# Interactions of spatial strategies producing generalization gradient and blocking: A computational approach

**DOI:** 10.1371/journal.pcbi.1006092

**Published:** 2018-04-09

**Authors:** Laurent Dollé, Ricardo Chavarriaga, Agnès Guillot, Mehdi Khamassi

**Affiliations:** 1 Institute of Intelligent Systems and Robotics, Sorbonne Université, CNRS, F-75005 Paris, France; 2 Defitech Chair in Brain-Machine Interface, Center for Neuroprosthetics, Institute of Bioengineering and School of Engineering, EPFL, Geneva, Switzerland; University College London, UNITED KINGDOM

## Abstract

We present a computational model of spatial navigation comprising different learning mechanisms in mammals, i.e., associative, cognitive mapping and parallel systems. This model is able to reproduce a large number of experimental results in different variants of the Morris water maze task, including standard associative phenomena (spatial generalization gradient and blocking), as well as navigation based on cognitive mapping. Furthermore, we show that competitive and cooperative patterns between different navigation strategies in the model allow to explain previous apparently contradictory results supporting either associative or cognitive mechanisms for spatial learning. The key computational mechanism to reconcile experimental results showing different influences of distal and proximal cues on the behavior, different learning times, and different abilities of individuals to alternatively perform spatial and response strategies, relies in the dynamic coordination of navigation strategies, whose performance is evaluated online with a common currency through a modular approach. We provide a set of concrete experimental predictions to further test the computational model. Overall, this computational work sheds new light on inter-individual differences in navigation learning, and provides a formal and mechanistic approach to test various theories of spatial cognition in mammals.

## Author summary

We present a computational model of navigation that successfully reproduces a set of different experiments involving cognitive mapping and associative phenomena during spatial learning. The key ingredients of the model that are responsible for this achievement are (i) the coordination of different navigation strategies modeled with different types of learning, namely model-based and model-free reinforcement learning, and (ii) the fact that this coordination is adaptive in the sense that the model autonomously finds in each experimental context a suitable way to dynamically activate one strategy after the other in order to best capture experimentally observed animal behavior. We show that the model can reproduce animal performance in a series of classical tasks such as the Morris water maze, both with and without proximal cues, which support the cognitive mapping theory. Moreover, we show that associative phenomena such as generalization gradient and blocking observed within the navigation paradigm cannot be explained by each learning system alone, but rather by their interaction through the proposed coordination mechanism. The fact that these experimental results have for a long time been considered contradictory while they could here be accounted for by a unified modular principle for strategy coordination opens a promising line of research. We also derive model predictions that could be used to design new experimental protocols and assess new hypotheses about complex behavior arising from the interaction of different navigation strategies.

## Introduction

Neurobehavioral evidence supports a prominent role for interactions between multiple anatomically distinct memory systems in the mammalian brain underlying the coordination of different behavioral strategies during learning (*e.g.,* [1]): A cognitive memory system, relying on a network comprising the hippocampus, prefrontal cortex and associative parts of the basal ganglia (i.e., the dorso-medial striatum), would mediate goal-oriented planning strategies; While a stimulus-response/habitual memory system, relying on sensorimotor parts of the cortex and basal ganglia (i.e., the dorso-lateral striatum), would in parallel mediate the progressive acquisition of routine strategies that would take over with overtraining [2–8].

Recently, a growing computational effort has been put forward to model the coordination of such behavioral strategies, with more and more computational models employing such a dual learning systems framework to account for changes in animals’ behavioral strategies between different stages of learning during the task [4, 9–12], as well as between different subparts of the action sequence or movement trajectory during the trial [9, 13, 14].

In particular, when dealing with instrumental conditioning experimental data, these dual systems models well explain animals’ tendency to alternate between initial flexible goal-oriented strategies, where the animal is hypothesized to use an internal model to plan and infer future consequences of action (model-based), and more automatic and habitual strategies at late stages of learning, where behavior is supposed not to rely on an internal model but rather on stimulus-response associations (model-free) (see e.g., [5, 15] for reviews). In the case of navigation paradigms, the model-based / model-free dichotomy has been found to better account for the diversity of navigation behaviors than the old classical distinctions between place and response strategies, or between allocentric / egocentric strategies [8]. Moreover, such a distinction provides a possible explanation of the distinct roles of the hippocampus and different subparts of the striatum during navigation [8, 15].

Nevertheless, how learning systems dynamically interact during navigation is still little understood. In particular, it is not clear how a unified coordination principle or mechanism can explain both cases of strategy competition (when a lesion impairing one strategy but leaving another one spared can produce an improvement in the animal’s behavioral performance, e.g., [16]) and cases of strategy cooperation (when two strategies together produce a better performance than one strategy alone, e.g., [17]). Existing computational models have proposed various criteria to coordinate multiple learning systems, but each criterion has been evaluated on specific experimental paradigms. For instance, system coordination has been proposed to depend on the uncertainty in the model-free system alone [11], relying on the strong assumption that the model-based system always has *perfect information*; Alternatively, some models have released the assumption of perfect information [18], but they still bias the coordination towards a default model-free control, which cannot explain why some actions always remain under model-based control even after training [4]. Other models propose a coordination that can also depend on uncertainty in the model-based system [4, 19], an approach that does not scale up to tasks involving a large number of states [20]; some authors have used a fixed coordination weight per individual [12, 21], which cannot account for dynamic changes in coordination strength along training; system coordination has also been proposed to depend on working-memory load [18, 19, 21] in human experiments where it is considered that accessing working-memory has a cost, and more recently in similar experiments in monkeys [22]. Few multiple-systems models have addressed rodent navigation data. The model proposed by [10] could solve a variety of navigation tasks in a simulated robot but employed fixed pre-learned behavior in the model-free system and did not perform formal comparisons between their simulated robot and experimental results in rats. The model proposed by [9] combined place-based and cue-guided learning systems, coordinating them by choosing at each timestep the system with the smallest reward prediction errors and the largest reward expectations. However, since the two learning systems are model-free, the model cannot account for flexible strategies enabled by model-based learning. In general, most previous computational models of rodent navigation have employed what is called a *Locale* strategy to account for place-based behavior [9, 13, 17, 23, 24], which learns place-action associations through model-free learning and can thus not account for model-based behavior.

**Fig 1:**
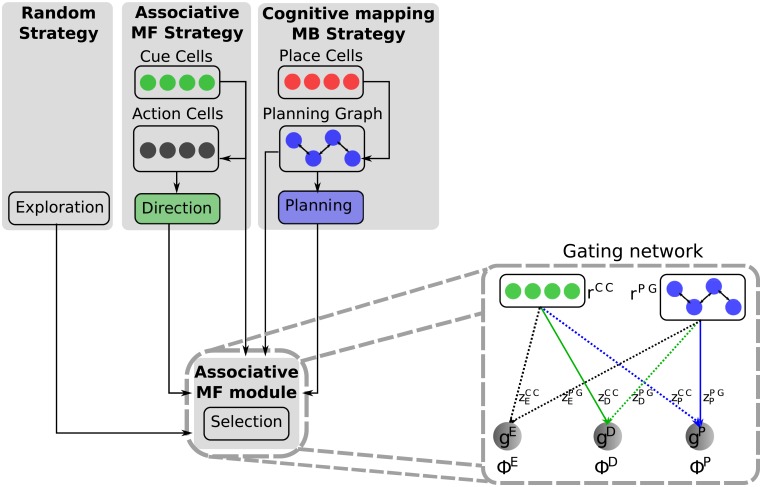
Model overview. The proposed computational model is composed of four main modules. The Direction module uses model-free (MF) reinforcement learning to associate visual information encoded by Cue Cells with propositions of oriented movements encoded by Action Cells. This results in an orientation proposed to the gating network; The Planning module is a model-based (MB) system which builds by Hebbian learning a topological Planning Graph with Place Cells and proposes to the gating network an orientation of movement reflecting the shortest path to find the reward; The Exploration module proposes random orientations of movement to the Gating Network. The selection between the outputs of the three modules is learned by a separate associative module through model-free reinforcement learning. The inputs of the Direction and Planning modules (CC and PG) are linked to the units in the gating network. The gating values *g*^*k*^ (*k* = E, D or P, corresponding to Exploration, Direction and Planning modules) are weighted sums of the input values *r*_*j*_ (*j* = CC or PG) with weights $z_{j}^{k}$. At each stimulated timestep, one among the modules is selected according to a winner-take-all scheme.

We previously proposed a computational model of navigation where a *gating-network* coordinates model-free and model-based systems with a commun currency: their measured instantaneous performance [14]. In the model the gating-network is an associative module which learns through model-free reinforcement learning which system is the most efficient in each location of the spatial and perceptual spaces, which implements a certain degree of hierarchy in learning [25]. This enables to *gate* the appropriate system at the right moment during performance, depending on input from place cells’ and visual cells’ activity (Fig 1). The fact that the gating-network learns to select which navigation strategy to follow based on model-free reinforcement learning is consistent with the hypothesis that the same selection mechanisms learned through dopamine reinforcement signals are employed in different striatal territories for movement selection, action selection, and strategy selection [5, 26–32]. Previous studies reported how the model could reproduce rodent experimental data in two specific navigation tasks. Nevertheless, the previous version of the model employed a hand-tuned mixture of Gaussians to model artificial hippocampal place cells with a fixed distance between place fields, and it is thus not clear how general these previous results were. Here, we extend this model by integrating a more realistic hippocampus model [33] and show how the mechanisms of the gating network within this new model can explain a wide range of experimental data during navigation paradigms involving spatial memory as well as associative phenomena such as generalization and blocking.

Specifically, we reproduce the classical reference memory experiment in the hidden water maze [34]; a delayed matching to place task [35]; cases of competition between strategies previously classified as cue-guided and place-based in a water maze [16]; a gradual competition between distal and proximal cues [36]; generalization gradient [37] and blocking [38]. In particular, we show that phenomena such as generalization gradient and blocking observed within the navigation paradigm, which to our knowledge have never been accounted for by computational models before, cannot be explained by each learning system alone, but rather by their interaction through the proposed gating network.

While the debate is still vivid in psychology and experimental neuroscience between the cognitive map theory and the associative theory of mammal navigation [39–44], our work highlights together with recent previous computational models that a variety of learning behaviors can result from a single coordination mechanism for the interaction between these two types of strategies. Moreover, while most previous computational models focus on mechanisms for the competition between learning systems, our work shows that a set of rodent navigation behaviors can be explained in terms of cooperation between systems. Finally, by proposing a common currency for learning systems coordination, our model can generalize to the coordination of N systems whose individual learning mechanisms may be of different nature. This could help predict behavior in paradigms involving more than two navigation strategies, which has so far rarely been experimentally studied.

## Results

The proposed computational model is composed of four main modules (Fig 1): an associative *Direction* strategy (D) which learns through model-free reinforcement learning to associate the perception of proximal cues within the environment with directions of movements; a cognitive mapping *Planning* strategy (P) which learns through model-based reinforcement learning a transition graph between different positions within the environment encoded in simulated place cells, and proposes directions of movement based on action plans towards the memorized goal position; and an *Exploration* strategy (E) which proposes random direction of movements. Finally, a *Gating Network* which learns through model-free reinforcement learning which strategy to select based on the system’s input (i.e., cue cues and place cells). The module dedicated to the model-based Planning strategy is itself composed of several modules dedicated to building hippocampal place representations through the integration of idiothetic and allothetic information from enthorinal cortex grid cells and sensorial cells to dentate gyrus place cells, and projections of pools of place cells to nodes of the cognitive graph within the prefrontal cortex module (S1 Fig). This results in more variability and plausibility of the simulated place fields compared to the the uniformly distributed Gaussian place cells that we used in the previous version of the model [14]. The detailed mathematical formulation of the model as well as parameter tables (S1 and S2 Tables) are given in S1 Text. We tested the model in several experimental paradigms with increasing complexity in order to show that the same associative principle for the coordination of the model-free *Direction* strategy and the model-based *Planning* strategy can account for a wide series of experimental data on rodent navigation. These simulations provide computational predictions about the way distal and proximal cues may compete for the control of behavior within such a modular architecture (examples of repartitions of cues processed by each of the modules are illustrated in S2 Fig). We also show that some experimental results previously accounted for by a model-free spatial strategy called *Locale* strategy (L) can be better explained in terms of the model-based *Planning* strategy (P) within this framework.

### Experiment I: Reference memory in the hidden water maze

**Fig 2:**
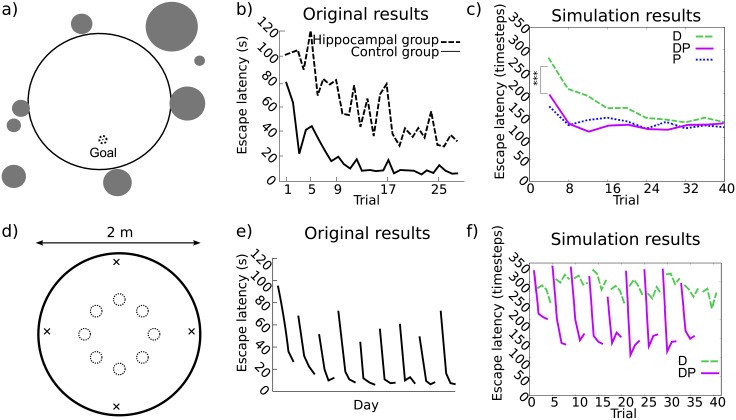
**(a-c) Experiment I: Original Morris water maze task by Morris et al. 1982** [34]**.** a) Simulated environment: gray disks represent schematized distal cues, the dotted circle represents the platform. b) Original results plotted as a learning curve of escape latency versus trials. c) Simulated results: Direction vs Planning. **(d-f) Experiment II: Delayed Matching Task by Steele and Morris 1999** [35]. d) Simulated environment: black crosses represent starting locations, dotted circles represent the possible platform locations. The distal cues around the water maze are here not represented for the sake of clarity of the figure. e) Original results plotted as a learning curve of escape latency versus days. f) Simulated results: Direction and Planning versus Direction only. D: Direction; P: Planning. *** corresponds to significance level *P* < 0.001.

One of the best known experimental paradigms, the Morris water maze show that intact rats are able to learn the location of a hidden, stable platform [34] (Fig 2a). In contrast, hippocampal-lesioned animals are impaired in such tasks as shown in Fig 2b.

While previous computational models have already reproduced these classical results (e.g., [17]), we present here new simulations with our model to show that it can also reproduce them (Fig 2c), but also to analyze which variants of the model fail to do so. The simulated hippocampus-lesioned model, where only the Direction (D) strategy is operational, shows significantly higher latencies to reach the platform during the first 10 trials than the full model, where both strategies (DP) are operational (Mann-Whitney test for non-matched paired samples, *p* < 0.001). In the simulations, when P and D strategies are available simultaneously, the gating mechanism learns to privilege the former (S3a Fig) which uses the configuration of distal cues to estimate the allocentric position of the platform and to plan a sequence of movements towards it. In contrast, the performance of an associative model with a D strategy only, where distal cues compete against each other, was impaired as is the case with hippocampal lesioned animals. Interestingly, our simulations predict that if the experiment is performed for a sufficient number of trials, animals with impairments in hippocampal processing (i.e., D strategy alone) should eventually reach the platform with performance that is not statistically different than control animals (i.e., DP strategies together). This is consistent with more recent experimental results showing that the blocking of hippocampal sharp-wave ripples oscillations, known to be important for memory consolidation, impairs performance in a spatial memory task but still spares a slow improvement in performance in the tested animals [45]. Moreover, our simulations predict that Striatum-lesioned animals (i.e., P strategy alone) should not be impaired in this task (Fig 2c). This is again consistent with more recent experimental results in the water maze where striatum-lesioned animals had non-different espace latencies than controls [16]. So far, these results are not novel compared to the large body of computational simulations of this experiments that have been previously done [17, 23, 24]. Nevertheless, it is interesting to note that many such models have reproduced these results using a model-free allocentric Locale (L) strategy in contrast to the model-based one used here.

Simulation of a variant of our model where the Planning strategy is replaced by a Locale can also reproduce the experimental results (S3b Fig). Nevertheless, the simulation results strikingly lead to different predictions: that the performance of Hippocampus-lesioned animals should never reach that of the control animals even after a large number of simulated trials; and that the performance of Striatum-lesioned animals (i.e., L strategy alone in S3b Fig) should also be impaired (but less) compared to control animals. Hence in this variant of the model, the two strategies together produce a better performance than each strategy alone, which reveals a potential collaborative interaction between strategies that will be discussed later. These predictions constitute possible ways to disentangle the two alternative models. However, here we argue that the ability of a model without model-based strategy to reproduce these results is mainly due to the stationarity of the task: the platform always remains at the same location, which can be easily learned by a model-free strategy. The next simulated experiments will show that in non-stationary cases, a model-based strategy is necessary to reproduce rats’ ability to adapt in a few trials to each change in the platform location.

### Experiment II: Delayed matching to place—Reaching a moving hidden goal

In an extension to the previous paradigm, the hidden platform was moved every session made of four trials (Fig 2d), thus allowing the animal to remember its position for a few trials once it has been found, but nevertheless requiring an adaptation to frequent goal location changes [35]. Experimental results show that escape latencies of intact animals increased on the first trial after the platfom is moved, but decreased quickly in the following ones (Fig 2e). Results of model simulations showed that this quick adaptation of behavior can be reproduced when a model-based Planning strategy is available (Fig 2f), but not with a model-free Locale one nor an associative Direction one (S4a and S4b Fig). The Planning strategy permitted the quick within-session adaptation observed in rats, while both Locale and Direction strategies were much slower at learning the platform location and hence did not display much within-session reduction in escape latency. Analysis of the evolution of the contribution of strategies within the full model shows that while the Direction strategy contributed to the model decisions of movement during the first simulated session, the model quickly learned to avoid using it during next sessions (S4c Fig). This explains why the performance of the full model shows smaller within-session reduction in escape latencies during the first session than during later sessions. At that stage, the model automatically learned that solving this task can be achieved through a combination of Planning and Exploration strategies. When the contribution of the Exploration strategy increased, such as during session #5, the model started the session with a lower escape latency because the model relied less on the Planning strategy at the first trial of the session and hence spent less time searching around the previous platform location. This suggests that an ideal combination of strategies in this type of tasks, once the structure of the task is learned by the model, would be to rely most of the time on the Planning strategy—to enable the quick within-session improvement in performance—while keeping a certain level of exploration to prevent the model from being stuck at the previous platform location. As we will see in Experiment IV, the gating network of the model can achieve this sort of cooperation between strategies—hence suggesting a way in which rats may do it—when the presence of an intra-maze cue enables the Direction strategy to be efficient at the first trial of each session. Before that, the next simulated experiment will illustrate a case where the use of an intramaze cue enables the Direction strategy to reach a good performance and hence to enter competition with the Planning strategy.

### Experiment III: Competition between cue-guided and place-based strategies

**Fig 3:**
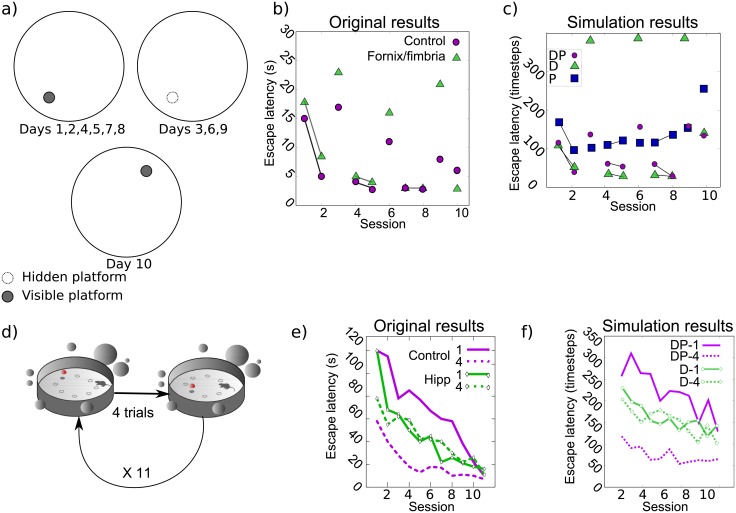
**(a-c) Experiment III.** a) Environment. b) Original results plotted as a learning curve of escape latency versus trials. c) Simulated results of the Planning + Direction group vs Direction only group. **(d-f) Experiment IV.** d) Simulated environment: gray disks represent distal cues, dotted circles represent the platform e) Original results plotted as a learning curve of escape latency versus trials. f) Simulated results of the Planning + Direction group vs Direction only group.

In this experiment, animals learned to reach a cued and stable platform, also identified by surrounding distal cues. During some trials, the cue was hidden, forcing the animals to learn its location also by distal cues (thus discarding the possibility of overshadowing—i.e., neglecting—distal cues because of the presence of the proximal one) [16]. In the last trial block (4 trials), the cued platform was moved at the opposite place, testing whether rats reach it following its spatial location or the cue (Fig 3a). In these trials, hippocampal-lesioned animals went directly towards the new cued platform position, as did half of the control animals—named *cue-responders*. In contrast, the remaining control animals—named *place-responders*—first swam towards the previous platform location (presumably following distal cues) and then went directly to the cued goal. This suggests a competition between both strategies taking place at these trials (Fig 3b).

Simulation results in this task with a previous version of our model have already been reported in [14]. That study focused on whether output actions in the model should have an egocentric or an allocentric frame. Here, using simulations relying on an allocentric frame for output actions, we address two questions: how the gating-network can manage inter-strategy competition in order to solve the task; what are the different experimental predictions raised when the model-free Direction strategy competes with a model-based Planning strategy versus a model-free Locale one.

As previously reported [14], the model can reproduce the experimental results both in the control case (when strategies P and D are available) and in the hippocampal lesion case (when only the D strategy is operational) (Fig 3c). Simulations reproduce the fact that control and lesion groups perform comparably in the trials where the intra-maze cue is visible (trials #1, 2, 4, 5, 7, 8) and during the competition trial #10, as well as the significantly larger escape latencies of the lesion group in trials where this cue is hidden (trials # 3, 6, 9). An interesting new prediction from the model is that lesions to the striatum (putatively impairing the Direction strategy while sparing the Planning strategy) would produce an intermediate performance (P group in Fig 3c). More precisely, the performance should not be impaired in the hidden cue case because the Planning strategy can still rely on distal cues to locate the platform. Nevertheless, the performance in the visible case should not be as good as the full model, indicating that the full model solves this case through a cooperation between strategies rather than by the Planning strategy alone. Such a cooperation is illustrated in S5d–S5h Fig where the trajectory produced by the agent during a given trial expresses a D strategy during the initial part of the trajectory and a P strategy later on. This enables to spend less time far from the new platform location by preventing the P strategy from driving the agent towards the previous platform, as is the case with the P model alone during the competition trial #10. This contributes to a better performance of the full model also in that case. Such a cooperation is nevertheless characterized by a strong dominance of the Planning strategy in the behavior of the simulated agents (S5a Fig). Separating selection rates by trial types clearly shows that the model manages to increase the contribution of the Planning strategy when the intra-maze cue is hidden (hence when the Direction strategy is inefficient) and to decrease it during the competition trial in order to reduce the time spent at the previous location of the platform (S5b Fig).

A second line of simulation results can be illustrated when repeated simulations with the same parameter-set enable the full model to exhibit behavior alike to the two distinct populations of experimentally observed rats: *cue-responders* and *place-responders* (S5c Fig). The most important prediction of the model in this case is that the behavior of both populations should at the same time reflect a dominance of each individual’s preferred strategy (Planning for *place-responders* and Direction for *cue-responders*), but in neither group this behavior results from the complete absence of the other strategy (S5c Fig). In our simulations, the Direction strategy still contributed to 20% of the choices made by the simulated *place-responders*. Conversely, the Planning strategy contributed to nearly 15% of the choices made by the simulated *cue-responders*. An important consequence of this feature is that individual simulated trajectories within the competition trial #10 reflect an alternation between movements guided by the three different strategies (Exploration included; S5d Fig). This illustrates a cooperation between strategies, the Planning strategy being the one which attracts the simulated agent towards the previous platform location in this case (obviously more strongly for *place-responders* than for *cue-responders*). These results suggest that even experimental situations of apparent competition between navigation strategies can be solved through different degrees of cooperation, the respective contribution of each strategy being dynamically adjusted by the model to achieve the task properly.

We performed additional simulations to analyze the case where the model-based Planning strategy is replaced by a model-free Locale strategy, as in previous computational models [9, 17, 24]. As before, the model shows an increased contribution of the spatial strategy (here the Locale instead of the Planning) during trials where the intra-maze cue is hidden, and a strong decrease in its contribution during the competition trial to avoid losing time at the previous platform location (S5e Fig). Nevertheless, these results reveal an overall increase in the contribution of the Direction strategy (S5f Fig). This can be explained as a mechanism of compensation for the lower flexibility of the model-free Locale strategy compared to the model-based Planning one. Interestingly, this version of the model is still able to reproduce the experimental results, both in the control and lesion cases (S5g Fig). Like in Experiment I, we argue that this is made possible because the platform location is stable during all trials except test trial #10. A different prediction from this version of the model is that, while *place-responders* should still perform mixed strategies relying on a cooperation between strategies, the involvement of the Locale strategy should be much weaker in *cue-responders*, resulting in frequent homogeneous trajectories only controlled by the Direction strategy (S5h Fig).

### Experiment IV: Gradual competition between distal and proximal cues

As in Experiment II, the platform was moved every four trials (a session), but in this paradigm a proximal cue indicating the position of the platform was held at a constant distance and direction from it [36] (Fig 3d). This was meant as a way to enable the Direction strategy to also solve the task on its own, and hence to trigger a fair competition between Planning and Direction strategies. Central questions addressed by the authors of the original study are whether hippocampal lesions would specifically impair a particular strategy and how this would bias the competition. Interestingly, they observed that both control and hippocampus-lesioned animals were able to (at least partially) learn the task since they both show a gradual improvement in performance, as illustrated by the decrease in escape latencies across sessions (Fig 3e). This session-by-session progressive improvement in performance converged to a point where both groups reached similar espace latencies in the last sessions. The important observation is that the two groups showed different performance characteristics within each session. Control animals were able to display a fast adaptation (i.e., within 4 trials) to the new position of the platform at each session. In contrast, hippocampus-lesioned animals did not show a significantly different performance between the first and the fourth trial of each session (Fig 3e). Strikingly, hippocampus-lesioned animals were nevertheless better than control animals at the first trial of the session. Further analyses reveal that this is explained by the tendency of hippocampus-intact animals to spend time at the previous platform location [36]. This experiment thus reveals a set of intrincate phenomena which support a dual learning systems approach: the hippocampus appears as necessary to enable fast adaptation to new platform location; Nevertheless, lesion of the hippocampus led to a reduction in the time spent around the previous platform location; In consequence, both groups were eventually able to learn the task. One important computational question is how the model should balance the competition/cooperation between the strategies to produce such a performance?

As for Experiment III, our previously reported results in this paradigm [14] focused on whether output actions in the model should have an egocentric or an allocentric frame. Here we show new simulations to (i) further analyze the balance between cooperation and competition mediated by the gating network, and (ii) to see whether a model-free Locale strategy could solve the task similarly to a model-based Planning strategy in the model.

The combination of Planning and Direction strategies in the full model can reproduce the behavior of intact animals in this experiment (Fig 3f). Emulation of hippocampal lesions in the model—leaving only the Direction and Exploration strategies spared—can reproduce the behavioral performance of the lesioned animals in this task: a better performance than the control group at the first trial of each session; and a lack of fast adaptation between the first and the fourth trial of each session; hence an impaired performance compared to controls at the fourth trial of each session. Interestingly, as observed in our previous work [14], an artificial lesion to the striatum in the model—leaving only the Planning and Exploration strategies spared—predicts an impaired performance in this task (S6a Fig): while the fast adaptation between the first and the fourth trial of each session is preserved, the striatum-lesioned model shows larger escape latencies than controls and hippocampus-lesioned models at the first trial of each session. This directly results from the tendency of the Planning strategy to be attracted by the previous platform location at the first trial of each session. We found this tendency to be stronger in the behavior of the simulated agent in the striatum-lesioned model (P) than in the full model (DP) (S6b Fig). Another interesting property of the striatum-lesioned model is that it does not show the progressive improvement of performance across sessions seen in the two other models, and which could be the signature of a slow model-free learning process (S6a Fig). This constitutes a strong prediction of the model which could be tested experimentally.

Importantly, simulation results of each strategy alone enable us to well decompose the overall behavior of the control group into two clearly distinct components: a model-based learning component responsible for the fast within-session adaptation and a model-free learning component responsible for the slow across-session adaptation. Importantly, these two components are clearly visible in the performance of the full model (Fig 3f). It is thus interesting that the model, which has been mainly designed to regulate the competition between navigation strategies—since it gives full control of the movement to a single strategy at each timestep –, learns to achieve some degree of cooperation between strategies so as to benefit from the advantages of each of them. Plotting the rate by which each strategy is selected by the gating network during the first and the fourth trial of each session reveals how the model learned to operate this cooperation (S6c Fig). The Exploration strategy was more selected during the first trial of the two first sessions until the gating network learned to decrease its contribution to the movement. In parallel, the gating network learned to decrease the contribution of the model-free Direction strategy which is not yet efficient at the beginning of the experiment, and to increase the contribution of the model-based Planning strategy which can lead to fast adaptation. Very interestingly, from the second session onwards, the gating network learned to progressively reduce the contribution of the Planning in parallel to the improvement of the Direction strategy with learning. This resulted in the simulated agent spending less and less time at the previous platform location (S6b Fig). After the eighth session, the Direction strategy is selected more often than the Planning strategy, because it is now sufficient to successfully solve the task. This is the explanation that the model offers relative to the hippocampus-lesioned group in the experimental data which eventually reached the same performance as the control group in the last sessions (Fig 3e). Finally, it is worthy of note that the selection rate of the Planning strategy not only decreases during the first trial of each session, but also during the fourth one (S6c Fig). This is because the input that the gating network receives only provides it with information about visual cues and activity in the planning graph (Fig 1). The gating network is thus not able to discriminate between the different types of trials. A prediction from this is that any learning occurring in one type of trial will affect behavior in the other type of trials, which contributed here in making the Direction strategy more prevalent in the behavior of the simulated agents in the late sessions.

We further evaluate the predictions of this approach when the model-based Planning strategy is replaced by a model-free Locale strategy. Interestingly, the learning of the Locale strategy is too slow to learn the new platform position within only 4 trials, making the performance of this version of the model at the fourth trial not better than the Direction group (S6d Fig). Hence, as it was the case in the previous experiment, the important message is that fast adaptations within a few trials experimentally observed in animals are more likely to be well accounted for by a model-based learning strategy than by a model-free one.

### Experiment V: Generalization gradient

The last two experiments presented here highlight the role that associative learning processes can play in navigation paradigms. In particular, both experiments were originally designed as attempts to experimentally contradict the cognitive mapping theory—relying on localization processes based on a constellation of distal cues—by showing that individual cues could induce associative phenomena previously observed in non-navigation learning paradigms to support the associative learning theory. These associative phenomena are generalization gradient and blocking effects, which we will define hereafter while showing at the same time that only a competitive interaction of the associative Direction strategy with others (e.g., Exploration, cognitive-mapping-based Planning strategy) can reproduce these effects, not an associative Direction strategy alone.

**Fig 4:**
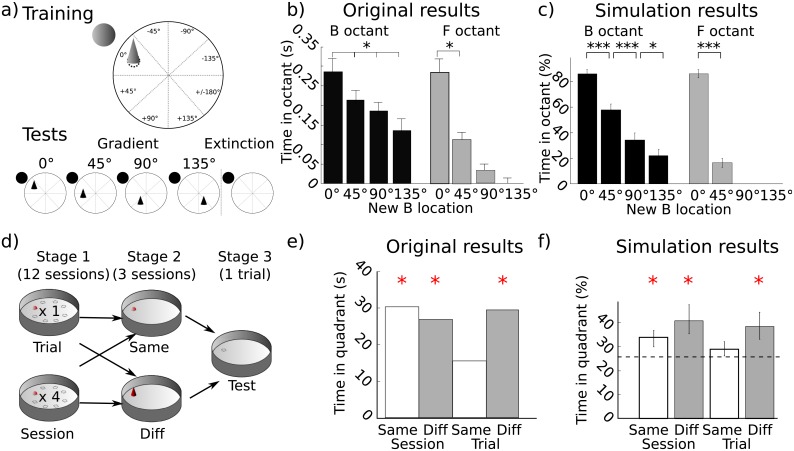
**(a-c) Experiment V.** a) Environment: gray disks represent distal cues, dotted circles represent the platform, gray cone represents the proximal cue b) Original results. c) Simulated results: time spent near the proximal cue (B) and the distal cue (F) by the full model combining Direction and Planning strategies. **(d-f) Experiment VI.** d) Environment: gray disks represent possible location of the platform, red dot and cone indicate cues. e) original results: escape latencies and time spent near the previous platform location during the test trial. f) Simulated results: time spent near the previous platform location in the Test trial by the full model combining Direction and Planning strategies. The horizontal dashed line represents chance level. *** and * correspond respectively to significance levels *P* < 0.001 and *P* < 0.05.

The spatial generalization gradient effect was studied by [37] in a navigation task involving a hidden platform under opaque water but marked by a proximal cue B, where a gradient of occupancy of the zone near cue B was recorded as this cue was progressively moved away from a distal cue F (Fig 4a). The authors expected a gradual loss of response to the proximal cue proportional to the distance increase. This decrease was supposedly due to the competition between cues—leading to a specific decrease of the proximal cue’s associative strength—rather than due to a competition between strategies.

The experimental protocol was composed of two training stages followed by one test trial. During Stage 1, a training of four sessions of eight trials was performed with two cues present, the proximal cue B (for *Beacon*) being initially close to the distal cue F (for *Frame of reference*). Stage 2 was composed of 10 sessions of nine trials each. In all sessions, eight of these trials were performed as in the previous stage (hereby termed *escape trials*). In the 9th trial of sessions 2, 4, 6, 8, and 10 (gradient trials), the platform was removed and the proximal cue B was rotated 0°, 45°, 90° or 135° from its original position (Fig 4a). This rotation was done either clockwise or counterclockwise, but the direction was kept constant for each animal. In the remaining sessions (1, 3, 5, 7, 9), the 9th trial was conducted with the F cue only, without the cue B nor the platform (extinction trials). These extinction trials were performed to reduce overshadowing of B by F, assumed to bias the generalization gradient.

The main experimental result shows that the greater the angle of the proximal cue B rotation during gradient trials, the less time the animal spent in the vicinity of this cue (i.e., a generalization gradient) (Fig 4b). In contrast, the occupancy of the area near the distal cue F did not exceed chance level. Thus, the proximal cue may have been overshadowing the distal one, and the obtention of the gradient suggests that the strength of the proximal cue was learned in an associative way. Nevertheless, during extinction trials rats occupied the octant F above chance level, hence revealing that behavior could still be under the control of the distal cue in the absence of the proximal cue.

**Fig 5:**
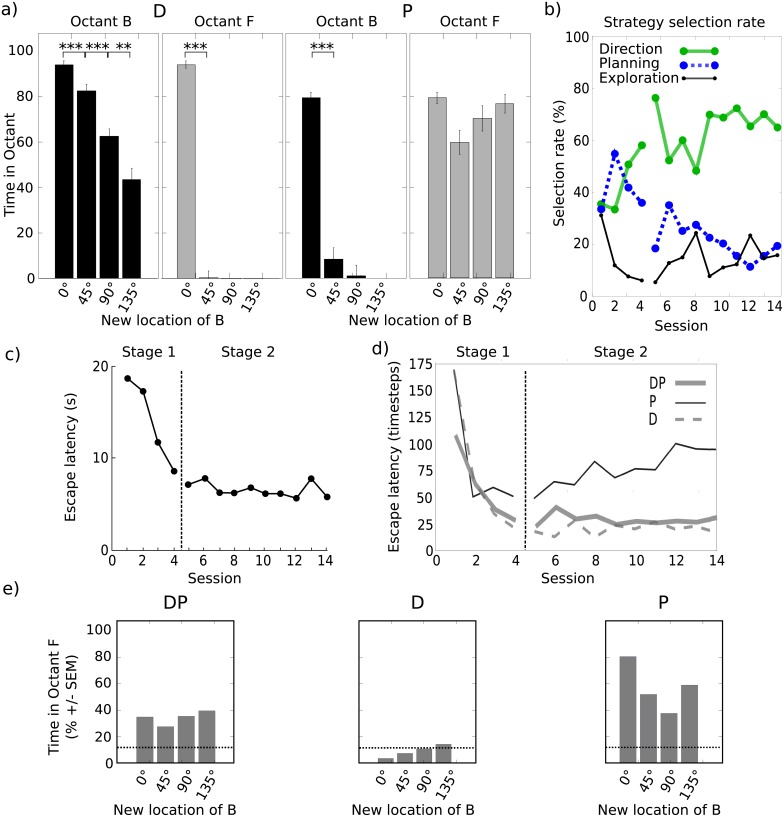
Experiment V: Detailed simulation results. a) Simulation results with either the Direction (D) strategy only (left) or with the Planning (P) strategy only (right) only. b) Strategy selection rate across sessions in the full model with all strategies (Direction, Planning, Exploration). c) Original experimental results with regards to the evolution of escape latencies during Stages 1 and 2 (adapted from [37]). d) Simulation results showing the evolution of escape latencies during Stages 1 and 2 in the full model, the model with D only and the model with P only. e) Occupancy rate in the F octant during the extinction trials in the three versions of the model.

Our model simulations suggest that only the competitive interaction between associative and cognitive mapping strategies could produce such effects. We found that Direction or Planning alone cannot reproduce the experimental results (Fig 5a). However, the modular approach allowing the selection among these two strategies in the full model was able to do so (Fig 4c). Analysis of the session-by-session evolution of the selection rate of each strategy reveals that the model could achieve this performance by progressively learning during Stage 1 that the Direction strategy is more efficient and accurate than the Planning strategy in this task and should thus be progressively more selected (Fig 5b). Indeed, plotting the escape latencies for the simulations with the Planning strategy alone shows a progressive improvement during Stage 1 followed by degradation of performance during Stage 2 (Fig 5d), which the model tried to compensate by selecting more and more the Exploration strategy instead of the Planning one (S7a Fig). This degradation of performance with the Planning strategy alone was not observed in the experimental data (Fig 5c) and only the simulations with the Direction strategy alone or with the full model (i.e., DP) could reproduce the performance during Stage 2 (Fig 5d). This suggests that the contribution of the Direction strategy was required to reproduce the characteristics of the learning process, and that within the Direction strategy the associative strength of cue B overwhelmed that of cue F (S7b Fig).

Nevertheless, simulations with the Direction strategy alone cannot reproduce the occupancy rates above chance level in octant F during extinction trials (Fig 5e). Chance levels were here obtained by simulating a *Chance* group, consisting of only one Exploration strategy, in the same conditions as the other groups. Only the full model and the Planning strategy alone could reproduce the animal behavior during extinction trials. Overall, only the full DP model could reproduce the ensemble of observed results in this experiment.

The selection rates of the strategies in the full model can give a further clue about the cooperation between Planning and Direction strategies which was employed to solve the task (Fig 5b). During Stage 1, selection rates indicate that both Direction and Planning strategies contributed to locate the platform. At the end of this stage, the gating network gave an advantage to the Direction strategy, but the Planning strategy remained selected at a rate above chance (36.4%). In the simulations without the Planning strategy, the Direction strategy was mainly helped by the Exploration strategy (averaged selection rate of 31%). At the beginning of this Stage, its performance was lower than in the full model—suggesting that the performance of the full model during these first trials was due to the cooperation between Planning and Direction strategies. This suggests that even if the Planning strategy was not the most efficient in this task nor sufficient to explain the experimental data alone, reproduction of rats’ performance by the full model still relies on the cooperation of the Planning strategy with the other strategies.

Importantly, the spatial generalization gradient effect in the full DP model was mainly due to the associative rules underlying the interactions between strategies rather than the associative rules within the Direction strategy itself (as the original authors hypothesized). S7c and S7d Fig detail strategy selections during gradient trials in groups DP (left) and D (right) by distinguishing the moments before the simulated agents had reached the octant B for the first time, and the moments after, when they occupied this octant and the other ones. Strikingly, as can be seen in the first column (“Before B”), the generalization gradient was not expressed until the simulated agents reached the octant B for the first time (light grey dashed line; no significant difference between test trials for 0°, 45°, 90° and 135°). The association between the proximal cue and the response leading to the goal was however well learned, as the simulated agents were able to reach the zone of the displaced proximal cue without any gradient. Yet the gradient itself was generated after, by the recruitment of other strategies when searching for the absent platform, i.e., without getting a reward (S7c and S7d Fig, right column “After B”, dark grey dashed line). This is also depicted by typical trajectories during gradient trials 45° and 135° (S7e and S7f Fig): octant B was rapidly reached with the Direction strategy and the Planning and Exploration strategies gave their contribution after, the former attracting the simulated agents towards octant F. These results contrast with the original authors’ hypothesis considering that the gradient resulted from a gradual loss of the associative strength of B during its learning. Unfortunately, the original experiment did not analyze the octant occupancy within gradient trials, but this prediction of the model would be easily verified.

### Experiment VI: Blocking

This experiment proposed another associative task [38] to investigate the expression of spatial blocking. This effect was proposed to depend on the amount of training with both distal and proximal cues, and on the change of the physical characteristics of the proximal cue. The hypothesis was that the blocking of the distal cues by a proximal cue would be due both to the presence of the same proximal cue during the experiment (which could be tested by the replacement by a different proximal cue in a different group of animals) and to the weak reliability of distal cues during training (which could be tested by changing the number of trials available to learn the position of a hidden platform based on distal cues).

In this experiment [38], four groups of animals are defined: Session-Same, Trial-Same, Session-Diff, Trial-Diff. The experimental protocol is decomposed into three different experimental stages (Fig 4d). In Stage 1, animals learned to find a cued platform (with a proximal cue A) in the presence of surrounding distal cues. For Session animals, the platform was moved every session (a session being composed of four trials). For Trial animals, the platform was moved every trial, so that rats did not anchor their learning process on the distal cues, contrary to groups Session. In Stage 2, the platform remained at the same location and was signaled either by the same proximal cue A (Same animals) or a different proximal cue B (Diff animals). Lastly, in Stage 3, the platform and its attached proximal cue were removed and the time spent near the previous platform location was recorded.

**Fig 6:**
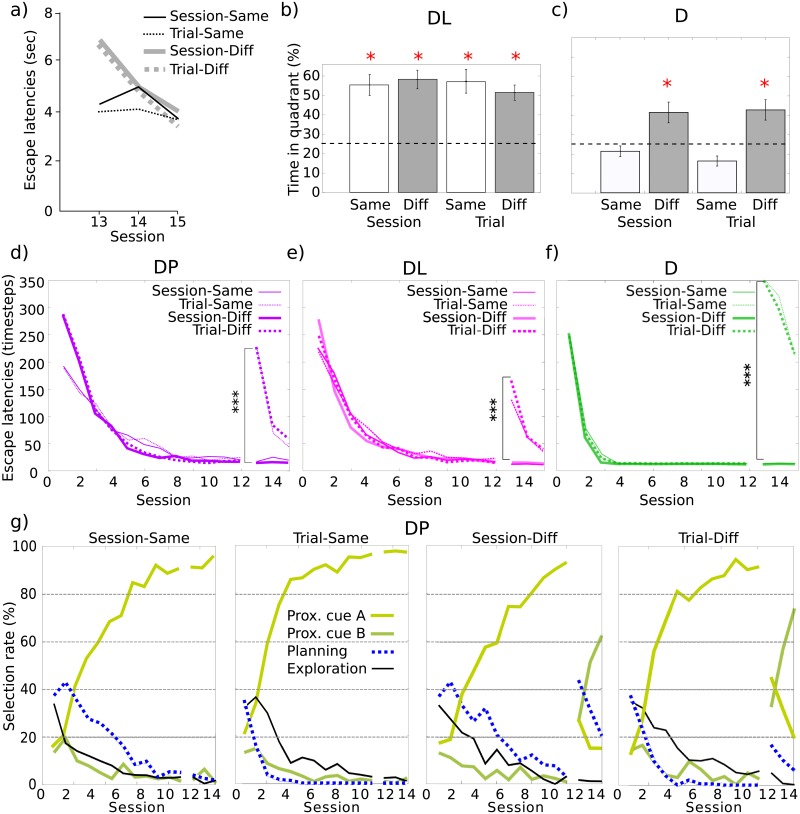
Experiment VI: Detailed simulation results. a) Original experimental results of [38] showing escape latencies during the three sessions of Stage 2. (b-g) Simulation results. b) Time spent in the quadrant containing the previous platform location when the model-based Planning strategy in the model is replaced by a model-free Locale (L) strategy. c) Time spent in the quadrant containing the previous platform location with the model containing only the Direction (D) strategy and the Exploration strategy. d) Escape latencies of the full model (DP). e) Escape latencies of the DL model. f) Escape latencies of the D model. g) Selection rate of each strategy in each condition of the task for the full model (DP).

The original experimental results showed the following main phenomenon: Only Trial-Same animals exhibited blocking (i.e., the time spent near the previous platform location lasted no more than chance level), whereas in other groups, animals spent more time near the previous platform location, demonstrating their learning of the platform location with the help of distal cues [38] (Fig 4e). The proposed explanations are that the two Session groups could not express blocking since, in Stage 1, distal cues were relevant to locate the platform. In contrast, distal cues were irrelevant for the two Trial groups during Stage 1, thus susceptible of being blocked. However, the change of proximal cue in Stage 2 in Group Trial-Diff prevented blocking, leaving Group Trial-Same as the only one to express a blocking phenomenon. A second important experimental result in [38] relates to the escape latencies of the animals. In the original article, the observed escape latencies were reported only for Stage 2. Groups Session-Same and Trial-Same showed no learning improvement (with respect to Stage 1), whereas groups Session-Diff and Trial-Diff expressed a re-learning of the association between the new proximal cue and the platform, resulting in larger escape latencies than groups Same during the first session of Stage 2 (i.e., Session 13) (Fig 6a).

Simulation of the full model (containing a Direction strategy D, a Planning strategy P and an Exploration strategy E) can reproduce these experimental results (Fig 4f): In the Trial-Same group, the selection module learned that the Planning was inefficient due to its poor performance in Stage 1 and was thus no more selected in the later stages (Fig 6g). In Diff groups, the change of proximal cue discarded the selection of the Direction strategies, thus leaving room for the Planning strategy to take place in Stage 3. And the Planning strategy was not discarded in Session conditions, because of its satisfactory performance in Stage 1. The full model could also reproduce the significantly different escape latencies during Stage 2 in the case of a different proximal cue B (Fig 6d). The model predicts that these escape latencies for the Diff conditions in the first session of Stage 2 should not be as high as those in the first session of Stage 1, thanks to the cooperation of strategies P and E which enabled some generalization between these two situations with a different proximal cue. This prediction can however not be confronted with the original article which does not show such comparison between Stage 1 and Stage 2.

We have also tested three other versions of the model in this task: A DL version where the model-based Planning strategy is replaced with a model-free Locale strategy; a D version where the Planning strategy is removed, leaving only the Direction and Exploration strategies; and a P version where the Direction strategy is removed. The latter completely fails to reproduce the experimental results because: (i) the time spent in the quadrant containing the previous platform location is significantly above chance in all conditions, unlike experimental results; (ii) the escape latencies during Stage 2 do not show an improvement and are significantly different between conditions Trial and Session, unlike experimental results (S8 Fig). A standard cognitive mapping approach is thus not appropriate to explain blocking in this context.

Interestingly, while the two other versions (models DL and D) can reproduce the escape latencies profile in Stage 2 (Fig 6e and 6f), as model DP also does, they lead to different predictions. The DL model predicts smaller escape latencies in the first session of Stage 2 compared to the first session of Stage 1, for the same reasons as the DP model. In contrast, the D model predicts even larger escape latencies since the Direction strategy starts Stage 2 with a performance lower than chance because of its synaptic weights resulting from learning during Stage 1 with a different proximal cue (S9a Fig). In addition, the D model predicts a quicker learning across sessions during Stage 1 than the two other models (Fig 6d–6f) because it is not polluted by the presence of an inefficient P or L strategy anchored on distal cues. Conversely, model P, but not model D, demonstrates a difference of performance between Session and Trial conditions (S8b Fig), and thus confirms that only the Planning strategy is able to learn within-sessions rather than across-sessions. This complementarity between the Direction and the Planning strategies is confirmed in model DP by the drastic improvement of performance between the first and the fourth trial of each session of Stage 1 in the Session-same condition compared to the Trial-Same condition (S9b Fig).

Most importantly, neither the DL model nor the D model can produce a blocking effect only in the Trial-Same condition (Fig 6b and 6c). Model D also showed a blocking effect in the Session-Same condition (unlike animals) because of the absence of a Planning strategy to rely on distal cues for the localization of the platform in this condition. S9c Fig illustrates how the DP model could avoid to express blocking in this condition by learning large weights and thus high confidence in distal cues used by the Planning graph (PG) when learning with the distal cues was possible for several trials (Session conditions). This results in the prediction that animals will hippocampal lesions should also show a blocking effect in the Session-Same condition, similar to model D.

Analysis of the behavior of the DP model during the test trial (Stage 3) provides further insights on the strategy coordination dynamics that may explain animal behavior in this task. We recorded the details of strategy selection in the model before reaching the quadrant of the platform and after, when the simulated agents occupied this quadrant and the others. Because in model DP the Direction strategy was preferred just before Stage 3, reaching the goal quadrant during the test trial mostly relied on this strategy (S9d Fig, 1st column of each condition), even if the corresponding proximal cue was absent, thus at random. The lowest selection rates of the Planning strategy (0.57%) were obtained during the Trial-Same condition. However, the Planning strategy was recruited after, when searching for the absent platform (S9d Fig, 2nd and 3rd columns). According to these results, in all conditions—and not only Trial-Same—did the model avoid to mainly rely on distal cues for reaching the goal quadrant in the absence of the proximal cue. Moreover, in all conditions—Trial-Same included—could the model quickly re-use distal cues after, which constitutes an interesting prediction of the model. Thus, in our simulations, the low occupancy rate of the goal quadrant in the Trial-Same condition could not be assumed to be due to a total blocking of the learning of distal cues—since distal cues were eventually used during the test trial –, but to a decrease in the confidence in these cues, acquired early in the experiment (as attested by S9c Fig, right). In model D, the blocking phenomenon was also expressed in the Session-Same condition, as expected by the authors but not observed in animals. A competition between strategies happened very early in the experiment, giving to the proximal cue a too high relevance during Stage 2 (S9a Fig). This reinforced the use of the Direction strategy in the Same conditions during Stage 3, leading to a longer time spent out of the goal quadrant (S9e Fig, 3rd column, Same compared to Diff conditions).

An important remaining question is whether alternative models employing a model-free Locale mechanism for the place-based strategy instead of the model-based Planning mechanism used here can also reproduce these results. Strikingly, unlike animals, model DL showed no blocking effect at all. This is because this task again involves a platform with a constant location, which gives an advantage to the Locale strategy over the Planning one by enabling the former to learn with more precision. As a consequence, while the Direction strategy is also the most selected in the DL model, the Locale strategy still contributes substantially to the behavior in the Trial-Same condition, hence preventing the blocking effect. Altogether, these results highlight that the complex mechanisms underlying the blocking effect in some conditions but not others can here not be reproduced by a purely associative model containing only a Direction strategy, but can instead be reproduced only by a modular approach which coordinates an associative strategy (here Direction) with a cognitive mapping strategy (here Planning). Only such a modular approach was in our simulations capable, like animals, of expressing both blocking and its absence.

## Discussion

In this work, we have presented a computational model for navigation paradigms combining a model-based Planning strategy, a model-free Direction strategy and a random Exploration strategy. The three strategies are coordinated by a gating network which learns in a model-free associative manner which strategy is the most efficient in each situation (i.e., depending on visual input and planning graph activity). The model could reproduce a set of behavioral and lesion data observed in navigating rodents in six different experiments (the main results are summarized in Table 1). The model can account for these data by achieving both competition and cooperation between strategies, which results in non-trivial behavior both within and across-trials. It is a striking feature of the model to be able, with a single coordination mechanism through a gating network, to produce both cases of competition between strategies, where lesion of one strategy leads to an improvement of performance, and cases of cooperation where two strategies together produce a better performance than each strategy alone. The model moreover permits a precise quantification of this cooperation/competition trade-off by plotting the evolution of weights assigned by the gating network to different strategies at different times across learning. This permits concrete predictions that could be tested experimentally. Importantly, these behavioral properties result from dynamic activation of different strategies. These dynamics were different from those obtained by a model composed only of model-free strategies, or of only a Direction strategy. The fact that these different variants of the model could not reproduce all the aimed experimental results highlights that a combination of navigation strategies of different nature is key to account for these experimental data. The simulations moreover yield a series of predictions which could be tested in future experiments to further assess the model (Table 2). We also summarize predictions raised by the alternative model DL, which only relies on model-free strategies, in order to guide future experiments that aim at further comparing the two models (Table 3).

**Table 1:** Summary table of the different models that could reproduce each experiment. D: Direction strategy alone (combines different Direction modules in the case of multiple cues); P: Planning strategy alone; DP: combined Direction and Planning strategies; DL: combined Direction and Locale strategies.

Exp	Reference	Main phenomenon	D	P	DP	DL
I	Morris et al., 1982 [34]	Reference memory in the hidden water maze	No	Yes	Yes	Yes
II	Steele et al., 1999 [35]	Delayed matching to place—Reaching a moving hidden goal	No	Yes	Yes	No
III	Devan & White 1999 [16]	Competition between cue-guided and place-based strategies	No	No	Yes	Yes
IV	Pearce et al. 1998 [36]	Gradual competition between distal and proximal cues	No	No	Yes	No
V	Rodrigo et al. 2006 [37]	Generalization gradient	No	No	Yes	
VI	Roberts et al. 1999 [38]	Blocking	No	No	Yes	No

**Table 2:** Summary table of the main predictions raised by simulations of the DP model. Same abbreviations as in Table 1.

Exp	Model	Prediction
I	DP	If the experiment is prolonged, hippocampus-lesioned animals should eventually reach the platform with performance that is not statistically different than control animals.
I	DP	Striatum-lesioned animals should not be impaired in this task.
II	DP	Hippocampus-lesioned animals should be slower at learning the platform location and should hence not display much within-session reduction in escape latency.
II	DP	Inactivation of the striatum during late sessions should not affect performance.
III	DP	Striatum-lesioned animals should produce an intermediate performance between control and hippocampus-lesioned animals: no impairment in the hidden cue case; lower performance than controls in the visible case.
III	DP	Transient inactivations of the striatum when the intra-maze cue is hidden should barely affect performance.
III	DP	Transient inactivations of the hippocampus during the competition trial should reduce the time spent at the previous location of the platform during the first sessions, and barely affect performance during subsequent sessions.
III	DP	The behavior of both cue-responders and place-responders should reflect a dominance of each individual’s preferred strategy, but in neither group should this behavior result from the complete absence of the other strategy.
IV	DP	Striatum-lesioned animals should show: a spared fast adaptation between the first and the fourth trial of each session; larger escape latencies than controls and hippocampus-lesioned animals at the first trial of each session; no progressive improvement of performance across sessions seen in the two other groups, and which could be the signature of a slow model-free learning process.
V	DP	During gradient trials, animals should rapidly reach octant B with the Direction strategy, while the Planning and Exploration strategies would give their contribution after, the former attracting the simulated agents towards octant F. The generalization gradient should thus not result from a complete loss of the associative strength of proximal cue B during learning.
VI	DP	Escape latencies for the Diff conditions in the first session of Stage 2 should not be as high as those in the first session of Stage 1, thanks to the cooperation of the Planning and Exploration strategies which should enable some generalization between these two situations with a different proximal cue.
VI	DP	Hippocampus-lesioned animals should show larger escape latencies than controls; a quicker learning across sessions during Stage 1 because they should not be polluted by the presence of an inefficient spatial strategy anchored on distal cues; and a blocking effect in the Session-Same condition in addition to the Trial-Same condition.
VI	DP	In all conditions—Trial-Same included—should the animals be able to quickly re-use distal cues after reaching the goal quadrant in the absence of the proximal cue, when searching for the absent platform.

**Table 3:** Summary table of the main predictions raised by simulations of the DL model. Same abbreviations as in Table 1.

Exp	Model	Prediction
I	DL	The performance of Hippocampus-lesioned animals should never reach that of the control animals even after a large number of simulated trials.
I	DL	The performance of Striatum-lesioned animals should also be impaired (but less) compared to control animals.
II	DL	Both control and striatum-lesioned animals should be slow at learning the platform location and should hence not display much within-session reduction in escape latency.
III	DL	The behavior of place-responders should rely on a cooperation between strategies, while the involvement of the spatial strategy should be much weaker in cue-responders, resulting in frequent homogeneous trajectories only controlled by the Direction strategy.
VI	DL	There should be smaller escape latencies in the first session of Stage 2 compared to the first session of Stage 1, for the same reasons as the DP model.

Several previous models have used a Locale strategy [9, 23, 24, 46, 47], which associates places to movements without really building a cognitive map (no topological graph; see detailed comparisons in [8, 48]). Here we have shown that the Planning strategy better explains behavioral results observed in protocols involving frequent changes of goal location, because a model-based strategy is more flexible than a model-free one [4]. We also found that the Locale works well when the goal location is stable, suggesting a possible co-existence of the two strategies in a modular architecture. Such a co-existence has been previously discussed in [8], arguing that model-free learning processes involved in the Locale strategy could take place in the dorsolateral striatum, while model-based learning processes involved in the Planning strategy could take place in the hippocampus and prefrontal cortex. The possible involvement of the dorsolateral in a model-free place-based strategy is consistent with electrophysiological recordings showing that activity in the dorsolateral striatum correlates with place when the task requires knowledge of spatial relationships [49]. Inactivations of these different regions could be a way to test the different predictions raised in this manuscript relative to Planning versus Locale strategies.

Several previous models have already proposed a coordination of model-based and model-free reinforcement learning mechanisms to account for various rodent behavioral data [4, 11, 12, 18] and could thus be considered as possible candidates to model the experiments addressed here. Nevertheless, the Lesaint model [12] proposes a fixed coordination of MB and MF through time: each individual has a specific weight attributed to each system determining its contribution in decision-making. The models proposed by Daw [4], Keramati [11] and Pezzulo [18] do incorporate a dynamic coordination of MB and MF, based on uncertainty. Nevertheless, these models were designed to account for the sequential shift from initial goal-directed behavior to habitual behavior after overtraining, explaining the insensitivity in the latter case to outcome devaluation, which is a specific case of the questions addressed here. In Experiment IV studied in the present work, animals progressively learn to reduce their use of the cognitive mapping strategy, which we explain in the model by the fact that the gating network learned to use less and less this strategy at the first trial of each session to avoid being attracted by the previous platform condition. The Daw and Keramati models should in principle not be able to explain this because the uncertainty associated to the MB system should be lower and lower sessions after sessions, while uncertainty in the MF system should remain high because the platform changes location every four trials. Besides, the Pezzulo model biases its system coordination towards a default model-free control, which cannot explain why some actions remain under model-based control even after training, as argued in [4] and as observed in several experiments considered here (*e.g.*, S3a, S4c, S5a, S7c–S7e, S9d Figs). Moreover, the fact that the gating-network of our model learns to coordinate strategies (which is not the case for these three other models) also enables the model to learn to increase the contribution of the Exploration system when necessary (S5b Fig), which corresponds to a dynamic exploration rate which is absent from these other models. Moreover, the generalization gradient in Experiment V is produced by the model at the level of the associative rules within the gating network (thus at the level of strategy coordination) rather than at the level of associative rules within the model-free Direction strategy itself. The Daw, Keramati and Pezzulo models proposed a coordination criterion which depends on instantaneously measured signals (i.e., uncertainty) rather than on learned signals (i.e., their models cannot learn that strategy X is efficient in a particular part of the environment while strategy Y is efficient in another part), hence they cannot reproduce this effect. Nevertheless, these models account for a variety of other experiments involving outcome devaluation, contingency degradation as well as hippocampal off-line replays, which our model does not address. Thus it would be particularly interesting in future work to study if combining mechanisms from all these models can account for a wider array of experimental data.

Most animal experiments have aimed at distinguishing between only two strategies (place-based versus associative), without subtly distinguishing subtypes of these two categories. Our model enabled to show that different subtypes of place-based strategies (i.e., planning versus locale) are more efficient/relevant depending on the protocol. Similarly, we have previously illustrated how different subtypes of response strategies (taxon, direction) which differ in the frame of reference for actions (resp. egocentric and allocentric) can also display complementary behavioral properties [14]. Together these computational results predict that new elaborated protocols should permit to isolate more than two concurrent strategies (for instance, planning+locale+direction or planning+direction+taxon). The common currency proposed here enables in principle to coordinate any number of strategies of any different nature, because the model just needs to be able to evaluate their current performance in different *states* of the task. Moreover, these various subtypes of strategies should engage different parallel memory systems (for instance subterritories of various cortico-striatal loops, depending on the input-output of these territories and their respective learning mechanisms). This predicts that specific lesions of these subterritories should affect only particular subtypes of strategies.

The present computational results have important implications relative to the debate between the *cognitive mapping* theory and the *associative* theory of spatial cognition in mammals [50, 51]. These two theories propose alternative mechanisms to explain spatial learning. According to the *associative* theory, spatial learning is dependent of a single type of mechanism—abundantly studied within the framework of classical and operant conditioning—by which a new response is incrementally acquired by the association of a stimulus and a reward [52–55]. In the associative paradigm, stimuli or group of stimuli available in the environment are assumed to compete to control animal navigation.Those which are not favored by this competition are not going to contribute to the achievement of the task. The *cognitive mapping* paradigm rather attests the existence of non-associative spatial rules (i.e., not incremental, independent from reward), in which all cues participate to develop a spatial representation [56]. This theory has received a strong support from the discovery of hippocampal place cells [57], which enables the animal to quickly build a reliable spatial representation of their environment [58], independently from the reward (latent learning). The debate between the two theories is still vivid in that the *cognitive mapping* paradigm is not able to explain blocking or overshadowing effects, and since the actual existence of such “cognitive map” enabling animal and humans to plan shortest paths or shortcuts aroused and still arouses controversies [39, 40]. On the other hand, opponents to the *associative* theory highlight a number of experiments failing to display overshadowing between proximal and distal cues [41, 42] or revealing potentiation between cues during attempts to look for spatial blocking and overshadowing (for a review, see [43]).

We have tried to show here that these theories could however be reconciled by a *modular* paradigm which proposes that both kinds of mechanisms may cohabit in distinct neural systems and may be learned in parallel [44]. Indeed, a large amount of studies have shown that inactivation of specific neural zones in rodents selectively impair only part of their navigational capacities [2, 44, 59–69]. The modular approach is also strengthened by several experimental procedures that have shown animals shifting from one type of spatial strategy to another one, either within a navigation trial, or as learning takes place across sessions [36, 44, 70–78]. This suggests the existence of mechanisms ruling the *selection* among navigation strategies in distinct neural structures from those which learn each strategy. In support of this view, lesions of prelimbic and infralimbic areas of the medial prefrontal cortex prevent the shift of a place-based strategy towards a cue-guided one but does not prevent the strategies themselves to be learned or displayed [79]. Similarly, lesion and electrophysiological studies of the ventral striatum suggest an evaluative role of the structure, important for initial learning and flexibility, but not necessarily a substrate for learning a specific navigation strategy (e.g., [62, 80]; see more thorough discussions in [7, 8, 81, 82]). The computational model proposed here constitute a refutable proposition concerning the mechanisms that may underly such a modular organization combining associative and cognitive mapping memory systems.

Several criticisms of the cognitive mapping theory have argued against the assumption that a global topographical representation (i.e., a cognitive map) exists and this information is available at all times during training. Whether this is a valid assumption and whether real rats benefit from such a representation is open to debate. However, it is important to emphasize that our computational model does not assume that a global map is learned. The mapping mechanism that we used rather focuses on the representation of areas that have been extensively visited [83], and it leads to local, partial and sometimes approximative maps that can produce suboptimal planning behavior in embedded, noisy tests [84]. Such a mechanism is supported by the observation that successful “planning” of trips does not necessarily depend upon a global representation (see, e.g., [85]). Moreover, a number of studies over the past 20 years have provided empirical evidence of local, non-global maps (e.g., [86–88], the first one providing clear evidence that non-global (at the very least) representations are involved in rodent spatial navigation in the water task). Related to this, recent work, both behavioral and physiological, has emphasized the important distinction between local boundary and distal landmark control [89–92].

These results also have important implications for the understanding of the coordination of learning and decision-making systems in humans, beyond spatial navigation. While we focused here on the modeling of experimental data in rodents for consistency, the coordination of model-based and model-free learning principles has also been highlighted in humans during instrumental learning tasks [93]. Moreover, cognitive mapping models have implications beyond spatial navigation, including roles in information contextualization [94], navigation between conceptual relationships in a manner similar to that of space [95], mapping of social relationships [96], and more generally in the integration of memories to guide future decisions [97]. Within this framework, an important question relates to the nature of the interaction between brain networks that underlies these cognitive functions. As mentioned above, previous contributions have emphasized the role of different parts of the striatum in different types of learning [5, 7, 8], the hippocampus being in a position to provide transition information between places for the building of model-based information in the medial prefrontal cortex and more ventromedial parts of the striatum [8]. Interestingly, studies in humans have demonstrated the recruitment of the striatum during learning with immediate feedback in a probabilistic learning task, and increased activation of the hippocampus with delayed feedback [98, 99]. Strikingly, in these tasks human subjects with Parkinson’s disease—whose striatum is known to be degraded—were impaired in learning from immediate but not delayed feedback. Such results appear consistent with the separation within the model between dorsolateral striatum-dependent model-free learning and hippocampus-prefrontal cortex-dependent model-based learning. Nevertheless, the precise role of different subparts of the prefrontal cortex in these learning processes is probably more difficult to disentangle. One currently attractive theory proposes that the orbitofrontal cortex participates to the learning of relationships between states within the model-based system, which in humans can also be useful to learn cognitive maps of non-spatial tasks [100]. In contrast, hippocampal projections to regions homologous to the dorsolateral and anterior cingulate prefrontal cortex are thought to play an important role in performance monitoring, with increased between-regions coherence upon task learning [101]. Such a process could relate to the performance monitoring mechanisms that underlie systems coordination within our *gating-network*. Nevertheless, more investigations would be required to further test the hypothesized roles of different prefrontal cortex subregions with respect to the different computations in the model.

The proposed coordination of learning systems also offers an opportunity to discuss about the possible role(s) of dopamine in mediating memory formation. Here, to be conservative, one could argue that the only role of dopamine on which to postulate relates to the production of phasic model-free reinforcement signals to update action values [102]. Following previous work on the combination of model-based and model-free learning in Pavlovian conditioning [12], we could further predict that dopamine blockade would only impair model-free navigation strategies, but not model-based ones, thus predicting similar behavior to the one shown through simulations of the Planning system alone. Such a prediction, specific to the navigation domain, could be interesting to experimentally test in order to further assess the model. Nevertheless, dopamine is known to play a role beyond the learning of action values based on reinforcement: For instance, it has been shown that dopamine contributes to the successful binding between experiences that are separated in time [103], which have been interpreted in terms of inference-based processes at the time of generalization. While dopamine reinforcement signals hypothesized to subserve model-free learning in our model could in principle slowly produce some binding between delayed events, notably through the association of reward values to stimuli and places that precede it, true off-line inference in the model relies on model-based processes (which enable action planning through a tree-search process [104]). Hypothesizing that dopamine plays no role in model-based learning [12] would at first glance fail to explain the coupled changes in learning-phase activity between the hippocampus and the dopaminergic system during information binding [103]. Nevertheless, the possibility to include in the model some off-line replay mechanisms—which permit another form of systems cooperation through the transfer of knowledge from model-based to model-free [105]—could be a promising extension of the model to explain off-line hippocampus drives over model-free dopaminergic learning signals [106] without using these signals for model-based learning per se.

Finally, some simplifications and limitations of the present model should be stressed in order to highlight possible ways to improve it. A first criticism that can be raised against the model presented here is the important number of parameters needed. Some of them need to be tuned differently according to the experiment (S2 Table). As a consequence, this can weaken the explanatory power of the model, that could be seen as an unnecessarily complex *mixture of experts* [107], where each strategy is considered as an expert whose selection becomes then irrelevant. In order to tackle this issue, we limited ourselves to two free parameters only, and changed their values within constrained boundaries. These parameters are the model-free learning rates of, respectively, the Gating Network and the Direction strategy—thus 2 parameters among a total of 18. The neurobiological meaning of such parameters (inherent to any RL model) has been investigated [108], and could account for motivational levels like, for instance, a stress induced by the experiment [109]. Moreover, it is not unreasonable to consider that animals may have changed their learning rates between task conditions [110]. While adding mechanisms to dynamically adapt learning rates based on some measures of the statistics of each task (such as reward volatility as done in [110]) would have added unnecessary complexity with respects to the phenomena investigated here—making the interpretation of our results more difficult –, one particularly interesting continuation of this work could consist in modeling neural systems responsible for such task monitoring and motivational effects and their influence on learning rates.

A second important limitation of the model is that it does not address the question of which precise model-free learning mechanisms should be employed. Instead, it rather focuses on the comparison at the global level between learning properties of model-based and model-free families of reinforcement learning algorithms [104]. Thus here we have not tested different types of model-free (MF) learning algorithms (*e.g.*, Q-learning, Actor-Critic, SARSA). Comparing these different MF algorithms is particularly important when examining the precise profile of neural activity in different brain regions, as done by Hagai Bergman’s group and Geoff Schoenbaum’s group [111, 112] (see [113, 114] for extensive discussions) who investigated which of these different algorithms could best explain dopamine neurons’ phasic activity in instrumental learning tasks. Such an analysis goes beyond the present work and extensions of the model would be required to account for this. Nevertheless, in previous work, we have shown that these precise MF learning algorithms do not make very different predictions in terms of behavioral adaptation [115, 116], the behavior of animals in such tasks instead appearing to also rely on a more flexible MB learning algorithm. This is why the present study focuses on the comparison between learning algorithms of different natures (MB, MF, random exploration) to account for animal behavior.

In summary, we presented a new computational model of navigation that successfully reproduced a set of different experiments involving cognitive mapping and associative phenomena during spatial learning. The fact that these experimental results have for a long time been considered contradictory while they could here be accounted for by a unified modular principle for strategy coordination opens a promising line of research to systematically assess computational predictions of this type of modular computational models of navigation. This type of model can also be used to design new experimental protocols and assess new hypotheses about complex behavior arising from the interaction of different navigation strategies. In parallel, such models could contribute in translating important inspiration from animals’ behavioral flexibility to autonomous agents having to display fast adaptation to rapid changes in the environment from a small amount of data, a paradigm which has been called *micro-data learning* [117], by opposition to big data learning where the data perimeter is already known in advance. The computational work presented in this manuscript thus highlights the importance of cross-talk between disciplines interested in biological and artificial cognition to contribute to a better understanding of brain and behavior.

## Materials and methods

### Model description

The model is sketched in Fig 1 and described in more details in S1 Text.

#### Associative cue-guided strategy

In the model, the Direction module implements the associative approach in the model as a cue-guided strategy, in which each environmental cue has to be associated to the appropriate actions in terms of directions of movement. In our model, the Direction module works in an allocentric directional reference frame, corresponding to a *heading vector strategy* [36]. This strategy would be supported by a head-direction cells’ network involving the anterodorsal nucleus [118], so that all directions are given with respect to the zero direction of that allocentric reference frame (fixed upon the first entry to a novel environment).

The Direction module is composed of two populations of simulated cells. The first population comprises hypothetic *Cue Cells* (CC), that code for external (visual) stimuli, while the second population is composed of hypothetic *Action Cells* (AC), representing different directions of movement [14, 119]. Each AC *i* receives input from all CCs and codes for the movement orientation $\phi_{i}=\frac{2\pi i}{N_{AC}}$. Its activation represents the strength of moving in the corresponding orientation and is computed as a sum of CC activation weighted by the synaptic links between CC and AC.

Learning of these weights is performed by the Temporal Difference (TD)-based Q-learning algorithm [104], a classical associative reinforcement learning. Values of synaptic links between CC and AC are incrementally updated relatively to the amount of the “reward prediction error”, the difference between the expected reward (as computed by the value of AC) and the actual reward R (equal to 1 when the goal is reached, 0 otherwise), so that actions that lead to greater reward values than expected will be more likely to be chosen the next time, whereas actions that lead to less reward than expected will be less likely to be chosen.

#### Cognitive place-based strategy

The Planning strategy is implemented by means of a module containing a topological map based on a population of simulated hippocampal place cells (PC) [83]. This representation, independent of the goal location information, is assumed to be built in a training session and allows for the use of graph search algorithms to find the shortest path to the goal [120]. In our simulations, Place cells are learned using a biomimetic model combining idiothetic information (odometry, given by simulated grid cells) and visual input provided by the CC population [33, 119]. Connections between these inputs and place cells are randomly initialized before the learning phase. PC building consists then in a Hebbian learning of a pool of 1000 cells, while the agent wanders around in the environment.

This process yields a partially sparse representation of the environment. However, it is assumed that a sparser representation is needed to allow learning in the graph [83]. For this reason, a pool of 100 Graph Cells (GC), receiving Place Cells as inputs, is used to encode the Planning Graph (PG). The same Hebbian learning is used for the synapses between PC and GC. Two GC are then linked if it is possible for the agent to move between the locations represented by them without encountering a third GC in between. At the end of the learning phase, the resulting output consists of a topological Planning Graph, allowing the use of a planning algorithm in order to find the shortest path from the node where the agent is currently located to the goal node [83]. Then the input of the Planning module consists in a graph integrating odometry and several environmental cues, contrasting with the input of the Direction module.

Another difference with this module is the manner the topological graph is learned, independently from the reward (i.e., a link is created between locations A and B if it is possible for the agent to move directly from A to B, no matter the amount of reward it receives either in A or in B). As a consequence, if the goal location is changed, the Planning Graph does not need to be relearned. Thus a single trial in which the new goal is found is enough to propagate goal information in the graph and to determine the new shortest path to reach the goal.

#### Random strategy

Contrary to other biomimetic navigational models using learning algorithms (e.g., [9, 121]), we use an explicit module providing random directional actions (corresponding to an exploratory behavior in the sense of reinforcement learning). The first advantage of having an exploratory module is that a single mechanism provides exploratory actions on the basis of which all modules are updated as if they had selected the executed action. A second advantage, which is a corollary of the first one, is that exploration can be the result of any type of decision mechanism: either purely random decisions, as adopted here for simplicity and parsimony; or more complex exploratory movements leading the agent along the walls (thigmotaxis) or near a new object of interest in the environment [122]. Indeed it should be noticed that randomness is not sufficient to describe exploratory behaviors in real animals. Nevertheless, such an independent exploration module is biologically plausible, the existence of such specific module being supported in a recent review [123].

#### Associative selection of strategies: Gating network

During goal learning, the model has to select which of the modules (i.e., strategies) takes control over behavior, i.e., chooses the next action. When several Direction modules are implemented in the model (because of the presence of several environmental cues) together with a Planning and an Exploration module, a first selection is made among Direction modules, then between the winner and both other modules.

The gating network learns to select among the modules on the basis of a “common currency” that allows the comparison of strategies using different learning algorithms [119]. This currency is defined as the *orientation* of movement proposed by each module. The existence of a common currency is supported by the role of separate brain regions specialized in the selection of navigation strategies, as reviewed in the introduction. The biological plausibility of using orientation as the actual currency will be further discussed. It is chosen here for modeling constraints: orientations will eventually be proposed by all navigation modules, even if they do not share any learning principle. Then the selection network can work with any kind of modules, being deterministic (like Direction and Planning) or random (like Exploration).

The gating network consists of units, which activation *g*^*k*^, called “gating value”, expresses the expected reward the agent can get if one specific module *k* is selected (Fig 1). The input to the units in the gating network is provided by the activations of the Cue Cells population and of the nodes of the Planning Graph. Similarly to the learning in the Direction module, the connection weights for the Direction and Planning gating values are randomly initialized and adjusted using a TD-based Q-learning algorithm. However, contrary to a classical TD-learning algorithm where only the chosen action is updated, all modules are updated, given their proposed orientation of movement: the closer an orientation is from the selected one, the higher the corresponding strategy will be reinforced. In contrast, two strategies that proposed two opposite orientations will have opposite reinforcement. This can be seen as a discrete version of a generalization mechanism [124].

The selection between modules is performed at each time step, unless the Exploration module is chosen, in which case a persistence mechanism is used and the orientation chosen by this module is taken during three subsequent time steps. This was done to avoid the agent being stuck in a particular location due to random weight initialization. Since exploration actions are pseudo-random, as learning goes on, their weights are expected to *decrease* with respect to strategies that direct the agent towards the goal, since the gating network assigns higher weights to strategies that consistently maximize reward.

Thus, by its associative mechanism, the module of selection favors competitive interactions between strategies: if the gating value of one strategy remains greater than the value of another one, the first would be able to block or overshadow the expression of the other. However, as the strategies could be sequentially selected within a trial, they could also cooperate (giving a better global performance than the performance provided by each), one being able to supply the temporary weaknesses of the other.

#### Simulations

The agent was simulated as a disk of 15 cm diameter, with a panoramic visual sensor (360°). Simulated environments and procedures were built respecting the original experimental paradigms. In consequence, the agent was allowed a maximum of 350 timesteps per trial. If it failed to find the platform before that, it was automatically guided towards the goal. At each timestep, the agent moved 6 cm in the direction given by the selected module.

To assess different hypotheses about the mechanisms that govern spatial learning in these tasks, we reproduced all experimental paradigms using three different instantiations (i.e., groups) of the model corresponding to the approaches mentioned before. The first one (associative approach, group D) was composed of one Direction module per cue, proximal or distal, assuming that each individual cue is associated to the reward by means of a TD-learning algorithm, and that each cue could compete against each other, through associative process taking place at the strategy selection level. The second one (cognitive mapping approach, group P) was constituted of one Planning module integrating all proximal and distal cues in a topological map. This map was learned independently from the position of the goal, as described in the Model Description. The third instance (modular approach, group DP) was composed of both Direction and Planning modules. As explained above, this modular approach assumes that these modules and their corresponding separate neural structures have different learning rules (TD-learning for Direction module(s) and graph search for the Planning module), and focus on specific environmental cues (i.e., the Direction module(s) focuse(s) on proximal cue(s), the Planning module on distal cues). Each instantiation (D, P and DP) also included one separate Exploration module.

For each experimental group, the simulations were repeated 50 times. For associative modules, weights between inputs and action cells, as well as weights between inputs and gating units in the gating network, were randomly initialized. The planning graph (PG) was assumed to be built before the experiment, during a random walk in the environment during 1000 timesteps. This procedure was done for each agent.

Group performance was assessed in terms of the averaged escape latencies (i.e., the number of time steps before reaching the goal). Statistical differences were verified using the Wilcoxon test for comparing samples resulting from the same group, whereas the Mann Whitney test was used for comparing different groups. Significant differences were indicated by one (*p* < 0.05), two (*p* < 0.01) or three (*p* < 0.001) stars. The behavior of agents was also analyzed in terms of the selection rate of each module within trials at different regions of the environment (e.g., around the goal or the proximal cue).

## Supporting information

S1 Text Content: Supplementary methods. (PDF)

S1 Table Environment independent model parameter table. (PDF)

S2 Table Environment dependent model parameter table. (PDF)

S1 FigDetailed schema of the model-based spatial planning module of the model.a) The entorhinal cortex (EC) module encodes idiothetic information (grid cells on the left) and visual information, represented as the sum of the encoded landmarks. b) The dentate gyrus (DG) module receives EC’s output and realizes a Hebbian learning process on the weights $W_{ij}^{\left(EC,DG\right)}$ in order to learn place cells. c) Diffusion of the goal signal within the cognitive graph during the planning process: Hippocampal place cell input is weighted by a weight $W_{ij}^{P}$. The weighted sum gives the value associated to position *r^P^*. When the goal has been reached, the activation-diffusion algorithm (red arrows) assigns a goal value *G_i_* to each node, devalued by factor *α*. d)The devalued goal value within the cognitive graph results in the choice by the agent of a direction Φ^*P*^ that maximizes the goal value (chosen trajectory in red). e) Place field illustrated for 10 learned place cells. f) Receptive field of a node of the cognitive graph learned based on the input of hippocampal place cells. g) Example of a cognitive graph learned in the model-based planning module for Experiment III. The grey disk represents the current platform location.(PDF)

S2 Fig Examples of assignments of environmental cues to different modules within the model. a) Cue assignment used for Experiment V. b) Cue assignment used for Experiment VI.
(PDF)

S3 Fig Experiment I. a) Selection rate of the strategies. b) Performance when the model-based Planning strategy in the model is replaced by a model-free Locale strategy. D: Direction Strategy; E: Exploration Strategy; L: Locale Strategy; P: Planning Strategy.
(PDF)

S4 Fig Experiment II. Simulation results with a) the Direction strategy only, b) the Direction and Locale strategies together, c) the full model illustrating the contribution of individual strategies to the behavior of each strategy in terms of % of time where they are selected.
(PDF)

S5 Fig Experiment III. (a-d) Simulation results with the full model (Direction (D) Planning (P) and Exploration (E) strategies together). a) Session-by-session selection rate of strategies. b) Selelection rate of strategies by types of trials. c) Selection rates of strategies by types of simulated animals: Cue Responders (CR) and Place Responders (PR). d) Examples of individual simulated trajectories at the competition trial #10 (adapted from [14]). (e-h) Simulated results when the model-based Planning strategy in the model is replaced by a model-free Locale (L) strategy. e) Session-by-session selection rate of strategies. f) Selection rate of strategies by types of trials. g) Reproduction of the experimental results of [16]. h) Examples of individual simulated trajectories at the competition trial #10.
(PDF)

S6 Fig Experiment IV. (a-c) Simulation results with the full model (Direction (D) Planning (P) and Exploration (E) strategies together). a) Experimental predictions raised when the hippocampus in the model is lesioned (Group D) versus when when the striatum in the model is lesioned (Group P). b) Occupancy rate in the quadrants containing either the previous or the current platform location at the first and fourth trial of each session. c) Selection rate of each strategy in the full model during the first versus the fourth trial of each session. d) Simulated results when the model-based Planning strategy in the model is replaced by a model-free Locale (L) strategy.
(PDF)

S7 Fig Experiment V. (a-b) Selection rate of strategies during Stage 1 and Stage 2 in groups P (left) and D (right). (c-d) Comparison of the occupancy rate during test trials between Octant B, Before reaching Octant B, and After reaching Octant B for groups DP (left) and D (right). Within each octant is also shown the selection rate of strategies that contributed to this occupancy pattern. (e-f) Examples of typical trajectories of groups DP (left) and D (right) for tests 45° and 135°.
(PDF)

S8 Fig Experiment VI. Results with a model only employing the Planning (P) strategy combined with an Exploration strategy. a) The time spent in the quadrant containing the previous platform location is significantly above chance (dashed line) in all conditions, unlike experimental results. b) Escape latencies during Stage 2 do not show an improvement and are significantly different between conditions Trial and Session, unlike experimental results. c) Selection rate of each strategy in each condition of the task underlying the behavior of the P model. d) Details of strategy selection for model P during the test trial of Stage 3, before reaching for the first time the goal quadrant (first column) and after, during the occupancy of the goal quadrant (second column) and of the others (third column).
(PDF)

S9 Fig Experiment VI. Detailed results with the full DP model and the D model. a) Selection rate of each strategy in each condition of the task for the D model. b) Performance of group DP (Trial-Same and Session-Same) during the first trial and the fourth trial of a session during Stage 1. c) Evolution of weights in the gating network between the inputs and their dedicated strategies units for Session-Same and Trial-Same conditions of the DP model. (d-e) Selection rates of strategies in Session-Same, Session-Diff, Trial-Same and Trial-Diff conditions of the DP model (d) and the D model (e) during Stage 1 and Stage 2.
(PDF)

## References

[1] Packard MG, Goodman J. Factors that influence the relative use of multiple memory systems. Hippocampus. 2013;23(11):1044–1052. doi: 10.1002/hipo.22178 23929809 10.1002/hipo.22178

[2] Yin HH, Knowlton BJ. Contributions of striatal subregions to place and response learning. Learn & Mem. 2004;11(4):459–463. doi: 10.1101/lm.81004 10.1101/lm.81004 PMC498333 15286184

[3] Yin HH, Ostlund SB, Knowlton BJ, Balleine BW. The role of the dorsomedial striatum in instrumental conditioning. Eur J Neurosci. 2005;22:513–523. doi: 10.1111/j.1460-9568.2005.04218.x 16045504 10.1111/j.1460-9568.2005.04218.x

[4] Daw ND, Niv Y, Dayan P. Uncertainty-based competition between prefrontal and dorsolateral striatal systems for behavioral control. Nat Neurosci. 2005;8(12):1704–1711. doi: 10.1038/nn1560 16286932 10.1038/nn1560

[5] Yin HH, Knowlton BJ. The role of the basal ganglia in habit formation. Nat Rev Neurosci. 2006;2006:464–76. doi: 10.1038/nrn1919 10.1038/nrn1919 16715055

[6] Thorn CA, Atallah H, Howe M, Graybiel AM. Differential dynamics of activity changes in dorsolateral and dorsomedial striatal loops during learning. Neuron. 2010;66:781–795. doi: 10.1016/j.neuron.2010.04.036 20547134 10.1016/j.neuron.2010.04.036 PMC3108575

[7] Bornstein AM, Daw ND. Multiplicity of control in the basal ganglia: computational roles of striatal subregions. Curr Opin Neurobiol. 2011;21:374–380. doi: 10.1016/j.conb.2011.02.009 21429734 10.1016/j.conb.2011.02.009 PMC3269306

[8] Khamassi M, Humphries MD. Integrating cortico-limbic-basal ganglia architectures for learning model-based and model-free navigation strategies. Front Behav Neurosci. 2012;6 doi: 10.3389/fnbeh.2012.00079 23205006 10.3389/fnbeh.2012.00079 PMC3506961

[9] Chavarriaga R, Strosslin T, Sheynikhovich D, Gerstner W. A Computational Model of Parallel Navigation Systems in Rodents. Neuroinformatics. 2005;3(3):223–242. doi: 10.1385/NI:3:3:223 16077160 10.1385/NI:3:3:223

[10] Girard B, Filliat D, Meyer JA, Berthoz A, Guillot A. Integration of navigation and action selection functionalities in a computational model of cortico-basal-thalamo-cortical loops. Adapt Behav. 2005;13(2):115–130. doi: 10.1177/105971230501300204

[11] Keramati M, Dezfouli A, Piray P. Speed/Accuracy trade-off between the habitual and goal-directed processes. PLoS Comput Biol. 2011;7(5):1–25. doi: 10.1371/journal.pcbi.1002055 10.1371/journal.pcbi.1002055 PMC3102758 21637741

[12] Lesaint F, Sigaud O, Flagel SB, Robinson TE, Khamassi M. Modelling individual differences observed in Pavlovian autoshaping in rats using a dual learning systems approach and factored representations. PLoS Comput Biol. 2014;10(2):e1003466. 24550719 10.1371/journal.pcbi.1003466 PMC3923662

[13] Dollé L, Khamassi M, Girard B, Guillot A, Chavarriaga R. Analyzing Interactions between Navigation Strategies Using a Computational Model of Action Selection In: Spatial Cognition VI. vol. 5248 of LNAI 5248. Springer-Verlag; 2008 p. 71–86.

[14] Dollé L, Sheynikhovich D, Girard B, Chavarriaga R, Guillot A. Path planning versus cue responding: A bioinspired model of switching between navigation strategies. Biol Cybern. 2010;103(4):299–317. doi: 10.1007/s00422-010-0400-z 20617443 10.1007/s00422-010-0400-z

[15] van der Meer M, Kurth-Nelson Z, Redish AD. Information processing in decision-making systems. The Neuroscientist. 2012;18(4):342–359. doi: 10.1177/1073858411435128 22492194 10.1177/1073858411435128 PMC4428660

[16] Devan BD, White NM. Parallel information processing in the dorsal striatum: Relation to hippocampal functions. J Neurosci. 1999;19(7):2789–2798. 10087090 10.1523/JNEUROSCI.19-07-02789.1999 PMC6786063

[17] Foster DJ, Morris RG, Dayan P. A model of hippocampally dependent navigation, using the temporal difference learning rule. Hippocampus. 2000;10(1):1–16. doi: 10.1002/(SICI)1098-1063(2000)10:1%3C1::AID-HIPO1%3E3.0.CO;2-1 10706212 10.1002/(SICI)1098-1063(2000)10:1<1::AID-HIPO1>3.0.CO;2-1

[18] Pezzulo G, Rigoli F, Chersi F. The mixed instrumental controller: using value of information to combine habitual choice and mental simulation. Front Psychol. 2013;4:92 doi: 10.3389/fpsyg.2013.00092 23459512 10.3389/fpsyg.2013.00092 PMC3586710

[19] Viejo G, Khamassi M, Brovelli A, Girard B. Modeling choice and reaction time during arbitrary visuomotor learning through the coordination of adaptive working memory and reinforcement learning. Front Behav Neurosci. 2015;9 doi: 10.3389/fnbeh.2015.00225 26379518 10.3389/fnbeh.2015.00225 PMC4549628

[20] Renaudo E, Girard B, Chatila R, Khamassi M. Design of a Control Architecture for Habit Learning in Robots. In: Biomimetic and Biohybrid Systems, LNAI Proceedings; 2014. p. 249–260.

[21] Collins AG, Frank MJ. How much of reinforcement learning is working memory, not reinforcement learning? A behavioral, computational, and neurogenetic analysis. Eur J Neurosci. 2012;35(7):1024–1035. doi: 10.1111/j.1460-9568.2011.07980.x 22487033 10.1111/j.1460-9568.2011.07980.x PMC3390186

[22] Viejo G, Girard B, Procyk E, Khamassi M. Adaptive coordination of working-memory and reinforcement learning in non-human primates performing a trial-and-error problem solving task. Behav Brain Res. 2017;in press. doi: 10.1016/j.bbr.2017.09.030 29061387 10.1016/j.bbr.2017.09.030

[23] Burgess N, O’Keefe J. Neuronal computations underlying the firing of place cells and their role in navigation. Hippocampus. 1996;6(6):749–762. doi: 10.1002/(SICI)1098-1063(1996)6:6%3C749::AID-HIPO16%3E3.0.CO;2-0 9034860 10.1002/(SICI)1098-1063(1996)6:6<749::AID-HIPO16>3.0.CO;2-0

[24] Arleo A, Gerstner W. Spatial cognition and neuro-mimetic navigation: a model of hippocampal place cell activity. Biol Cybern. 2000;83(3):287–299. doi: 10.1007/s004220000171 11007302 10.1007/s004220000171

[25] Holroyd CB, McClure SM. Hierarchical control over effortful behavior by rodent medial frontal cortex: A computational model. Psychol Rev. 2015;122(1):54 doi: 10.1037/a0038339 25437491 10.1037/a0038339

[26] Gerfen CR, Wilson CJ. The basal ganglia In: Swanson LW, Bjorklund A, Hokfelt T (Eds). Handbook of chemical neuroanatomy. vol. Vol 12: Integrated Systems of the CNS, Part III. Elsevier Science B.V.; 1996 p. 371–468.

[27] Redgrave P, Prescott TJ, Gurney K. The basal ganglia: a vertebrate solution to the selection problem? Neuroscience. 1999;89:1009–1024. 10362291 10.1016/s0306-4522(98)00319-4

[28] Prescott TJ, Redgrave P, Gurney K. Layered control architectures in robots and vertebrates. Adapt Behav. 1999;7:99–127. doi: 10.1177/105971239900700105

[29] Joel D, Niv Y, Ruppin E. Actor-critic models of the basal ganglia: new anatomical and computational perspectives. Neural Netw. 2002;15(4):535–547. doi: 10.1016/S0893-6080(02)00047-3 12371510 10.1016/s0893-6080(02)00047-3

[30] Khamassi M, Lacheze L, Girard B, Berthoz A, Guillot A. Actor-critic models of reinforcement learning in the basal ganglia: from natural to artificial rats. Adapt Behav. 2005;13(2):131–148. doi: 10.1177/105971230501300205

[31] Frank M J and Claus ED. Anatomy of a decision: striato-orbitofrontal interactions in reinforcement learning, decision making, and reversal. Psychol Rev. 2006;113(2):300–26. doi: 10.1037/0033-295X.113.2.300 16637763 10.1037/0033-295X.113.2.300

[32] Stephenson-Jones M, Samuelsson E, Ericsson J, Robertson B, Grillner S. Evolutionary conservation of the basal ganglia as a common vertebrate mechanism for action selection. Current Biol. 2011;21(13):1081–1091. doi: 10.1016/j.cub.2011.05.001 10.1016/j.cub.2011.05.001 21700460

[33] Ujfalussy B, Eros P, Somogyvari Z, Kiss T. Episodes in space: A modelling study of hippocampal place representation. LNAI. 2008;5040:123–136.

[34] Morris R, Garrud P and Rawlins J, O’Keefe J. Place navigation impaired in rats with hippocampal lesions. Nature. 1982;297(5868):681–683. doi: 10.1038/297681a0 7088155 10.1038/297681a0

[35] Steele RJ, Morris RGM. Delay-dependent impairment of a matching-to-place task with chronic and intrahippocampal infusion of the NMDA-antagonist D-AP 5. Hippocampus. 1999;9(2):118–136. doi: 10.1002/(SICI)1098-1063(1999)9:2%3C118::AID-HIPO4%3E3.0.CO;2-8 10226773 10.1002/(SICI)1098-1063(1999)9:2<118::AID-HIPO4>3.0.CO;2-8

[36] Pearce JM, Roberts AD, Good M. Hippocampal lesions disrupt navigation based on cognitive maps but not heading vectors. Nature. 1998;396(6706):75–77. doi: 10.1038/23941 9817202 10.1038/23941

[37] Rodrigo T, Sansa J, Baradad P, Chamizo V. Generalization gradients in a navigation task with rats. Learning and Motivation. 2006;37(3):247–268. doi: 10.1016/j.lmot.2005.08.001

[38] Roberts A, Pearce J. Blocking in the Morris swimming pool. J Exp Psychol Anim Behav Process. 1999;25(2):225–235. doi: 10.1037/0097-7403.25.2.225 10331921 10.1037//0097-7403.25.2.225

[39] Bennett AT. Do animals have cognitive maps? J Exp Biol. 1996;199(1):219–224. 8576693 10.1242/jeb.199.1.219

[40] Sturz BR, Bodily KD, Katz JS, Kelly DM. Evidence against integration of spatial maps in humans: generality across real and virtual environments. Animal Cognition. 2009;12(2):237–247. doi: 10.1007/s10071-008-0182-z 18766392 10.1007/s10071-008-0182-z

[41] Brown VJ, Bowman EM. Rodent models of prefrontal cortical function. Trends Neurosci. 2002;25(7):340–343. doi: 10.1016/S0166-2236(02)02164-1 12079756 10.1016/s0166-2236(02)02164-1

[42] McGregor A, Good MA, Pearce JM. Absence of an Interaction Between Navigational Strategies Based on Local and Distal Landmarks. J Exp Psychol Anim Behav Process. 2004;30(1):34–44. doi: 10.1037/0097-7403.30.1.34 14709113 10.1037/0097-7403.30.1.34

[43] Pearce JM. The 36th Sir Frederick Bartlett Lecture: An associative analysis of spatial learning. Quat J Exp Psychol. 2009;62(1):1665–1684. doi: 10.1080/17470210902805589 10.1080/17470210902805589 19418377

[44] White NM, McDonald RJ. Multiple parallel memory systems in the brain of the rat. Neurobiol Learn Mem. 2002;77:125–184. doi: 10.1006/nlme.2001.4008 11848717 10.1006/nlme.2001.4008

[45] Girardeau G, Benchenane K, Wiener SI, Buzsaki G, Zugaro MB. Selective suppression of hippocampal ripples impairs spatial memory. Nat Neurosci. 2009;12(10):1222–1223. doi: 10.1038/nn.2384 19749750 10.1038/nn.2384

[46] Trullier O, Wiener SI, Berthoz A, Meyer JA. Biologically-based artificial navigation systems: review and prospects. Prog Neurobiol. 1997;83(3):271–285. 10.1016/s0301-0082(96)00060-3 9153072

[47] Redish AD. Beyond the cognitive map: From place cells to episodic memory. The MIT Press; 1999.

[48] Khamassi M. Complementary roles of the rat prefrontal cortex and striatum in reward-based learning and shifting navigation strategies. UPMC. PhD thesis; 2007.

[49] Schmitzer-Torbert NC, Redish AD. Task-dependent encoding of space and events by striatal neurons is dependent on neural subtype. Neurosci. 2008;153:349–360. doi: 10.1016/j.neuroscience.2008.01.081 10.1016/j.neuroscience.2008.01.081 PMC4206194 18406064

[50] Kelly DM, Gibson BM. Spatial navigation: Spatial learning in real and virtual environments. Comp Cog Behav Rev. 2007;2:111–124.

[51] Leising KJ, Blaisdell AP. Associative Basis of Landmark Learning and Integration in Vertebrates. Comp Cog Behav Rev. 2009;4:80–102. 10.3819/ccbr.2009.40010 PMC2895939 20607101

[52] Pavlov I. Conditioned reflexes. London: Oxford University Press; 1927.

[53] Skinner B. The behavior of organisms. New York: Appleton-Century-Crofts; 1938.

[54] Watson JB. Psychology as the Behaviorist views it (1913) Readings in the History of Psychology New York: Appleton-Century-Crofts; 1948.

[55] Rescorla R, Wagner A. A theory of Pavlovian conditioning: The effectiveness of reinforcement and non-reinforcement In: Black AH, Prokasy WF (Eds). Classical conditioning II: Current research and theory. New York: Appleton-Century-Crofts; 1972 p. 64–69.

[56] Tolman EC. Cognitive Maps in Rats and Men. Psychol Rev. 1948;55(4):189–208. doi: 10.1037/h0061626 18870876 10.1037/h0061626

[57] O’Keefe J, Dostrovsky J. The hippocampus as a spatial map. preliminary evidence from unit activity in the freely-moving rat. Brain Res. 1971;34(1):171–175. doi: 10.1016/0006-8993(71)90358-1 5124915 10.1016/0006-8993(71)90358-1

[58] Morris R. Spatial localisation does not require the presence of local cues. Learning and Motivation. 1981;12:239–260. doi: 10.1016/0023-9690(81)90020-5

[59] McDonald RJ, Devan BD, Hong NS. Multiple memory systems: The power of interactions. Neurobiol Learn Mem. 2004;82(3):333–346. doi: 10.1016/j.nlm.2004.05.009 15464414 10.1016/j.nlm.2004.05.009

[60] McDonald RJ, Hong NS, Devan BD. The challenges of understanding mammalian cognition and memory-based behaviours: an interactive learning and memory systems approach. Neurosci Biobehav Rev. 2004;28(7):719–745. doi: 10.1016/j.neubiorev.2004.09.014 15555681 10.1016/j.neubiorev.2004.09.014

[61] White NM. The role of stimulus ambiguity and movement in spatial navigation: a multiple memory systems analysis of location discrimination. Neurobiol Learn Mem. 2004;82:216–229. doi: 10.1016/j.nlm.2004.05.004 15464405 10.1016/j.nlm.2004.05.004

[62] Albertin SV, Mulder AB, Tabuchi E, Zugaro MB, Wiener SI. Lesions of the medial shell of the nucleus accumbens impair rats in finding larger rewards, but spare reward-seeking behavior. Behav Brain Res. 2000;117(1-2):173–83. doi: 10.1016/S0166-4328(00)00303-X 11099771 10.1016/s0166-4328(00)00303-x

[63] Grahn JA, Parkinson JA, Owen AM. The cognitive functions of the caudate nucleus. Prog Neurobiol. 2008;86(3):141–155. doi: 10.1016/j.pneurobio.2008.09.004 18824075 10.1016/j.pneurobio.2008.09.004

[64] Granon S, Poucet B. Medial prefrontal lesions in the rat and spatial navigation: Evidence for impaired planning. Behav Neurosci. 1995;109(3):474–484. doi: 10.1037/0735-7044.109.3.474 7662158 10.1037//0735-7044.109.3.474

[65] Jankowski J, Scheef L, Huppe C, Boecker H. Distinct striatal regions for planning and executing novel and automated movement sequences. Neuroimage. 2009;44(4):1369–1379. doi: 10.1016/j.neuroimage.2008.10.059 19059350 10.1016/j.neuroimage.2008.10.059

[66] Packard MG, McGaugh JL. Inactivation of hippocampus or caudate nucleus with lidocaine differentially affects expression of place and response learning. Neurobiol Learn Mem. 1996;65(1):65–72. doi: 10.1006/nlme.1996.0007 8673408 10.1006/nlme.1996.0007

[67] White NM. Some highlights of research on the effects of caudate nucleus lesions over the past 200 years. Behav Brain Res. 2009;199(1):3–23. doi: 10.1016/j.bbr.2008.12.003 19111791 10.1016/j.bbr.2008.12.003

[68] McDonald R, White N. Parallel information processing in the water maze: evidence for independent memory systems involving dorsal striatum and hippocampus. Behav Neural Biol. 1994;61(3):260–70. doi: 10.1016/S0163-1047(05)80009-3 8067981 10.1016/s0163-1047(05)80009-3

[69] Packard MG, McGaugh JL. Double dissociation of fornix and caudate nucleus lesions on acquisition of two water maze tasks: further evidence for multiple memory systems. Behav Neurosci. 1992;106(3):439–446. doi: 10.1037/0735-7044.106.3.439 1616610 10.1037//0735-7044.106.3.439

[70] Clark RE, Broadbent NJ, Squire LR. The Hippocampus and Spatial Memory: Findings with a Novel Modification of the Water Maze. J Neurosci. 2007;27(25):6647–6654. doi: 10.1523/JNEUROSCI.0913-07.2007 17581951 10.1523/JNEUROSCI.0913-07.2007 PMC2553679

[71] Hamilton DA, Akers KG, Johnson TE, Rice JP, Candelaria FT, Redhead ES. Evidence for a shift from place navigation to directional responding in one variant of the Morris water task. J Exp Psychol Anim Behav Process. 2009;35(2):271–278. doi: 10.1037/a0013260 19364235 10.1037/a0013260

[72] Gold PE. Coordination of multiple memory systems. Neurobiol Learn Mem. 2004;82(3):230–242. doi: 10.1016/j.nlm.2004.07.003 15464406 10.1016/j.nlm.2004.07.003

[73] Hartley T, Burgess N. Complementary memory systems: Competition, cooperation and compensation. Trends Neurosci. 2005;28(4):169–170. doi: 10.1016/j.tins.2005.02.004 15808348 10.1016/j.tins.2005.02.004

[74] Kim J, Baxter M. Multiple brain-memory systems: The whole does not equal the sum of its parts. Trends Neurosci. 2001;24(6):324–30. doi: 10.1016/S0166-2236(00)01818-X 11356503 10.1016/s0166-2236(00)01818-x

[75] Poldrack RA, Packard MG. Competition among multiple memory systems: Converging evidence from animal and human brain studies. Neuropsychology. 2003;41(3):245–51. doi: 10.1016/S0028-3932(02)00157-4 10.1016/s0028-3932(02)00157-4 12457750

[76] Chang Q, Gold PE. Switching memory systems during learning: Changes in patterns of brain acetylcholine release in the hippocampus and striatum in rats. J Neurosci. 2003;23(7):3001–3005. 12684487 10.1523/JNEUROSCI.23-07-03001.2003 PMC6742106

[77] Voermans NC, Petersson KM, Daudey L, Weber B, Van Spaendonck KP, Kremer HPH, et al Interaction between the human hippocampus and the caudate nucleus during route recognition. Neuron. 2004;43(3):427–435. doi: 10.1016/j.neuron.2004.07.009 15294149 10.1016/j.neuron.2004.07.009

[78] Pych JC, Chang Q, Colon-Rivera C, Haag R, Gold PE. Acetylcholine release in the hippocampus and striatum during place and response training. Learn & Mem. 2005;12(6):564–572. doi: 10.1101/lm.33105 10.1101/lm.33105 PMC1356173 16322358

[79] Ragozzino ME, Detrick S, Kesner RP. Involvement of the Prelimbic-Infralimbic Areas of the Rodent Prefontal Cortex in Behavioral Flexibility for Place and Response Learning. J Neurosci. 1999;19(11):4585–4594. 10341256 10.1523/JNEUROSCI.19-11-04585.1999 PMC6782617

[80] Atallah HE, Lopez-Paniagua D, Rudy JW, O’Reilly RC. Separate neural substrates for skill learning and performance in the ventral and dorsal striatum. Nat Neurosci. 2007;10:126–131. doi: 10.1038/nn1817 17187065 10.1038/nn1817

[81] Humphries MD, Prescott TJ. The ventral basal ganglia, a selection mechanism at the crossroads of space, strategy, and reward. Prog Neurobiol. 2010;90:385–417. doi: 10.1016/j.pneurobio.2009.11.003 19941931 10.1016/j.pneurobio.2009.11.003

[82] van der Meer MAA, Redish AD. Ventral striatum: a critical look at models o learning and evaluation. Curr Opin Neurobiol. 2011;21(3):387–392. doi: 10.1016/j.conb.2011.02.011 21420853 10.1016/j.conb.2011.02.011 PMC3134536

[83] Martinet LE, Sheynikhovich D, Benchenane K, Arleo A. Spatial Learning and Action Planning in a Prefrontal Cortical Network Model. PLoS Comput Biol. 2011;7(5). doi: 10.1371/journal.pcbi.1002045 21625569 10.1371/journal.pcbi.1002045 PMC3098199

[84] Caluwaerts K, Staffa M, N’Guyen S, Grand C, Dollé L, Favre-Félix A, et al A biologically inspired meta-control navigation system for the Psikharpax rat robot. Bioinsp & Biomim. 2012;7(2):025009. 10.1088/1748-3182/7/2/025009 22617382

[85] Kubie JL, Fenton AA. Heading-vector navigation based on head-direction cells and path integration. Hippocampus. 2009;19(5):456–479. doi: 10.1002/hipo.20532 19072761 10.1002/hipo.20532

[86] Poucet B. Spatial cognitive maps in animals: New hypotheses on their structure and neural mechanisms. Psychol Rev. 1993;100(2):163–182. doi: 10.1037/0033-295X.100.2.163 8483980 10.1037/0033-295x.100.2.163

[87] Skinner DM, Etchegary CM, Ekert-Maret EC, Baker CJ, Harley CW, Evans JH, et al An Analysis of Response, Direction, and Place Learning in an Open Field and T Mazes. J Exp Psychol Anim Behav Process. 2003;29:3–13. doi: 10.1037/0097-7403.29.1.3 12561129

[88] Gibson BM. Cognitive maps not used by humans (Homo sapiens) during a dynamic navigational task. J Comp Psychol. 2001;115(4):397 doi: 10.1037/0735-7036.115.4.397 11824903

[89] Doeller CF, King JA, Burgess N. Parallel striatal and hippocampal systems for landmarks and boundaries in spatial memory. Proc Natl Acad Sci USA. 2008;105(15):5915–5920. doi: 10.1073/pnas.0801489105 18408152 10.1073/pnas.0801489105 PMC2311337

[90] Doeller CF, Burgess N. Distinct error-correcting and incidental learning of location relative to landmarks and boundaries. Proc Natl Acad Sci USA. 2008;105(15):5909–5914. doi: 10.1073/pnas.0711433105 18413609 10.1073/pnas.0711433105 PMC2311326

[91] Hamilton DA, Akers KG, Johnson TE, Rice JP, Candelaria FT, Sutherland RJ, et al The relative influence of place and direction in the Morris water task. J Exp Psychol Anim Behav Process. 2008;34(1):31–53. doi: 10.1037/0097-7403.34.1.31 18248113 10.1037/0097-7403.34.1.31

[92] Knierim JJ, Rao G. Distal landmarks and hippocampal place cells: effects of relative translation versus rotation. Hippocampus. 2003;13(5):604–617. doi: 10.1002/hipo.10092 12921350 10.1002/hipo.10092

[93] Glascher J, Daw N, Dayan P, O’Doherty JP. States versus rewards: dissociable neural prediction error signals underlying model-based and model-free reinforcement learning. Neuron. 2010;66(4):585–595. doi: 10.1016/j.neuron.2010.04.016 20510862 10.1016/j.neuron.2010.04.016 PMC2895323

[94] Eichenbaum H. Prefrontal-hippocampal interactions in episodic memory. Nat Rev Neurosci. 2017;18(9):547 doi: 10.1038/nrn.2017.74 28655882 10.1038/nrn.2017.74

[95] Constantinescu AO, O’Reilly JX, Behrens TE. Organizing conceptual knowledge in humans with a grid-like code. Science. 2016;352(6292):1464–1468. doi: 10.1126/science.aaf0941 27313047 10.1126/science.aaf0941 PMC5248972

[96] Tavares RM, Mendelsohn A, Grossman Y, Williams CH, Shapiro M, Trope Y, et al A map for social navigation in the human brain. Neuron. 2015;87(1):231–243. doi: 10.1016/j.neuron.2015.06.011 26139376 10.1016/j.neuron.2015.06.011 PMC4662863

[97] Shohamy D, Daw ND. Integrating memories to guide decisions. Curr Opin Behav Sci. 2015;5:85–90. doi: 10.1016/j.cobeha.2015.08.010

[98] Foerde K, Shohamy D. Feedback timing modulates brain systems for learning in humans. J Neurosci. 2011;31(37):13157–13167. doi: 10.1523/JNEUROSCI.2701-11.2011 21917799 10.1523/JNEUROSCI.2701-11.2011 PMC3328791

[99] Foerde K, Shohamy D. The role of the basal ganglia in learning and memory: insight from Parkinson’s disease. Neurobiol Learn & Mem. 2011;96(4):624–636. doi: 10.1016/j.nlm.2011.08.006 10.1016/j.nlm.2011.08.006 PMC3772079 21945835

[100] Schuck NW, Cai MB, Wilson RC, Niv Y. Human orbitofrontal cortex represents a cognitive map of state space. Neuron. 2016;91(6):1402–1412. doi: 10.1016/j.neuron.2016.08.019 27657452 10.1016/j.neuron.2016.08.019 PMC5044873

[101] Benchenane K, Peyrache A, Khamassi M, Tierney PL, Gioanni Y, Battaglia FP, et al Coherent theta oscillations and reorganization of spike timing in the hippocampal-prefrontal network upon learning. Neuron. 2010;66(6):921–936. doi: 10.1016/j.neuron.2010.05.013 20620877 10.1016/j.neuron.2010.05.013

[102] Schultz W, Stauffer WR, Lak A. The phasic dopamine signal maturing: from reward via behavioural activation to formal economic utility. Curr Opin Neurobiol. 2017;43:139–148. doi: 10.1016/j.conb.2017.03.013 28390863 10.1016/j.conb.2017.03.013

[103] Shohamy D, Wagner AD. Integrating memories in the human brain: hippocampal-midbrain encoding of overlapping events. Neuron. 2008;60(2):378–389. doi: 10.1016/j.neuron.2008.09.023 18957228 10.1016/j.neuron.2008.09.023 PMC2628634

[104] Sutton RS, Barto AG. Reinforcement learning: an introduction. The MIT Press, Bradford Book; 1998.

[105] Cazé R, Khamassi M, Aubin L, Girard B. Hippocampal replays under the scrutiny of reinforcement learning models. Submitted. 2018. 10.1152/jn.00145.2018 30303758

[106] Gomperts SN, Kloosterman F, Wilson MA. VTA neurons coordinate with the hippocampal reactivation of spatial experience. Elife. 2015;4:e05360 doi: 10.7554/eLife.05360 26465113 10.7554/eLife.05360 PMC4695386

[107] Khamassi M, Martinet LE, Guillot A. Combining self-organizing maps with mixture of experts: application to an actor-critic of reinforcement learning in the basal ganglia. In: Proceedings of the 9th International Conference on the Simulation of Adaptive Behavior (SAB 2006); 2006. p. 394–405.

[108] Doya K, Samejima K, Katagiri, Kawato M. Multiple model-based reinforcement learning. Neural Computation. 2002;14(6):1347–1369. doi: 10.1162/089976602753712972 12020450 10.1162/089976602753712972

[109] Luksys G, Gerstner W, Sandi C. Stress, genotype and norepinephrine in the prediction of mouse behavior using reinforcement learning. Nat Neurosci. 2009;12(9):1180–1186. doi: 10.1038/nn.2374 19684590 10.1038/nn.2374

[110] Behrens TE, Woolrich MW, Walton ME, Rushworth MF. Learning the value of information in an uncertain world. Nature neuroscience. 2007;10(9):1214 doi: 10.1038/nn1954 17676057 10.1038/nn1954

[111] Morris G, Nevet A, Arkadir D, Vaadia E, Bergman H. Midbrain dopamine neurons encode decisions for future action. Nat Neurosci. 2006;9(8):1057–1063. doi: 10.1038/nn1743 16862149 10.1038/nn1743

[112] Roesch MR, Calu DJ, Schoenbaum G. Dopamine neurons encode the better option in rats deciding between differently delayed or sized rewards. Nat Neurosci. 2007;10(12):1615–1624. doi: 10.1038/nn2013 18026098 10.1038/nn2013 PMC2562672

[113] Niv Y, Daw ND, Dayan P. Choice values. Nature neuroscience. 2006;9(8):987–988. doi: 10.1038/nn0806-987 16871163 10.1038/nn0806-987

[114] Daw ND. Dopamine: at the intersection of reward and action. Nat Neurosci. 2007;10(12):1505–1507. doi: 10.1038/nn1207-1505 18043583 10.1038/nn1207-1505

[115] Bellot J, Sigaud O, Khamassi M. Which Temporal Difference Learning algorithm best reproduces dopamine activity in a multi-choice task? In: Ziemke T, Balkenius C, Hallam J (Eds). Proceedings of the 12th International Conference on Adaptive Behaviour (SAB 2012). Odense, Denmark: Springer; 2012. p. 289–298.

[116] Bellot J, Sigaud O, Roesch MR, Schoenbaum G, Girard B, Khamassi M. Dopamine neurons phasic activity does not encode the reward prediction error that behavioral adaptation would predict. Submitted. 2018.

[117] Mouret JB. Micro-Data Learning: The Other End of the Spectrum. arXiv preprint arXiv:161000946. 2016.

[118] Taube JS, Muller RU, Ranck JB Jr. Head-direction cells recorded from the postsubiculum in freely moving rats. I. Description and quantitative analysis. J Neurosci. 1990;10(2):420–435. 2303851 10.1523/JNEUROSCI.10-02-00420.1990 PMC6570151

[119] Dollé L, Sheynikhovich D, Girard B, Ujfallussy B, Chavarriaga R, Guillot A. Analyzing interactions between cue-guided and place-based navigation with a computational model of action selection: Influence of sensory cues and training. In: Proceedings of the 11th International Conference on Simulation of Adaptive Behavior (SAB 2010). Paris, France: Springer; 2010. p. 335–346.

[120] Dijkstra EW. A note on two problems in connection with graphs. Numerische Mathematik. 1959;1(269-270):269–271. doi: 10.1007/BF01386390

[121] Guazzelli A, Corbacho FJ, Bota M, Arbib MA. Affordances, motivation, and the world graph theory. Adapt Behav. 1998;6(3):435–471. doi: 10.1177/105971239800600305

[122] D’Hooge R, De Deyn PP. Applications of the Morris water maze in the study of learning and memory. Brain Res Rev. 2001;36(1):60–90. doi: 10.1016/S0165-0173(01)00067-4 11516773 10.1016/s0165-0173(01)00067-4

[123] Chakravarthy V, Joseph D, Bapi RS. What do the basal ganglia do? A modeling perspective. Biol Cybern. 2010;103(3):237–253. doi: 10.1007/s00422-010-0401-y 20644953 10.1007/s00422-010-0401-y

[124] Strosslin T, Sheynikhovich D, Chavarriaga R, Gerstner W. Robust self-localisation and navigation based on hippocampal place cells. Neural Network. 2005;18(9):1125–1140. doi: 10.1016/j.neunet.2005.08.012 10.1016/j.neunet.2005.08.012 16263241

