# Supplementary material for: Interactions of spatial strategies producing generalization gradient and blocking: A computational approach
Source: PLoS Comput Biol. 2018 Apr 9;14(4):e1006092. doi: 10.1371/journal.pcbi.1006092 (PMC5908205; doi:10.1371/journal.pcbi.1006092)
Supplement: S1 Text — (PDF) [file pcbi.1006092.s001.pdf]

S1 Text

March 24, 2018

Supporting information for Dollé et al. (2018) PLoS Computational Biology "Interactions of spatial strategies producing generalization gradient and blocking: A computational approach"

1 Supplementary methods

1.1 Associative model-free *Direction* strategy

1.1.1 Visual inputs

We do not seek here to precisely simulate the perception of animals, but instead to obtain a numeric input that represents the perception of a cue with regards to its distance and direction to the simulated agent.

A visual stimulus is encoded by a vector of N_{CC} cue cells (CC), coding for a panoramic view of the environment in grey levels. The activity of a cell i signals the level of perception of a single cue in the direction Φ^i of the simulated agent:

$$r_i^{CC} = \exp\left(-\frac{(\Phi^l - \Phi^i)^2}{2\sigma_{CC}^2}\right), \quad (1)$$

The width of the Gaussian σ_{CC}, centered on the direction of the target cue, increases when the simulated agent is getting closer, thus simulating the size of the cue in the visual field of the simulated agent. The value of σ_{CC} is obtained by the full width at half maximum of the gaussian, equal to the diameter D_r of the projection of the cue on the retina:

$$\sigma_{CC} = \frac{D_r}{2\sqrt{2log2}} \quad (2)$$

with:

$$D_r = \frac{D_a}{\Delta_{R \to L}} R_r, \quad (3)$$

where D_a is the actual diameter of the cue, R_r the radius of the retina (fixed at 0.5cm) and $\Delta_{R \to L}$ the distance of the simulated agent to the cue.

For example, with $N_{CC} = 100$, a 50cm diameter cue will be perceived up to a distance of 7.50m. The diameter of its projection on the retina will be 0.3cm, corresponding to the the width of activation of a single Cue Cell. In order to simulate the sudden absence of a cue, a uniform noise is then injected to the dedicated CC vector, with a mean equal to 0.1.

1.1.2 Reinforcement learning

Motor responses consist of $N_{AC}^T = 36$ action cells (AC). Each AC receives inputs from all CCs and codes for a movement direction $\phi_i^{Dir} = \frac{2\pi i}{N_{AC}^{Dir}}$.

AC's activity is coding the value of a movement direction an is computed as follows:

$$a_i^{Dir}(t) = \sum_{j=1}^{N_{CC}} r_j^{CC}(t) w_{ij}^{Dir}(t). \quad (4)$$

$a_i^T(t)$ being the Q-value of the corresponding state-action couple. The total activity of the population codes for the direction Φ^T for the next movement of the simulated agent [11]:

$$\Phi^{Dir}(t) = \arctan\left(\frac{\sum_i a_i^{Dir}(t)\sin(\phi_i^{Dir})}{\sum_i a_i^{Dir}(t)\cos(\phi_i^{Dir})}\right). \tag{5}$$

Q-value $A^{Dir}(\Phi^{Dir}(t))$ is defined as the intersection of both a_i^{Dir} coding for the closest directions from $\Phi^T(t)$ Weights w_{ij}^{Dir} are updated following the Q-learning rule [12]:

$$\Delta w_{ij}^{Dir} = \eta^{Dir}\delta^{Dir}(t)e_{ij}^{Dir}(t+1). \tag{6}$$

Where η^{Dir} is the learning rate and $\delta^{Dir}(t)$ the reward prediction error.

$$\delta^{Dir}(t) = R(t+1) + \gamma^{Dir}A^{Dir}(\Phi^{Dir}(t+1)) - A^{Dir}(\Phi^{Dir}(t)), \tag{7}$$

where $R(t+1)$ is the reward delivered at time $t+1$, $0 < \gamma < 1$ is the decay factor and A^T, the Q-value of the action executed at time t, as estimated by the strategy.

The reward prediction error and the updating of the corresponding weights are also computed when the action at time t has been generated but the strategy's policy (i.e., following the action of another strategies, or a random move), allowing the learning process to continue.

Eligibility traces e_{ij}^T speed up learning by memorizing state-action couples:

$$e_{ij}^{Dir}(t+1) = r_j^{CC}(t)r_i^{AC}(t) + \lambda^{Dir}e_{ij}^{Dir}(t), \tag{8}$$

where λ^{Dir} is the trace decay factor and r_i^{AC} is computed as follows:

$$r_i^{AC}(t) = \exp\left(\frac{-(\phi_i^{Dir} - \Phi^{Dir}(t))}{(2\sigma^{Dir})^2}\right), \tag{9}$$

This term allows, not only the chosen action, but all the actions to be updated according to their proximity with the chosen action. Along with the equation 5, they allow the algorithm to be performed in the continuous state-action space. This could be implemented in a biologically plausible way by dynamical recurrent networks [14, 4], not modeled here for the sake of simplicity.

1.1.3 Allocentric and egocentric bearing

Two sub strategies can be modeled, depending on the chosen reference frame: with an egocentric reference frame, the direction Φ^l of the perceived landmark is computed relative to the current orientation of the simulated agent. With an allocentric reference frame, the direction is computed relative to an absolute axis $\overrightarrow{x}$, independently from the current orientation of the simulated agent. Motor outputs are then computed in the same reference frame than the inputs. An egocentric Taxon strategy will be termed Guidance, as it is commonly named in the literature, while an allocentric one will be termed Direction, as mentioned by, e.g., [10]. Here, as in [3, 7, 8], all simulations are done with the allocentric Direction strategy, except otherwise stated when an alternative is tested.

1.2 Cognitive model-based *Planning* strategy

1.2.1 Place inputs

Inputs are modeled by a place cells map. They are learned by a hippocampal model originally developed by [13] and consists of two different neural networks, simulating the enthorinal cortex (EC) and the dentate gyrus (DG) (**Suppl. Fig. S1**). This represents a novelty compared to the previous version of our model presented in [8]. EC grid cells and sensorial cells are then fed to the DG, which produces Place Cells by means of a Hebbian learning. Then a sparse representation is computed with a filter function that only keeps a few cells active and sets the others to zero. Detailed computations can be found in [13]. The final activation is then normalized and processed by the Planning strategy to build the nodes of the planning graph (PG).

1.2.2 Graph inputs

The planning graph consists of n^{PG} nodes. All PG nodes are connected to DG cells. The firing rate of a PG node j is computed as follows:

$$r_j^P = f_j(\sum_i W_{ij}^{(DG,PG)} DG_i \ , \ s^{PG}), \tag{10}$$

where W_{ij} is the synaptic weight linking the DG place cell i to the PG node j and $f_j(x, s^{PG})$ is the same non-linear function as in the DG, returning a sparse encoding of x, with a sparseness level of s^{PG}.

The synaptic weights are learned following a Hebbian rule, similar to the one used for learning the DG output:

$$\Delta W_{i,j}^{(DG,PG)} = \alpha^{(DG,PG)} r_j^P (DG_i - W_{i,j}^{(DG,PG)}) \tag{11}$$

A link between nodes N_i and N_j stores the allocentric direction of movement required to move from one node to the other.

1.2.3 Planning learning

The *goal planning phase* begins when the goal position is found, the closest node being set to the delivered reward value. Then, given the PG, the optimal path to the goal is determined by the bio-inspired activation-diffusion mechanism [1] based on Dijkstras algorithm for finding the shortest path between two nodes in a graph [6]. At each timestep, the Planning strategy proposes the corresponding direction. If the goal position is not known, a random direction is proposed.

1.3 Random *Exploration* strategy

Contrary to other biomimetic navigational models using learning algorithms (e.g., [3, 9]), we use an explicit module providing random directional actions (corresponding to an exploratory behavior in the sense of reinforcement learning). The first advantage of having an exploratory strategy is that a single mechanism provides exploratory actions on the basis of which all strategies are updated as if they had selected the executed action. A second advantage, which is a corollary of the first one, is that exploration can be the result of any type of decision mechanism: either purely random decisions, as adopted here for simplicity and parsimony; or more complex exploratory movements leading the simulated agent along the walls (thigmotaxis) or near a new object of interest in the environment [5]. Indeed it should be noticed that randomness is not sufficient to describe exploratory behaviors in real animals. Nevertheless, such an independent exploration strategy is biologically plausible, the existence of such specific module being supported in a recent review [2].

1.4 Associative strategy selection module: The *Gating network*

Each strategy computes at the same time its own proposition of movement. The time spent by the Planning strategy to compute the path is not taken into account as a potential cost in our selection mechanism. The gating network selects at each timestep which of the Direction, Planning or Exploration strategies (D, P and E) will control the future movement, on the basis of candidate directions Φ^k of movement. It consists of three units $k \in \{D; P; E\}$, each corresponding to a strategy. The activity g^k of strategy k is its "gating value". They are computed as the weighted sum of both SC and PG inputs by synaptic weights z^k:

$$g^k(t) = \sum_{j=1}^{N_{SC}} z_j^k(t) r_j^{SC}(t) + \sum_{j=N_{SC}+1}^{N_{SC}+N_{PG}} z_j^k(t) r_j^{PG}(t), \tag{12}$$

where z_j^k is the connection weight between the unit k of the gating network and input unit j of the strategies. A winner-take-all scheme then chooses the next movement direction $\phi^{k'}$:

$$\phi^{k'}(t); k' = \text{argmax}_i(g^i(t)) \tag{13}$$

The gating values connection weights are adjusted using the same Q-learning algorithm as the Direction strategy, except that the update is modulated by the angular difference between the proposed orientation and the one actually chosen, so that the closer an orientation is from the chosen one, the stronger is its update.

1.5 Alternative associative model-free *Locale* strategy

1.5.1 Place inputs

Inputs are modeled by the same place cells map than the Planning strategy. They are composed of dentate gyrus (DG) Place Cells learned through Hebbian learning.

1.5.2 Reinforcement learning

Once DG cells are learned, rule of reinforcement learning are applied to the Locale strategy, the same way there were applied to the Direction strategy: Motor responses consist of $N_{AC}^L = 36$ action cells (AC). Each AC receives inputs from all CCs and codes for a movement direction $\phi_i^L = \frac{2\pi i}{N_{AC}^L}$. equations 4 to 9 are computed, with DG inputs r^{DG} instead of CC inputs r^{CC} in order to respectively compute the values $a_i^L(t)$, $\Phi^L(t)$, Δw_{ij}^L, $\delta^L(t)$, $e_{ij}^L(t+1)$ and r_i^{AC}.

References

[1] Y. Burnod, *Organizational levels of the cerebral cortex: An integrated model.*, Acta Biotheoretica **39** (1991), no. 3-4, 351–361.

[2] VS Chakravarthy, D. Joseph, and R.S. Bapi, *What do the basal ganglia do? A modeling perspective*, Biological Cybernetics **103** (2010), no. 3, 237–253.

[3] Ricardo Chavarriaga, Thomas Strösslin, Denis Sheynikhovich, and Wulfram Gerstner, *A computational model of parallel navigation systems in rodents*, Neuroinformatics **3** (2005), no. 3, 223–242.

[4] S. Denève, P.E. Latham, and A. Pouget, *Reading population codes: a neural implementation of ideal observers*, Nat Neurosci **2** (1999), no. 8, 740–745.

[5] R. D'Hooge and P.P. De Deyn, *Applications of the morris water maze in the study of learning and memory*, Brain Research Reviews **36** (2001), no. 1, 60–90.

[6] E.W. Dijkstra, *A note on two problems in connection with graphs*, Numerische Mathematik **1** (1959), no. 269-270, 269–271.

[7] L. Dollé, M. Khamassi, B. Girard, A. Guillot, and R. Chavarriaga, *Analyzing interactions between navigation strategies using a computational model of action selection*, Spatial Cognition VI, LNAI 5248, vol. 5248, Springer-Verlag, 2008, pp. 71–86.

[8] L. Dollé, D. Sheynikhovich, B. Girard, R. Chavarriaga, and A. Guillot, *Path planning versus cue responding: A bioinspired model of switching between navigation strategies.*, Biological Cybernetics **103** (2010), no. 4, 299–317.

[9] A. Guazzelli, F.J. Corbacho, M. Bota, and M.A. Arbib, *Affordances, motivation, and the world graph theory*, Adaptive Behavior **6** (1998), no. 3, 435–471.

[10] D.A. Hamilton, K.G. Akers, T.E. Johnson, J.P. Rice, F.T. Candelaria, R.J. Sutherland, M.P. Weisend, and E.S. Redhead, *The relative influence of place and direction in the morris water task*, Journal of Experimental Psychology : Animal Behavior Processes **34** (2008), no. 1, 31–53.

[11] T. Strösslin, D. Sheynikhovich, R. Chavarriaga, and W. Gerstner, *Robust self-localisation and navigation based on hippocampal place cells*, Neural Network **18** (2005), no. 9, 1125–1140.

[12] R.S. Sutton and A.G. Barto, *Reinforcement learning: an introduction*, The MIT Press, Bradford Book, 1998.

[13] B. Ujfalussy, P. Eros, Z. Somogyvari, and T. Kiss, *Episodes in space: A modelling study of hippocampal place representation*, LNAI **5040** (2008), 123–136.

[14] H.R. Wilson and J.D. Cowan, *Excitatory and inhibitory interactions in localized populations of model neurons*, Biophys J **12** (1972), no. 1, 1–24.
