# Supplementary material for: Interactions of spatial strategies producing generalization gradient and blocking: A computational approach
Source: PLoS Comput Biol. 2018 Apr 9;14(4):e1006092. doi: 10.1371/journal.pcbi.1006092 (PMC5908205; doi:10.1371/journal.pcbi.1006092)
Supplement: S1 Table — (PDF) [file pcbi.1006092.s002.pdf]

Suppl. Table S1: Model parameters independent from the experiment

Name		Value	Description
**Direction strategy**			
N_{CC}	1	100	Number of cue cells for each landmark
N_{AC}^{Dir}	1	36	Number of action cells for the Direction strategy
σ^{Dir}	1	22.5 $^\circ$	Standard deviation of the generalization profile
λ^{Dir}	1	0.95	Decay factor of the eligibility trace
γ^{Dir}	1	0.8	Decay factor of the future reward
**Locale Strategy**			
λ^{L}	1	0.95	Decay factor of the eligibility trace
γ^{L}	1	0.8	Decay factor of the future reward
η^{L}	4	0.01	Learning rate
**Planning Strategy**			
α	2	0.7	Decay factor of the reward value
N_{PC}	3	1000	Number of place cells
**Gating Network**			
γ_{G}	1	0.8	Decay factor of the future rewarde
λ_{G}	1	0.7	Decay factor of the eligibility trace

[1] same parameter value as in [1];
[2] same parameter value as in [2];
[3] tuned in order to give a sufficient detailed representation to the strategies;
[4] hand-tuned.

References

[1] Ricardo Chavarriaga, Thomas Strösslin, Denis Sheynikhovich, and Wulfram Gerstner, *A computational model of parallel navigation systems in rodents*, Neuroinformatics **3** (2005), no. 3, 223–242.

[2] L. Dollé, D. Sheynikhovich, B. Girard, R. Chavarriaga, and A. Guillot, *Path planning versus cue responding: A bioinspired model of switching between navigation strategies.*, Biological Cybernetics **103** (2010), no. 4, 299–317.
