# Supplementary material for: Interactions of spatial strategies producing generalization gradient and blocking: A computational approach
Source: PLoS Comput Biol. 2018 Apr 9;14(4):e1006092. doi: 10.1371/journal.pcbi.1006092 (PMC5908205; doi:10.1371/journal.pcbi.1006092)
Supplement: S2 Table — (PDF) [file pcbi.1006092.s003.pdf]

Suppl. Table S2: Model parameters dependent from the experiment

**Exp.**	η^T: Learning rate of the Direction strategy	ξ: learning rate of the Gating Network
Exp. I	0.01	0.1
Exp. III	0.25	0.25
Exp. III	0.25	1
Exp. IV	0.1	0.25
Exp. V	0.25	0.25
Exp. VI	0.1	0.1
