# Supplementary figures and images for: Interactions of spatial strategies producing generalization gradient and blocking: A computational approach
Source: PLoS Comput Biol. 2018 Apr 9;14(4):e1006092. doi: 10.1371/journal.pcbi.1006092 (PMC5908205; doi:10.1371/journal.pcbi.1006092)

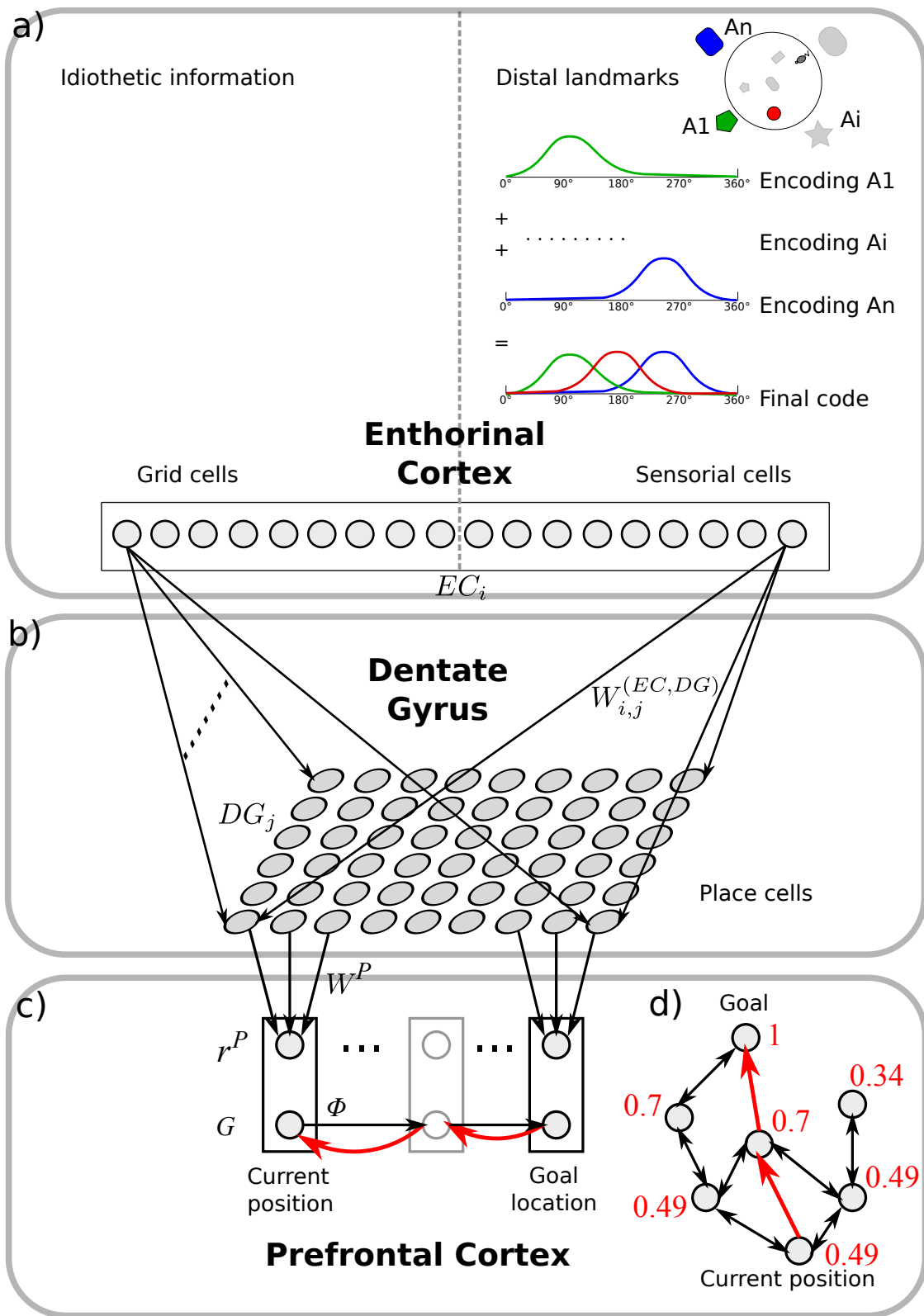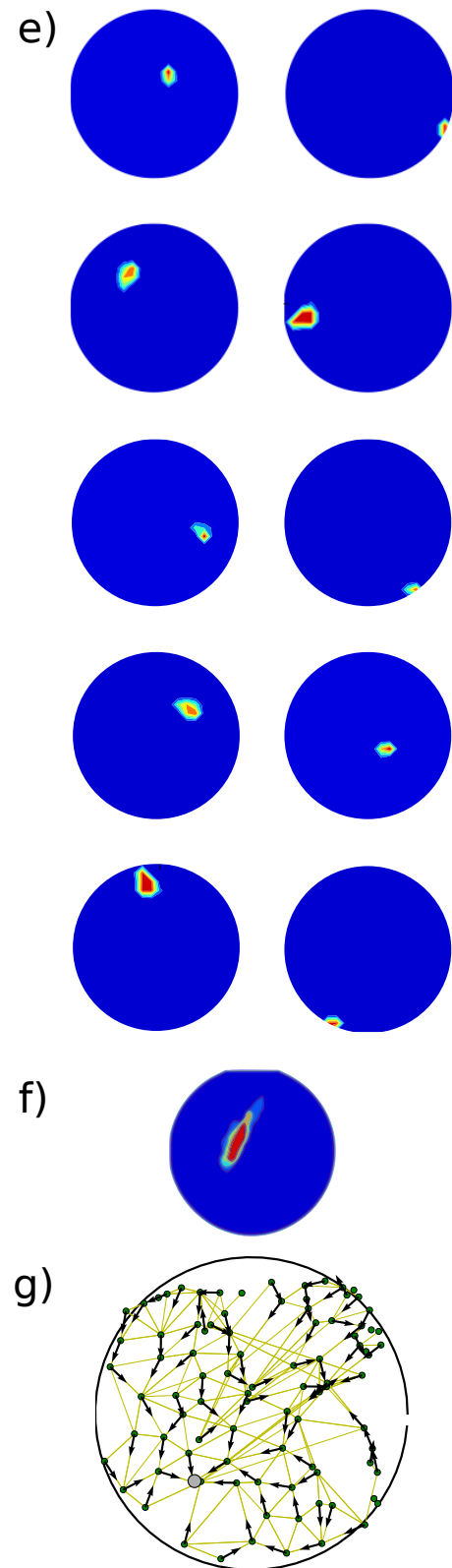

Supplement: S1 Fig — a) The entorhinal cortex (EC) module encodes idiothetic information (grid cells on the left) and visual information, represented as the sum of the encoded landmarks. b) The dentate gyrus (DG) module receives EC’s output and realizes a Hebbian learning process on the weights Wij(EC,DG) in order to learn place cells. c) Diffusion of the goal signal within the cognitive graph during the planning process: Hippocampal place cell input is weighted by a weight WijP. The weighted sum gives the value associated to position rP. When the goal has been reached, the activation-diffusion algorithm (red arrows) assigns a goal value Gi to each node, devalued by factor α. d)The devalued goal value within the cognitive graph results in the choice by the agent of a direction ΦP that maximizes the goal value (chosen trajectory in red). e) Place field illustrated for 10 learned place cells. f) Receptive field of a node of the cognitive graph learned based on the input of hippocampal place cells. g) Example of a cognitive graph learned in the model-based planning module for Experiment III. The grey disk represents the current platform location. (PDF) [file pcbi.1006092.s004.pdf]

a)

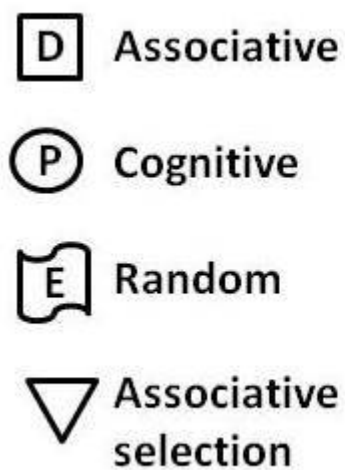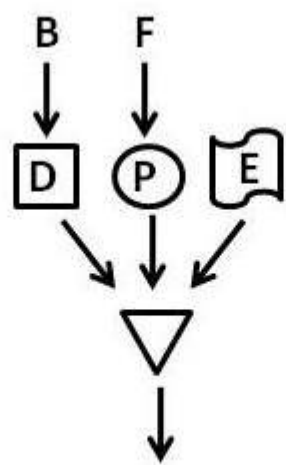

Group DP

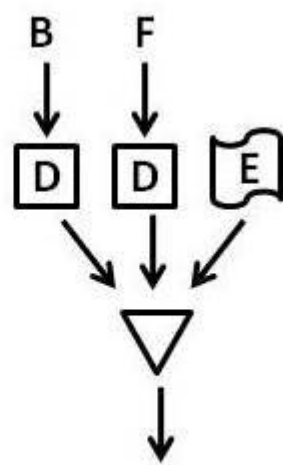

Group D

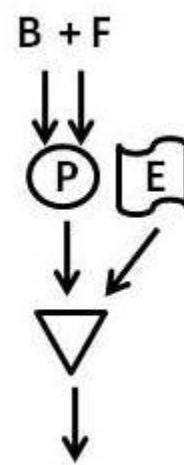

Group P

b)

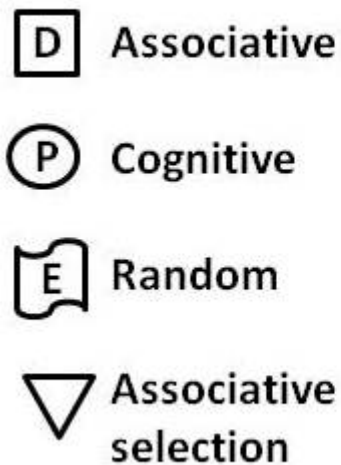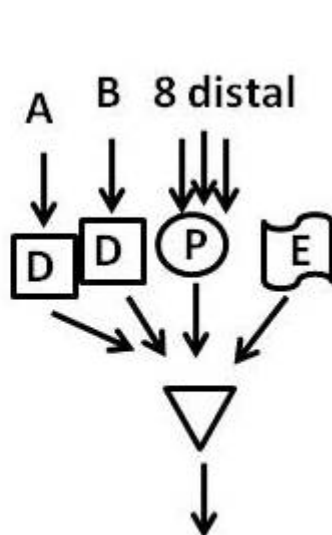

Group DP

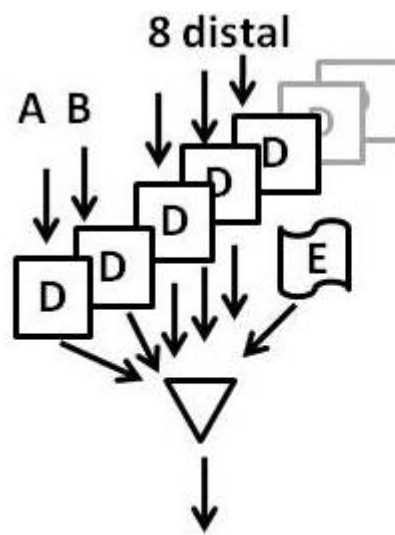

Group D

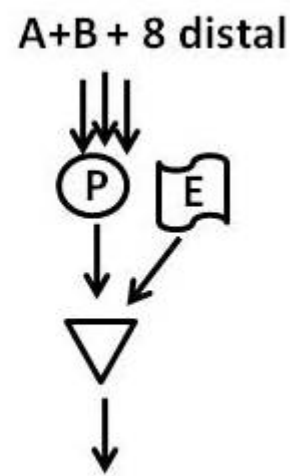

Group P

Supplement: S2 Fig — a) Cue assignment used for Experiment V. b) Cue assignment used for Experiment VI. (PDF) [file pcbi.1006092.s005.pdf]

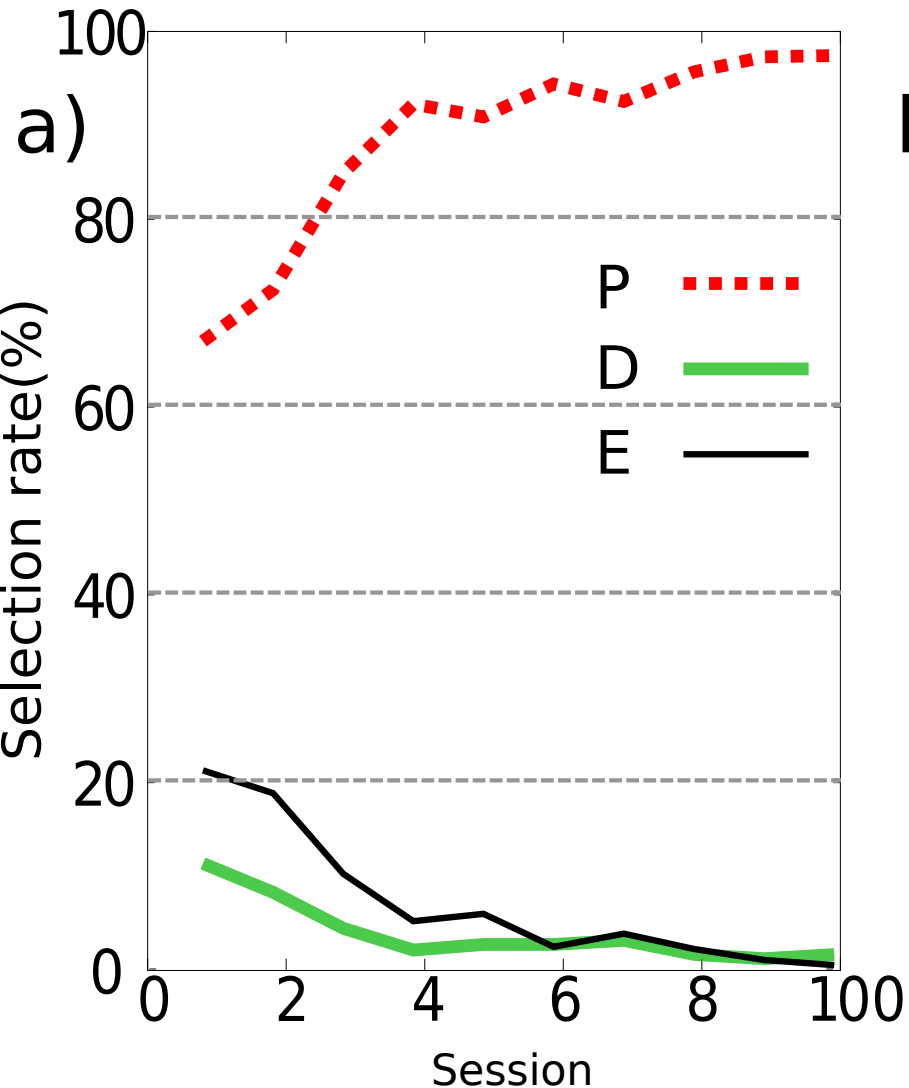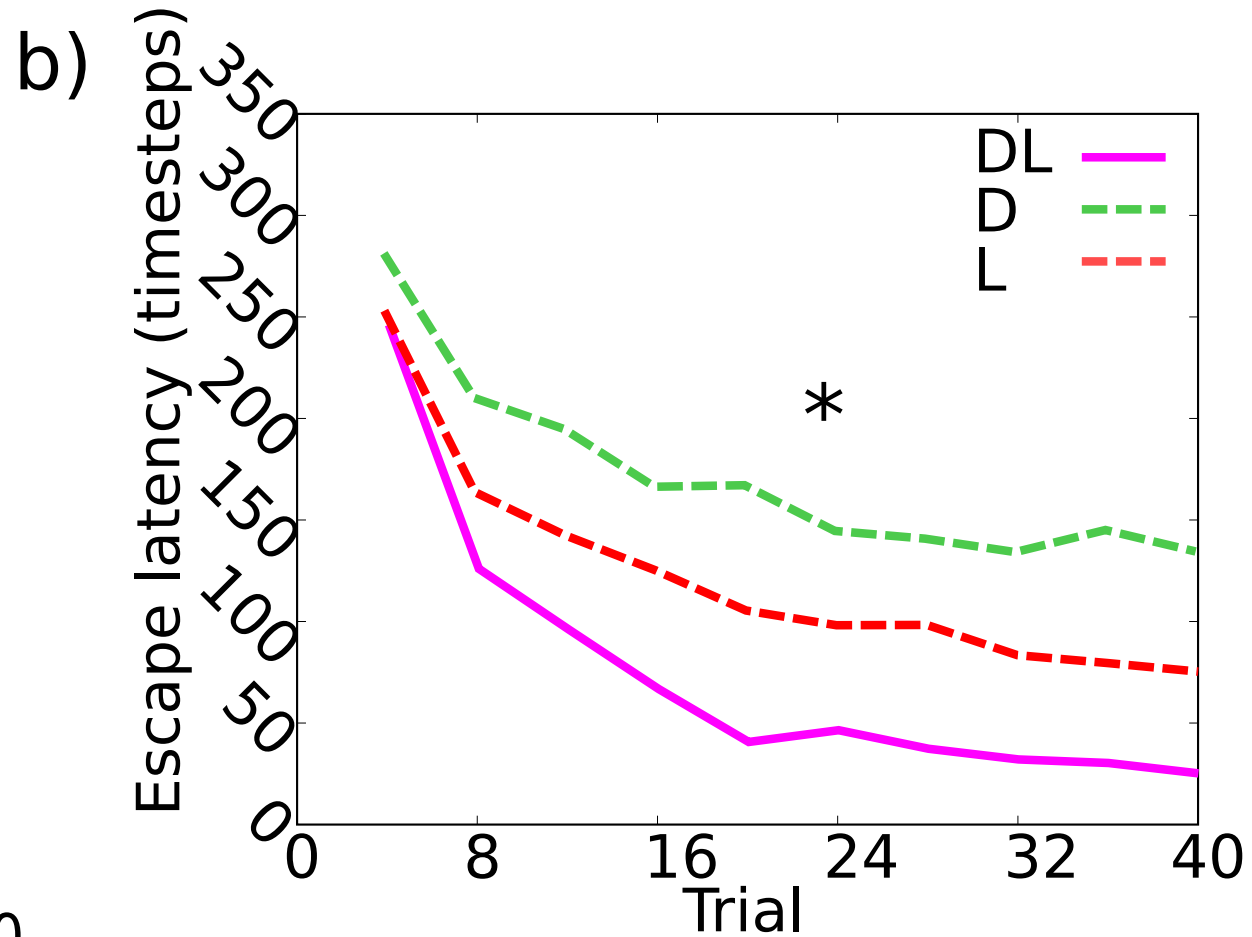

Supplement: S3 Fig — a) Selection rate of the strategies. b) Performance when the model-based Planning strategy in the model is replaced by a model-free Locale strategy. D: Direction Strategy; E: Exploration Strategy; L: Locale Strategy; P: Planning Strategy. (PDF) [file pcbi.1006092.s006.pdf]

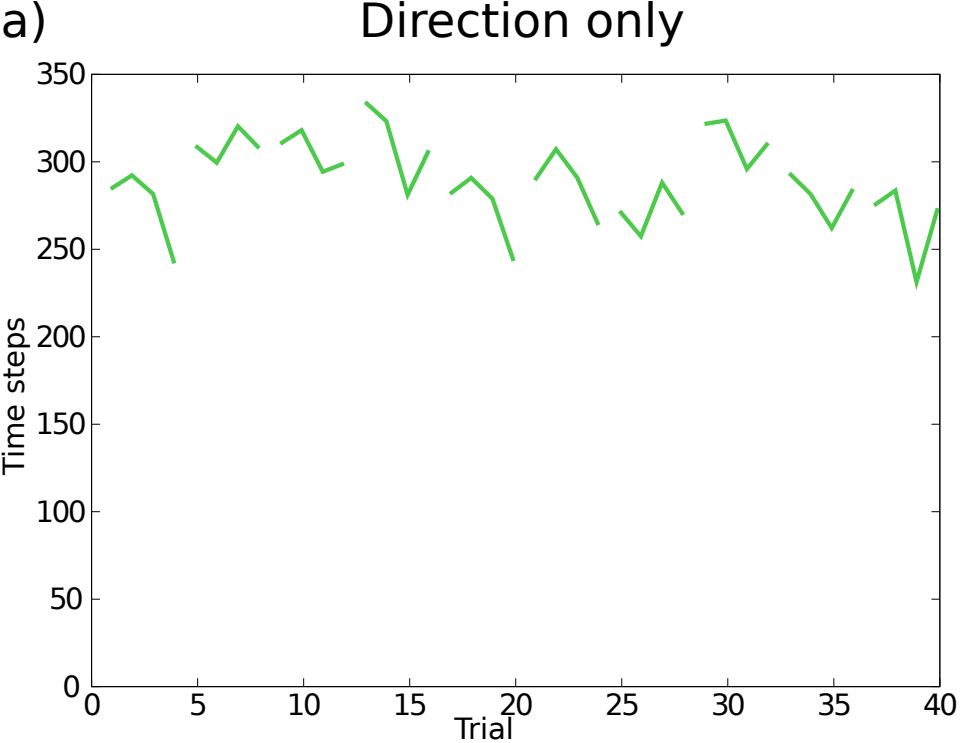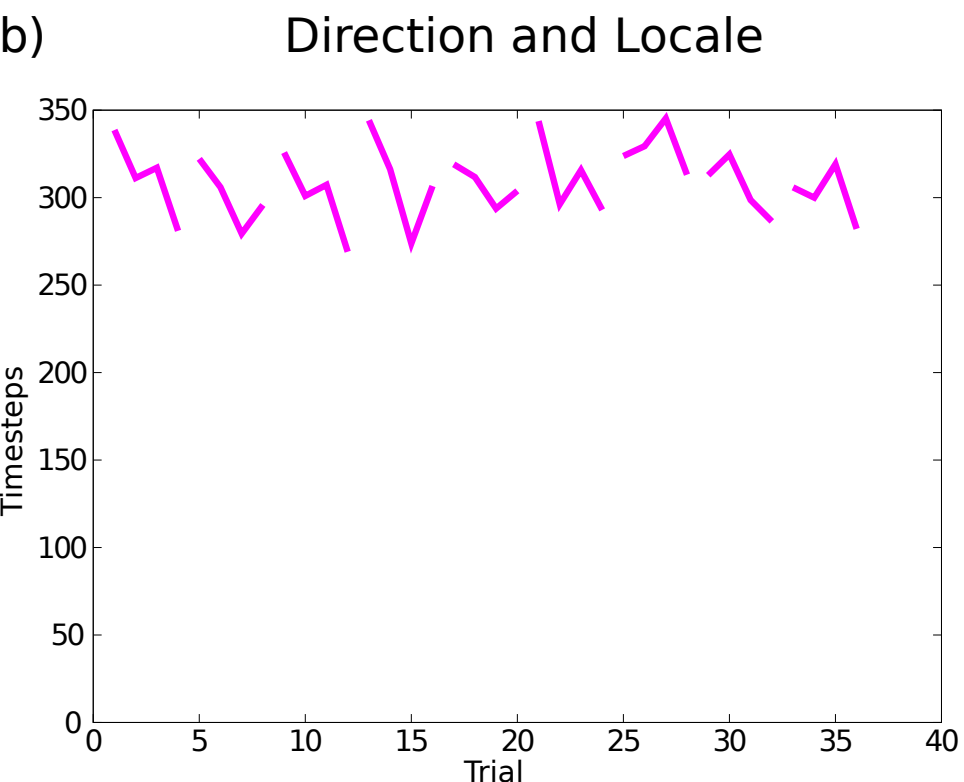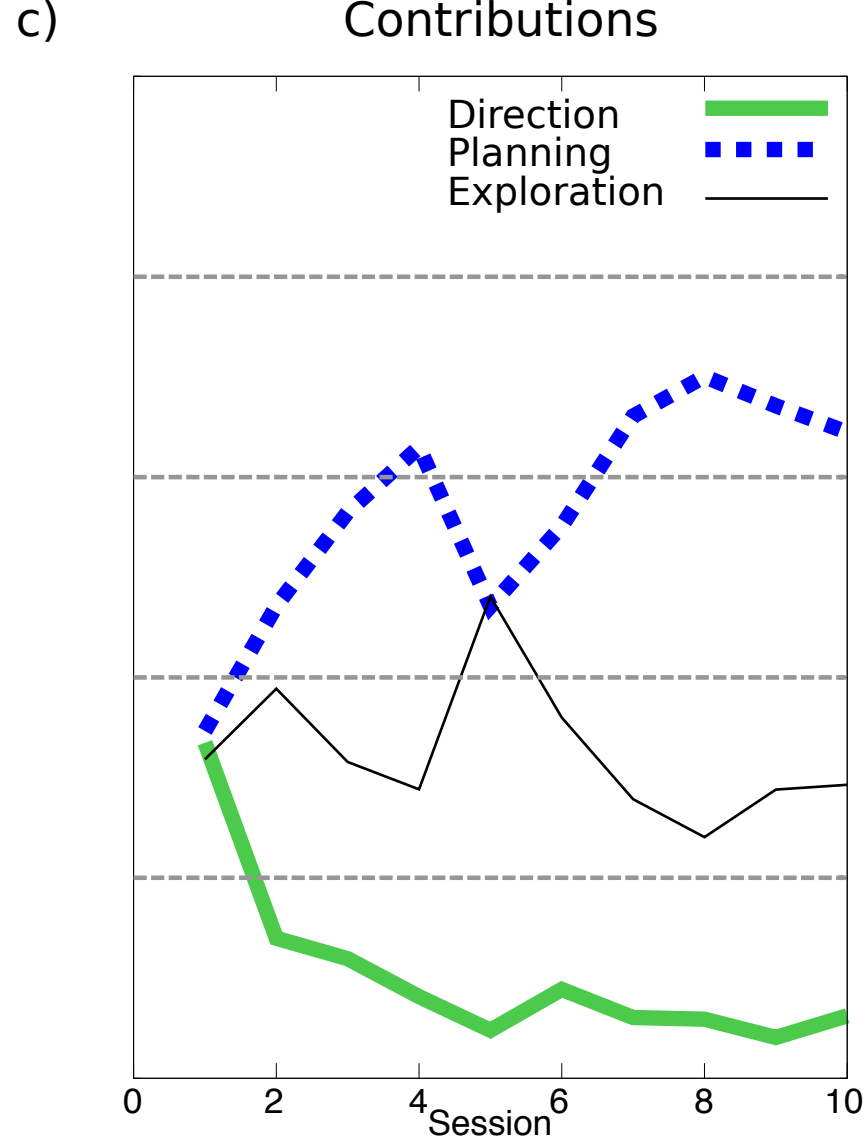

Supplement: S4 Fig — Simulation results with a) the Direction strategy only, b) the Direction and Locale strategies together, c) the full model illustrating the contribution of individual strategies to the behavior of each strategy in terms of % of time where they are selected. (PDF) [file pcbi.1006092.s007.pdf]

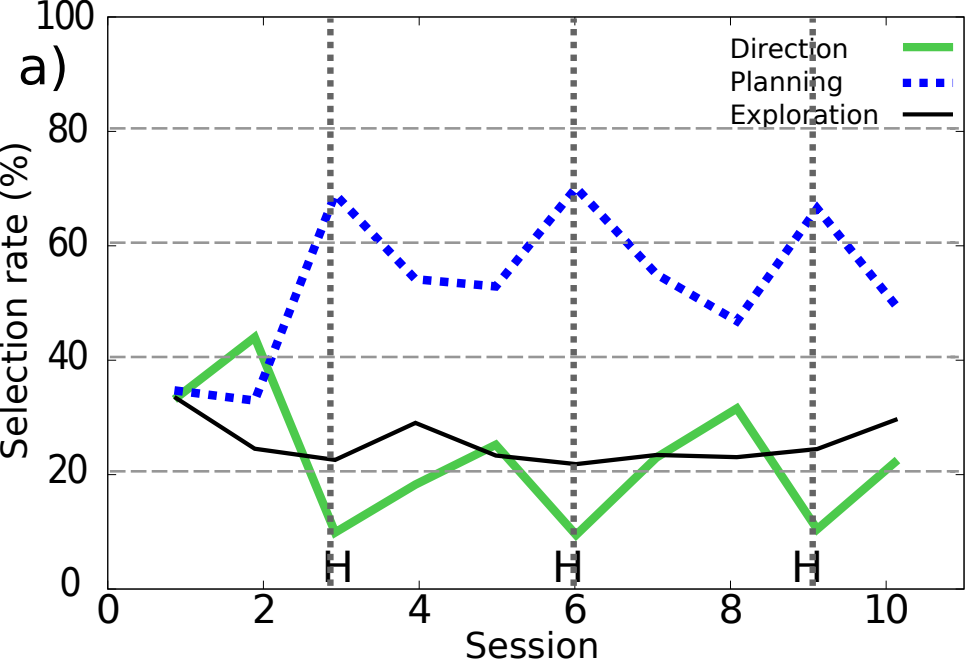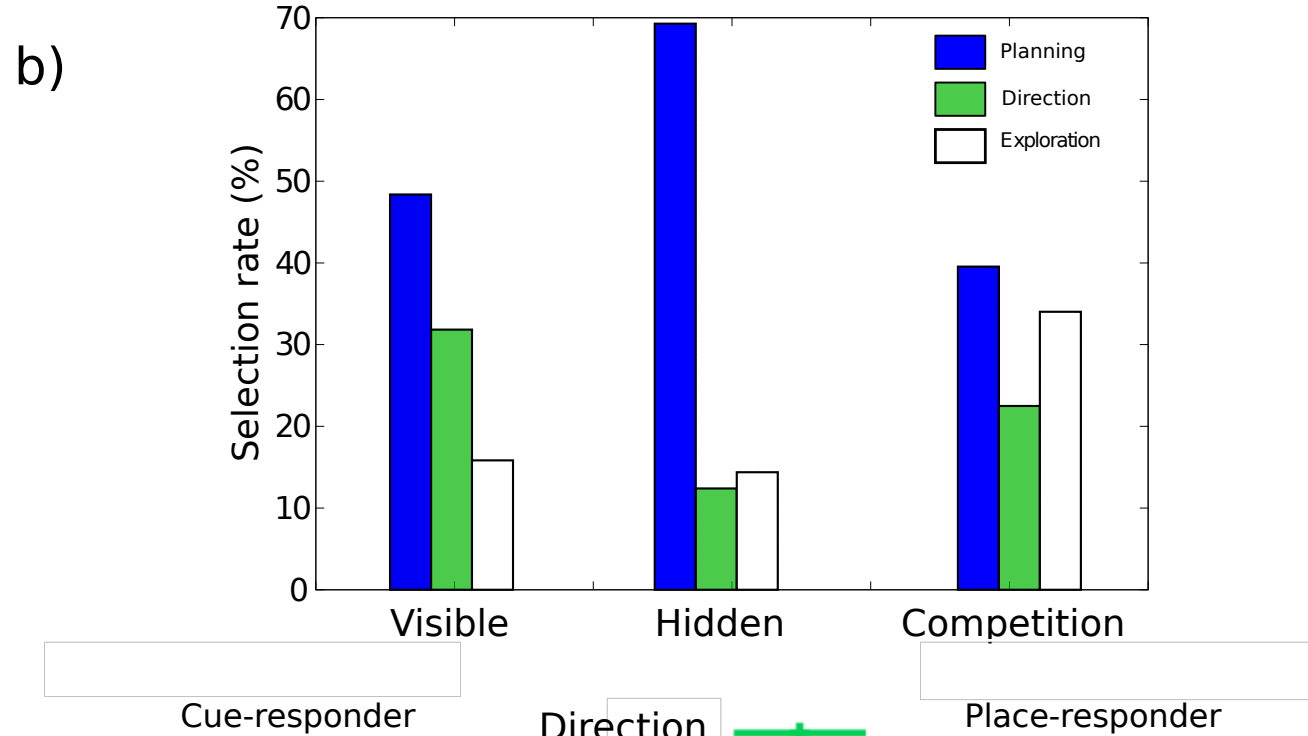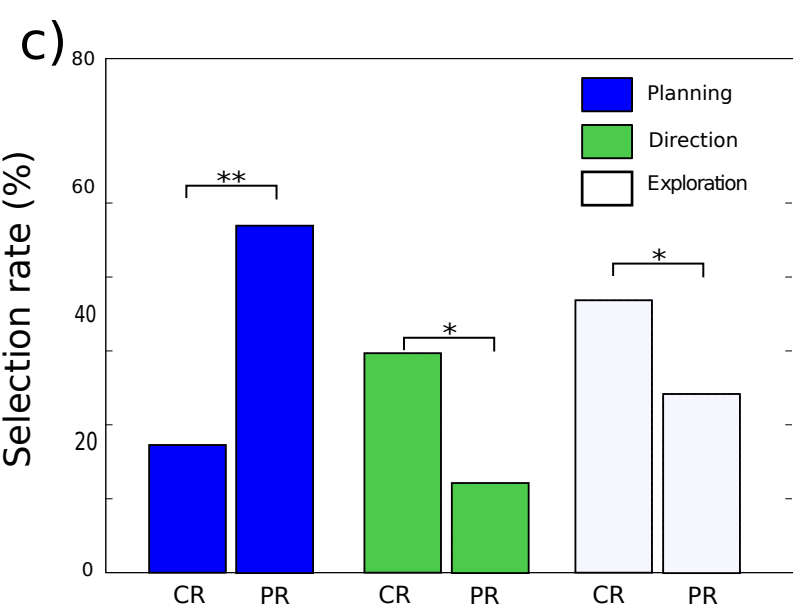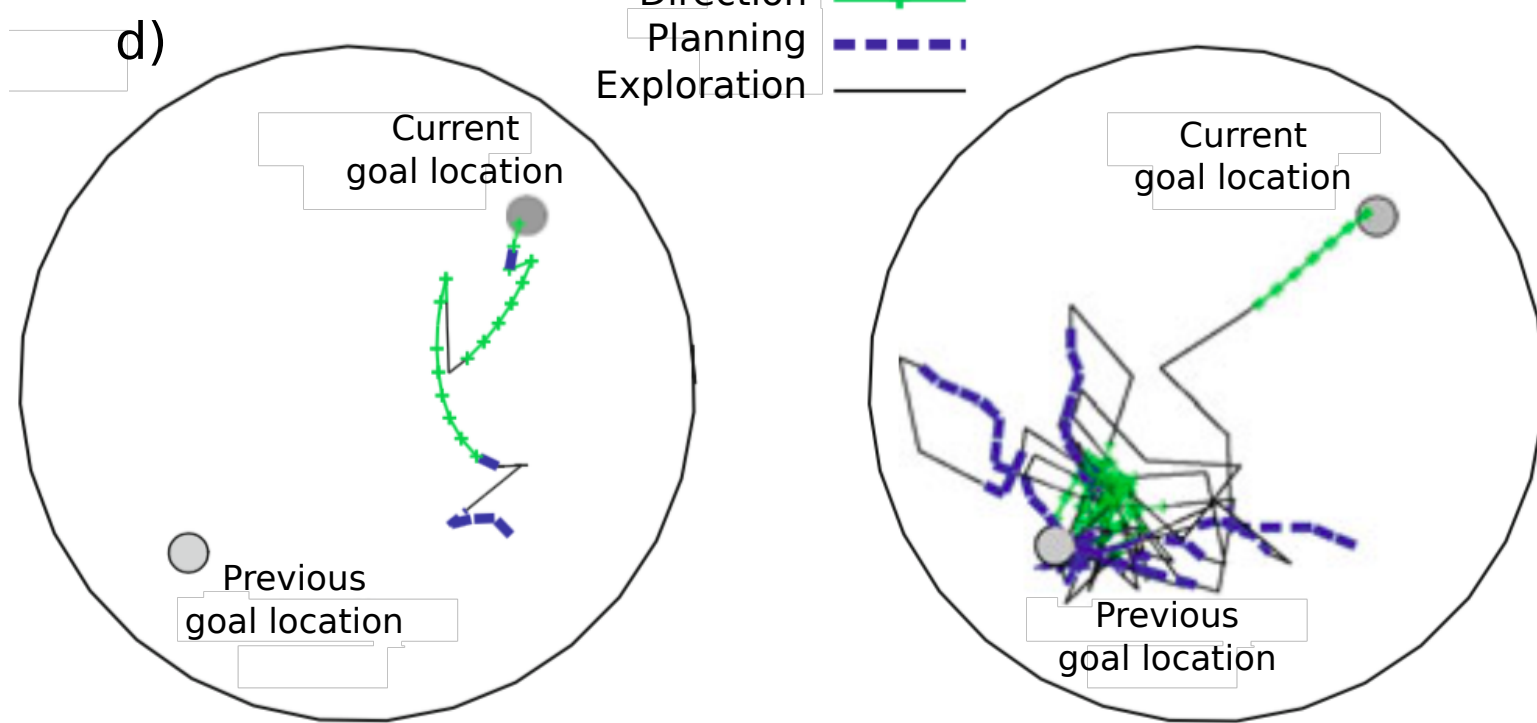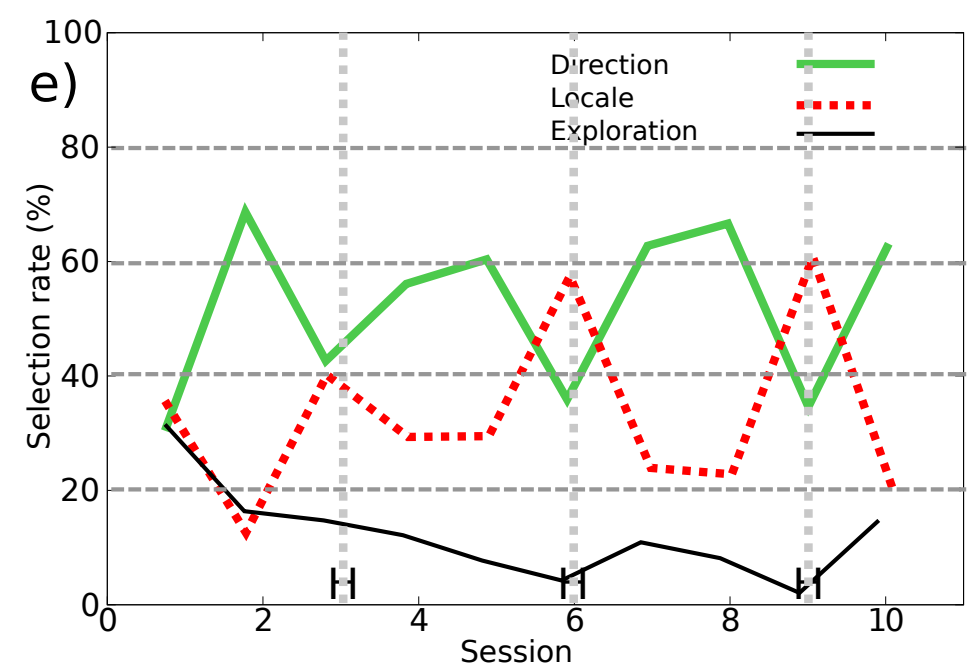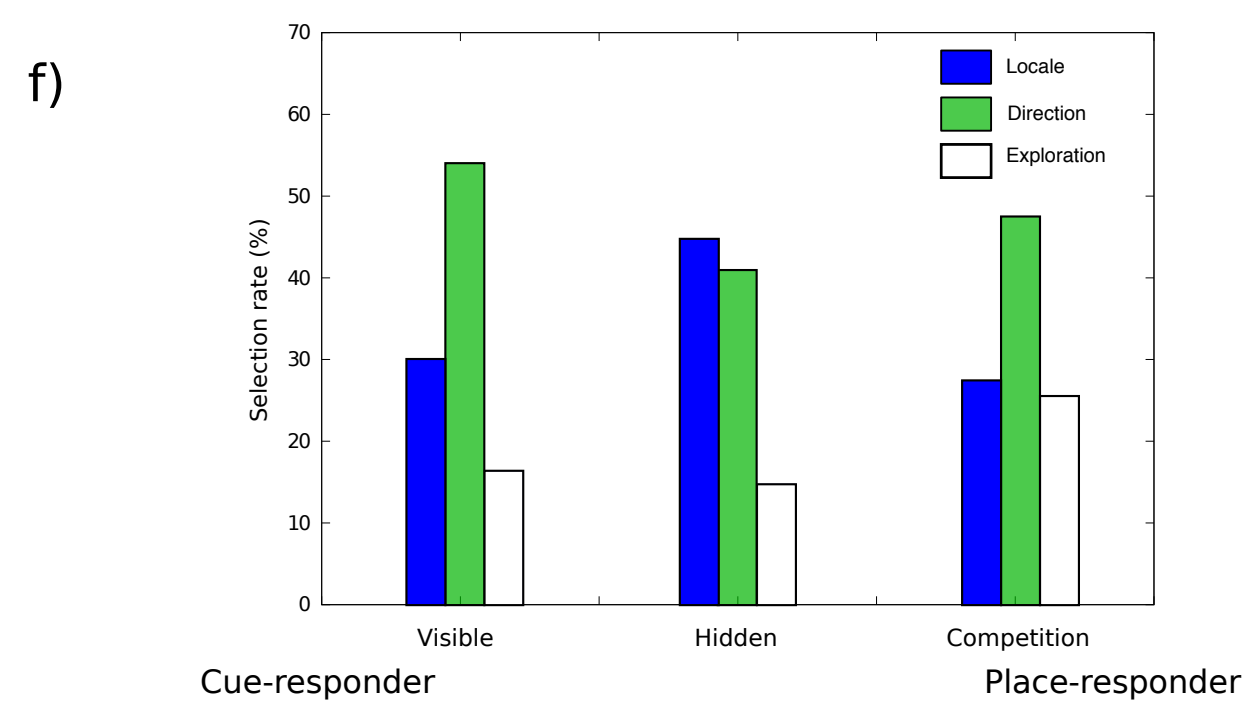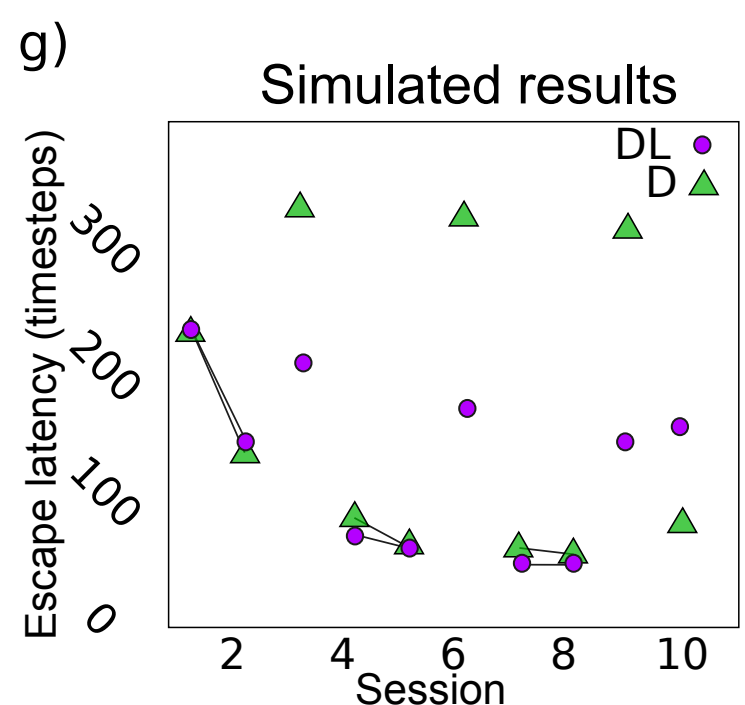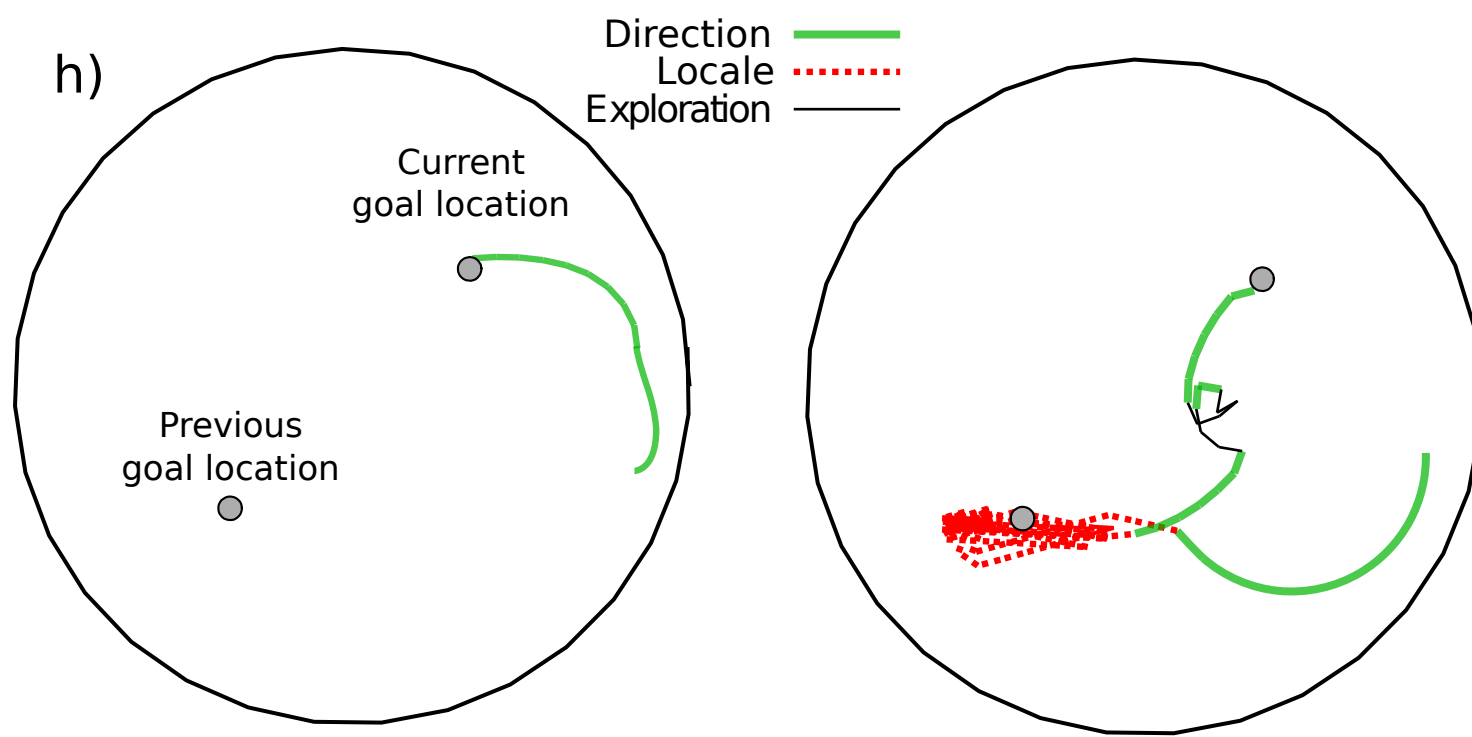

Supplement: S5 Fig — (a-d) Simulation results with the full model (Direction (D) Planning (P) and Exploration (E) strategies together). a) Session-by-session selection rate of strategies. b) Selelection rate of strategies by types of trials. c) Selection rates of strategies by types of simulated animals: Cue Responders (CR) and Place Responders (PR). d) Examples of individual simulated trajectories at the competition trial #10 (adapted from [14]). (e-h) Simulated results when the model-based Planning strategy in the model is replaced by a model-free Locale (L) strategy. e) Session-by-session selection rate of strategies. f) Selection rate of strategies by types of trials. g) Reproduction of the experimental results of [16]. h) Examples of individual simulated trajectories at the competition trial #10. (PDF) [file pcbi.1006092.s008.pdf]

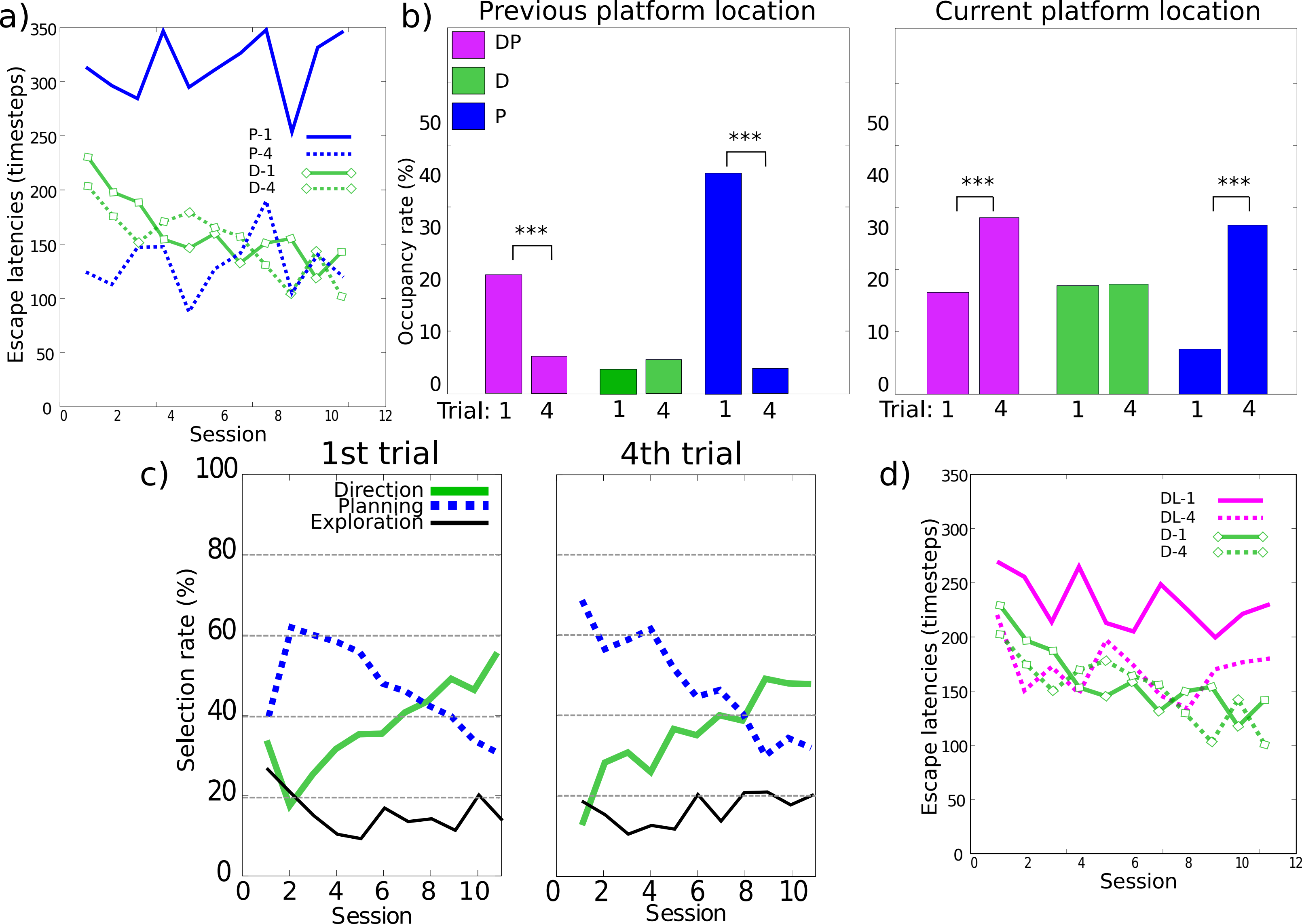

Supplement: S6 Fig — (a-c) Simulation results with the full model (Direction (D) Planning (P) and Exploration (E) strategies together). a) Experimental predictions raised when the hippocampus in the model is lesioned (Group D) versus when when the striatum in the model is lesioned (Group P). b) Occupancy rate in the quadrants containing either the previous or the current platform location at the first and fourth trial of each session. c) Selection rate of each strategy in the full model during the first versus the fourth trial of each session. d) Simulated results when the model-based Planning strategy in the model is replaced by a model-free Locale (L) strategy. (PDF) [file pcbi.1006092.s009.pdf]

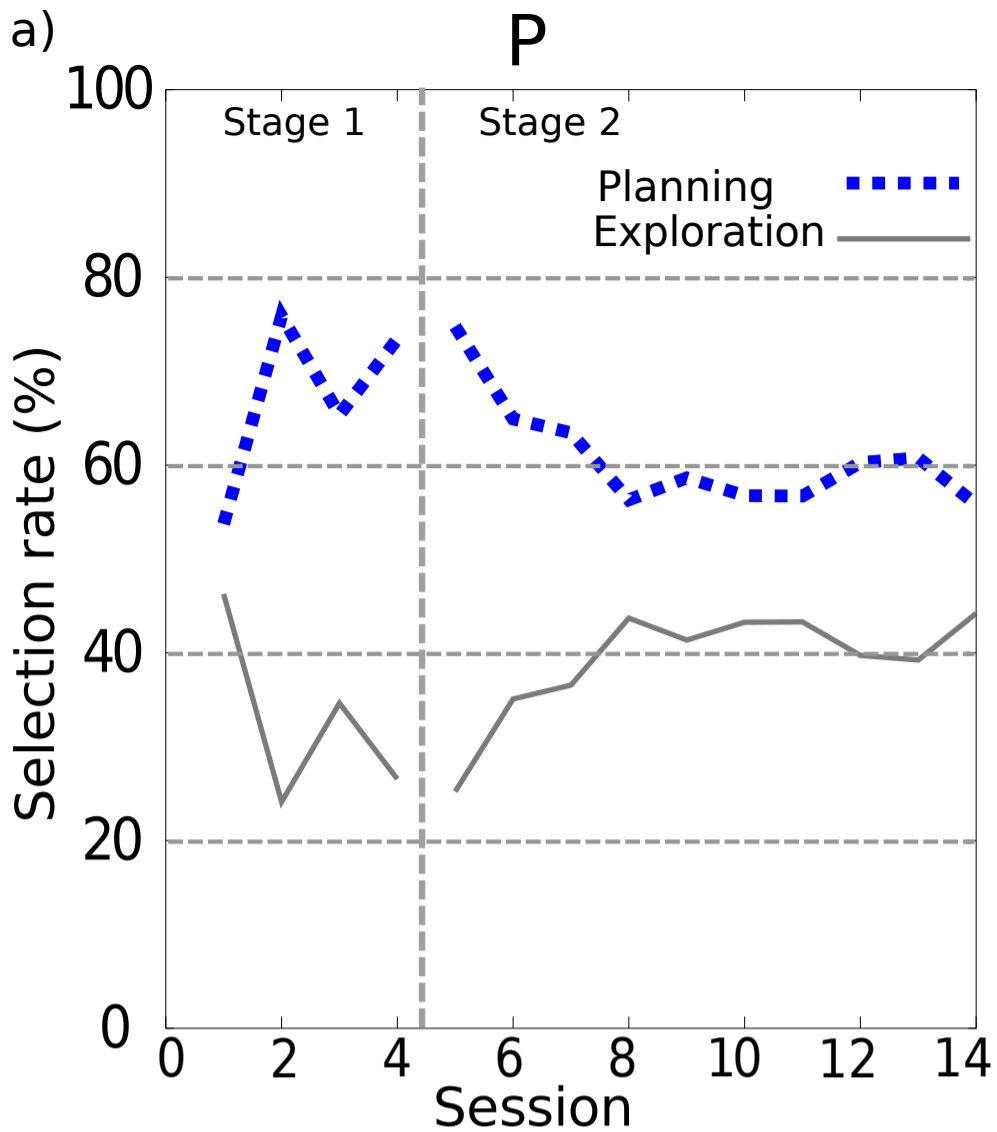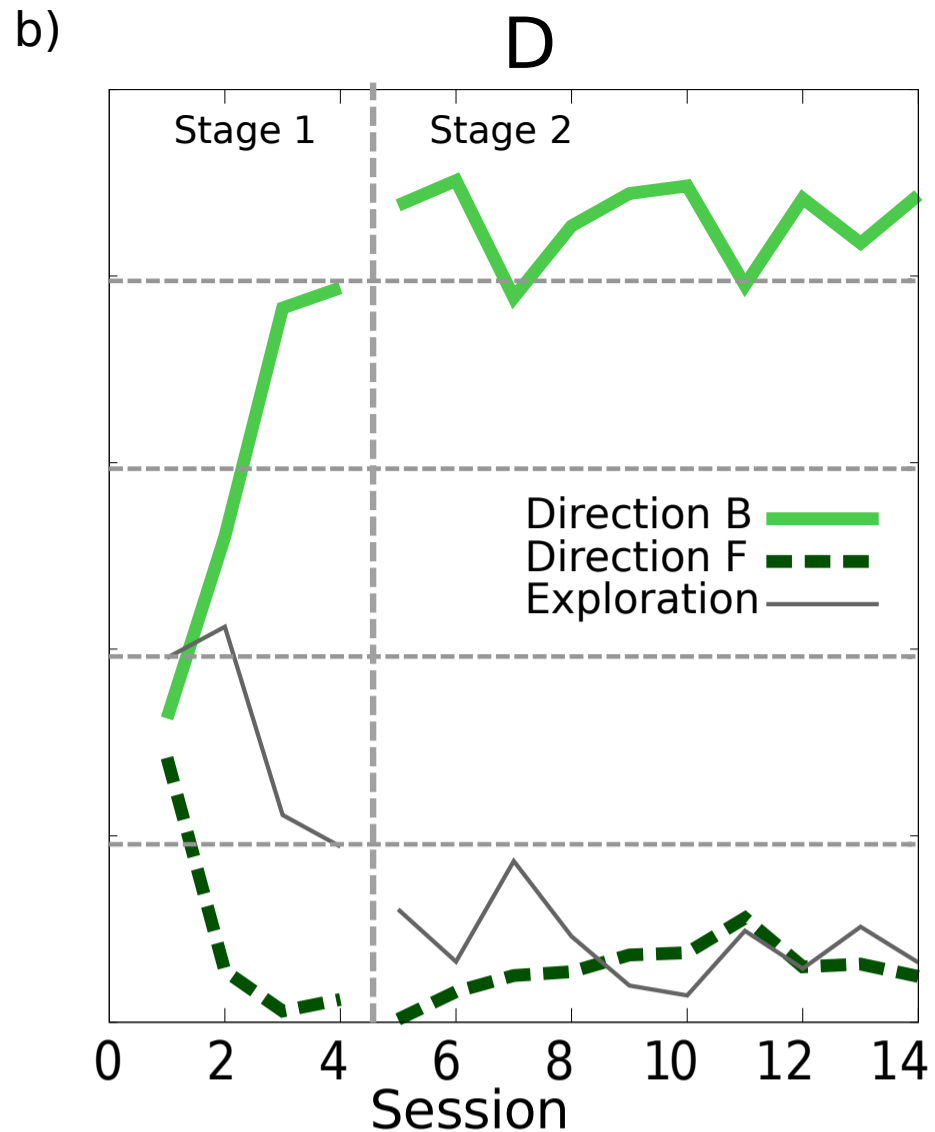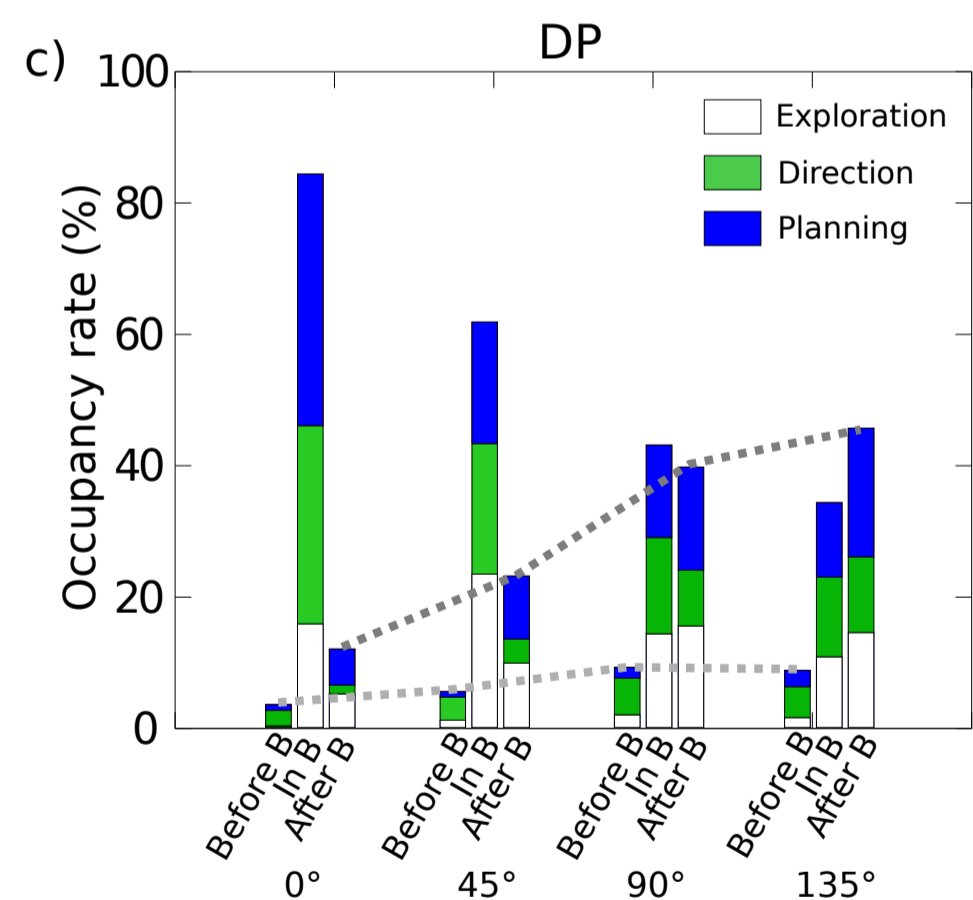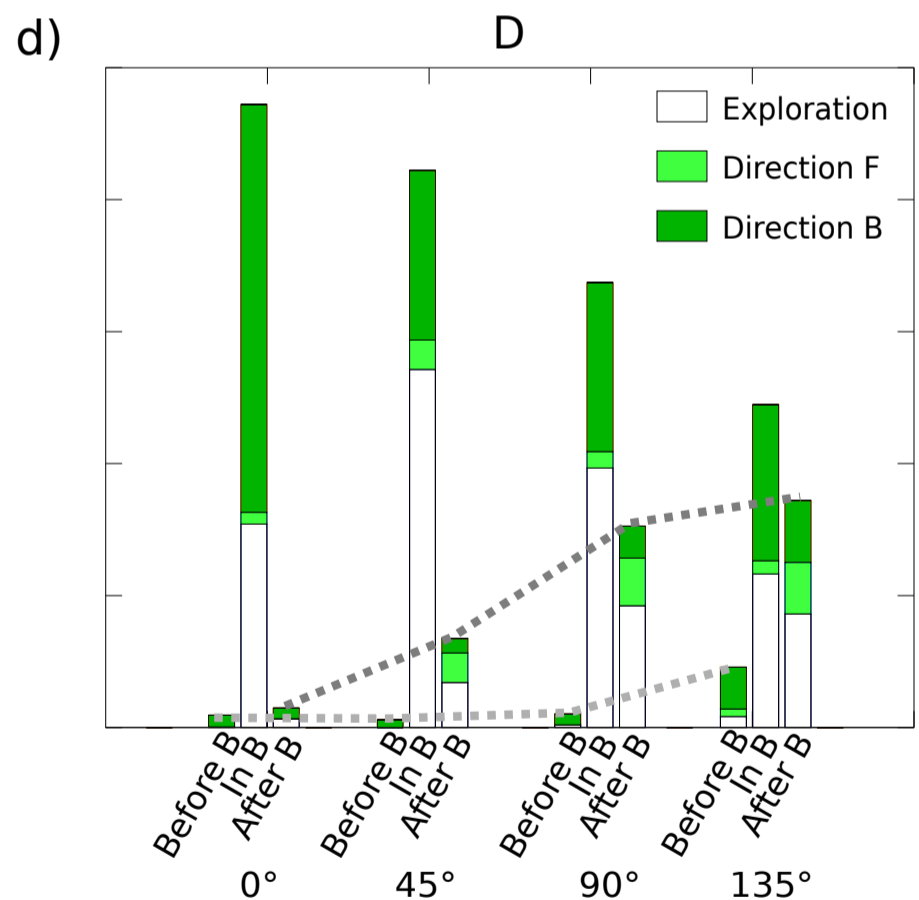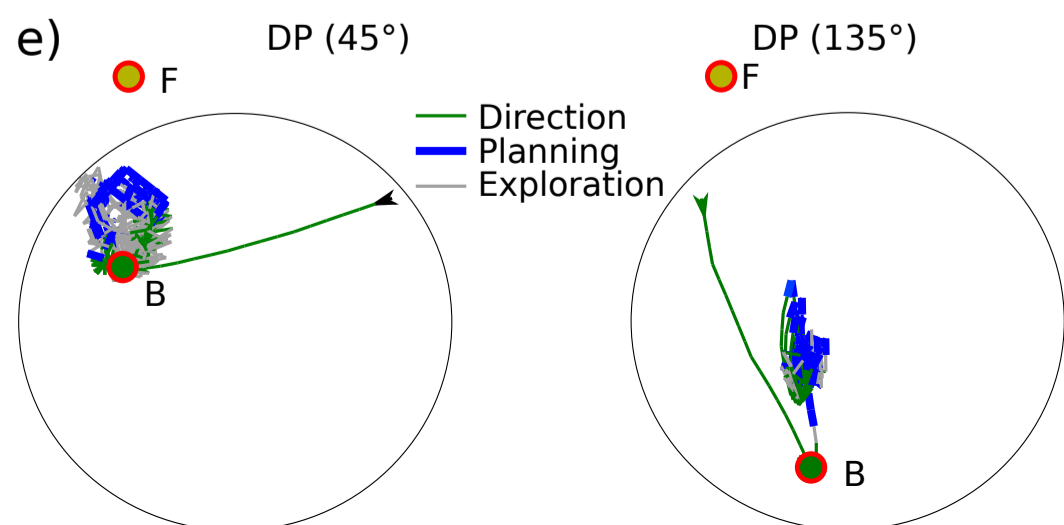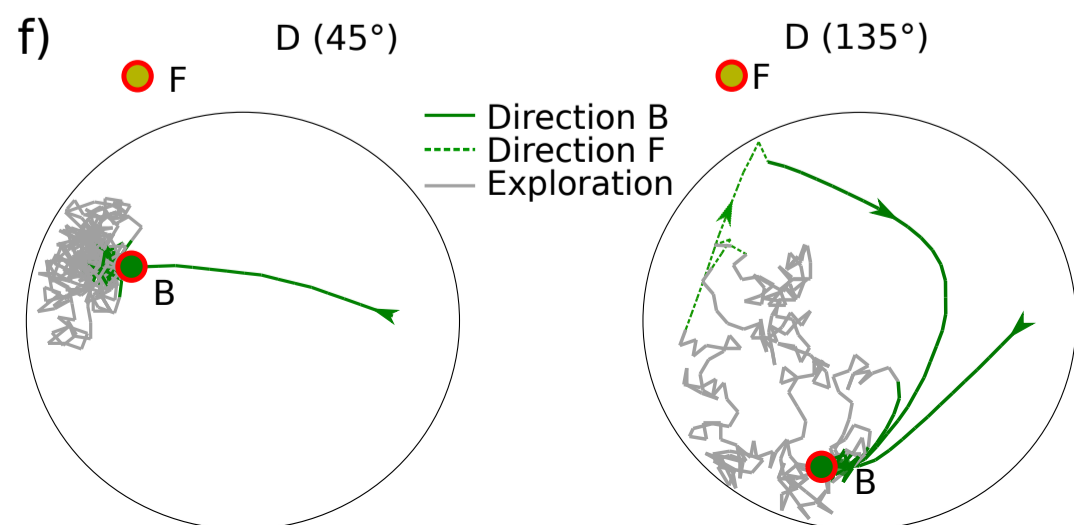

Supplement: S7 Fig — (a-b) Selection rate of strategies during Stage 1 and Stage 2 in groups P (left) and D (right). (c-d) Comparison of the occupancy rate during test trials between Octant B, Before reaching Octant B, and After reaching Octant B for groups DP (left) and D (right). Within each octant is also shown the selection rate of strategies that contributed to this occupancy pattern. (e-f) Examples of typical trajectories of groups DP (left) and D (right) for tests 45° and 135°. (PDF) [file pcbi.1006092.s010.pdf]

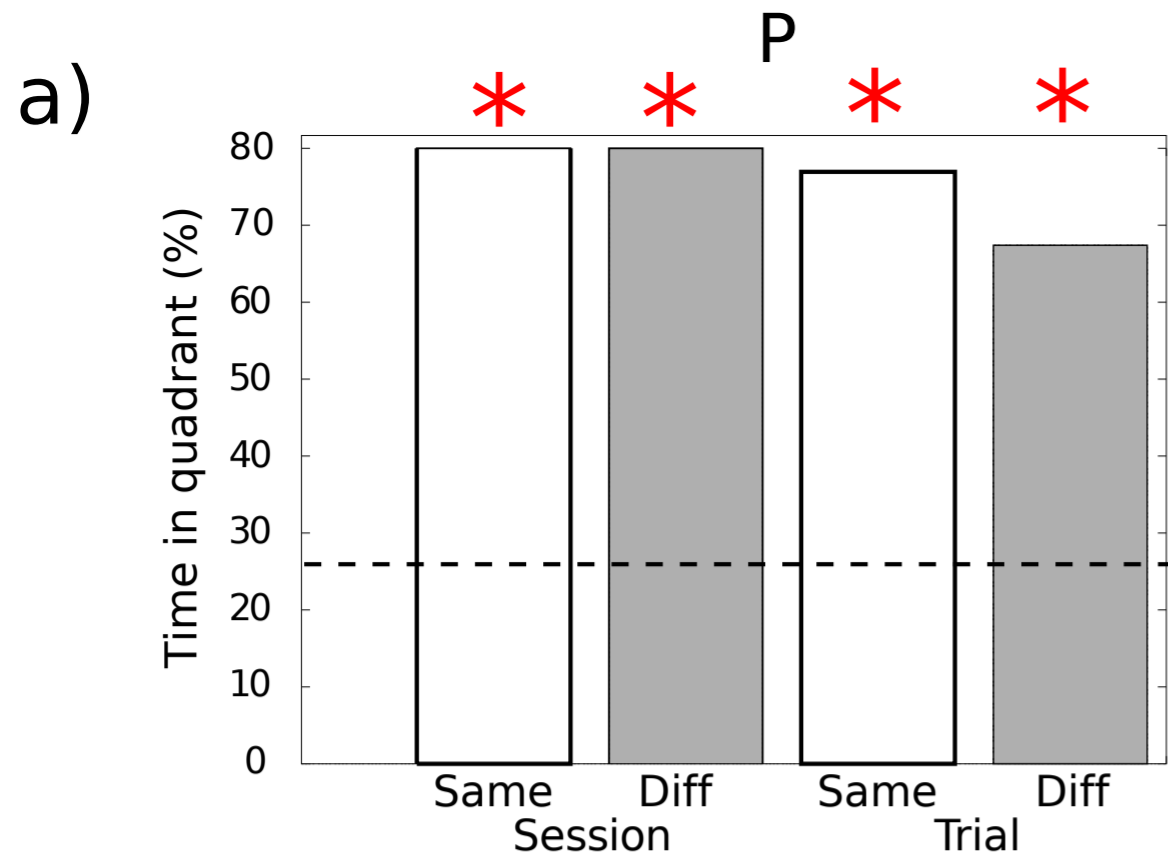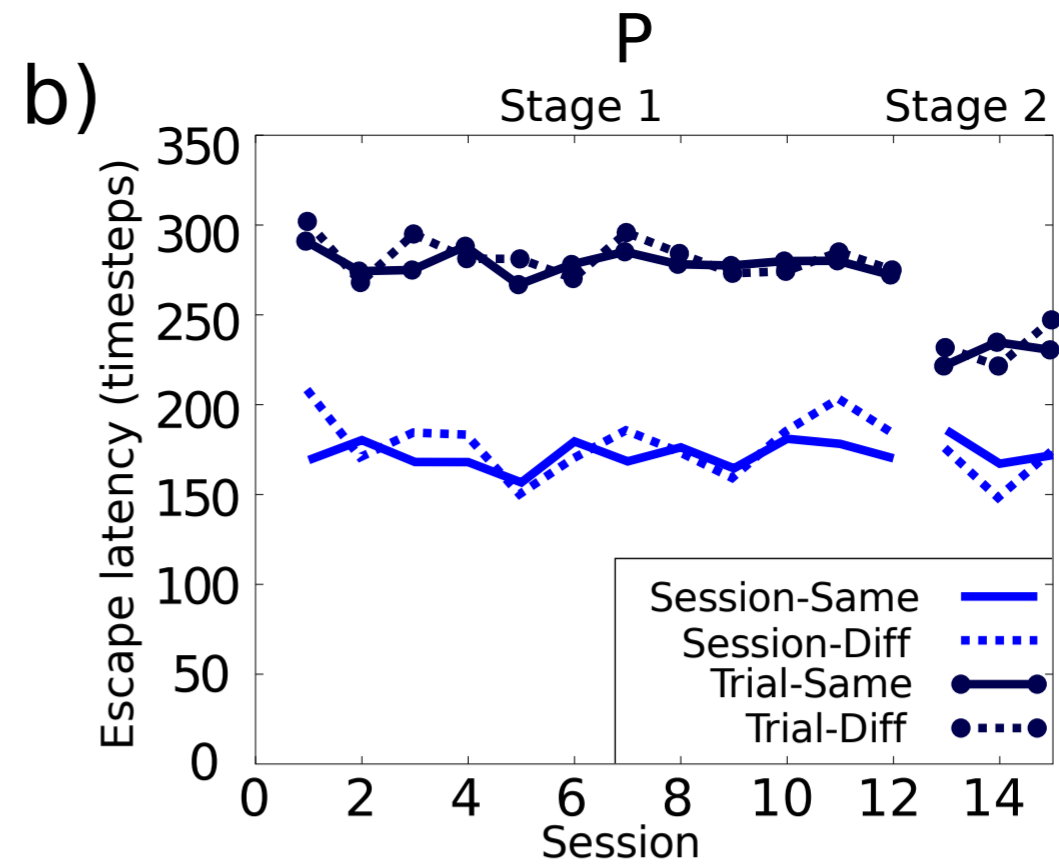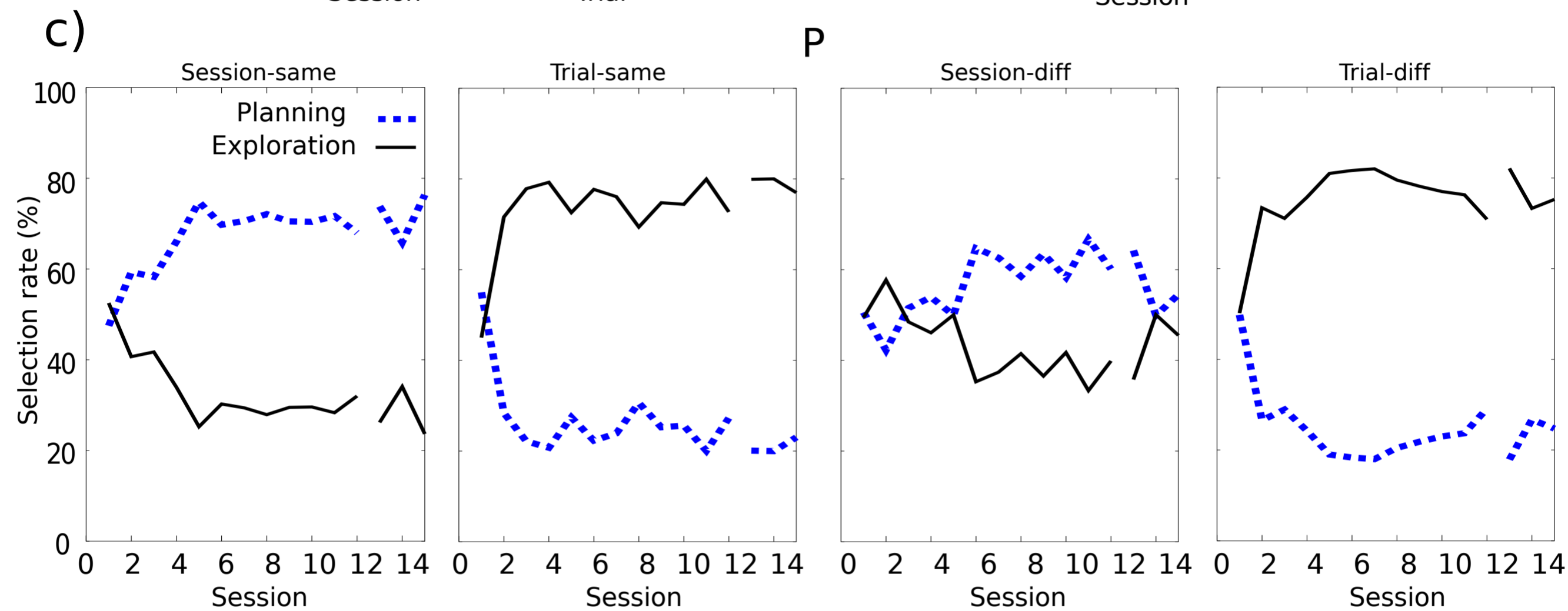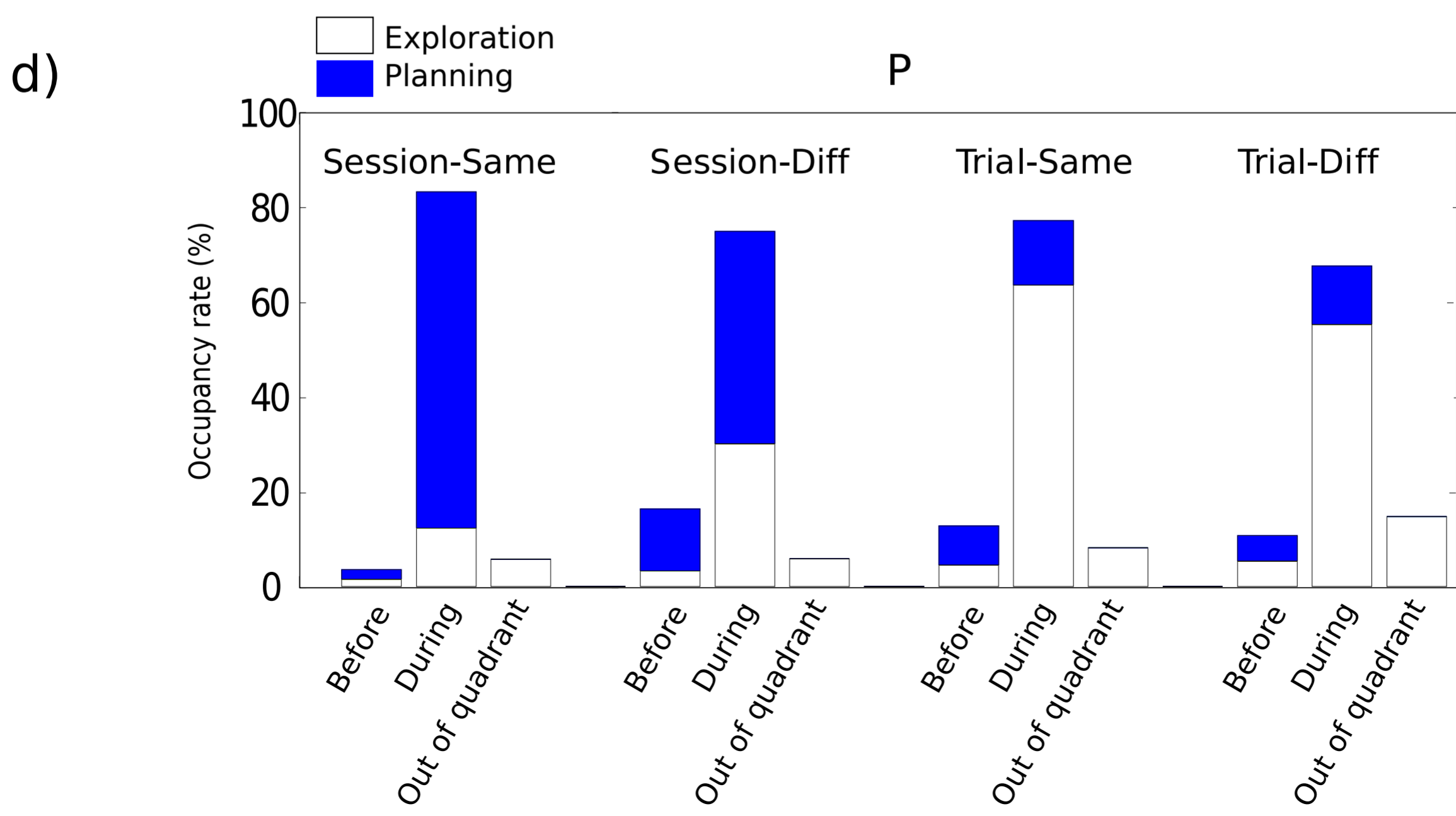

Supplement: S8 Fig — Results with a model only employing the Planning (P) strategy combined with an Exploration strategy. a) The time spent in the quadrant containing the previous platform location is significantly above chance (dashed line) in all conditions, unlike experimental results. b) Escape latencies during Stage 2 do not show an improvement and are significantly different between conditions Trial and Session, unlike experimental results. c) Selection rate of each strategy in each condition of the task underlying the behavior of the P model. d) Details of strategy selection for model P during the test trial of Stage 3, before reaching for the first time the goal quadrant (first column) and after, during the occupancy of the goal quadrant (second column) and of the others (third column). (PDF) [file pcbi.1006092.s011.pdf]

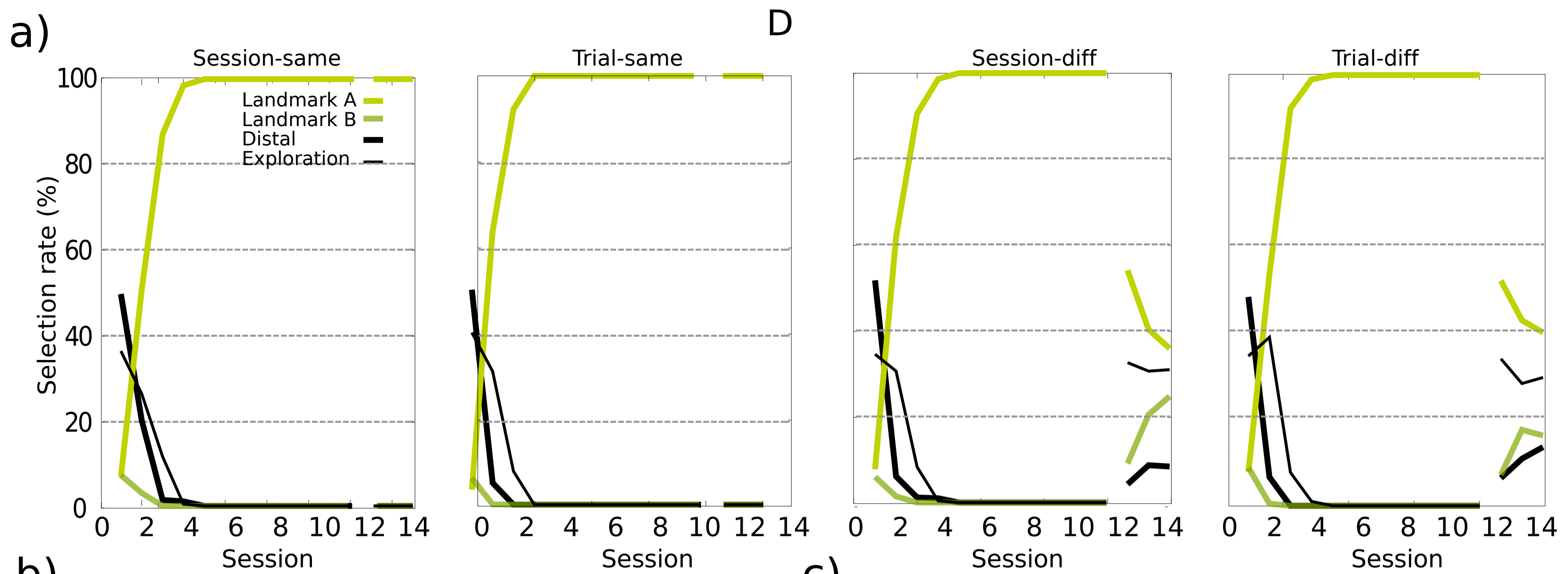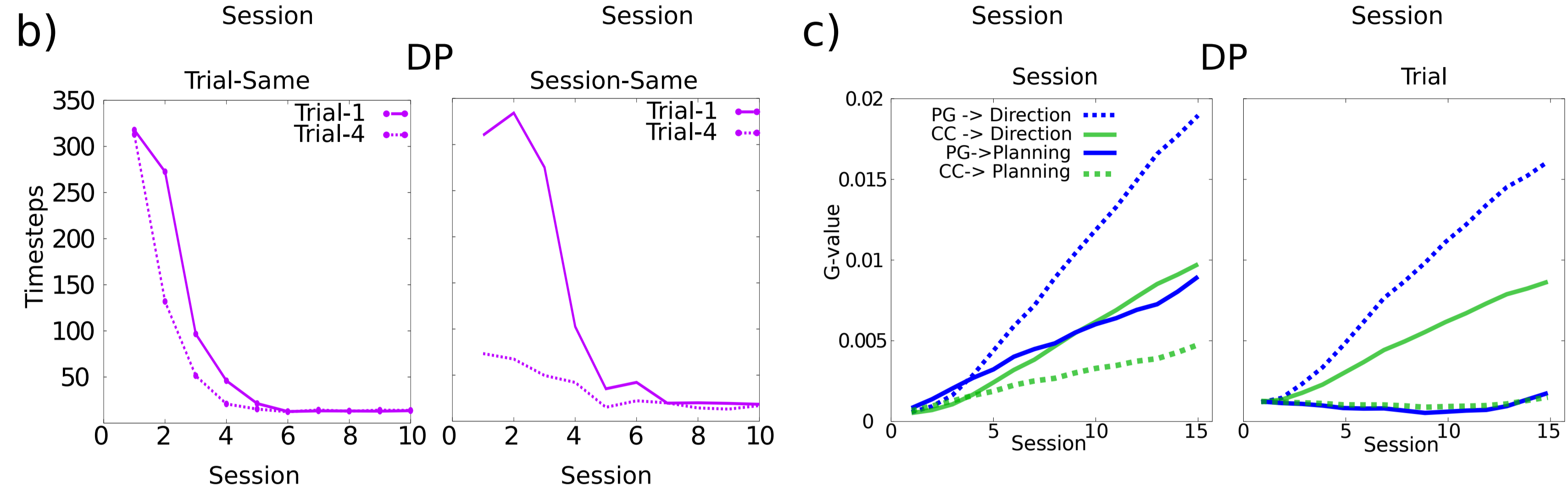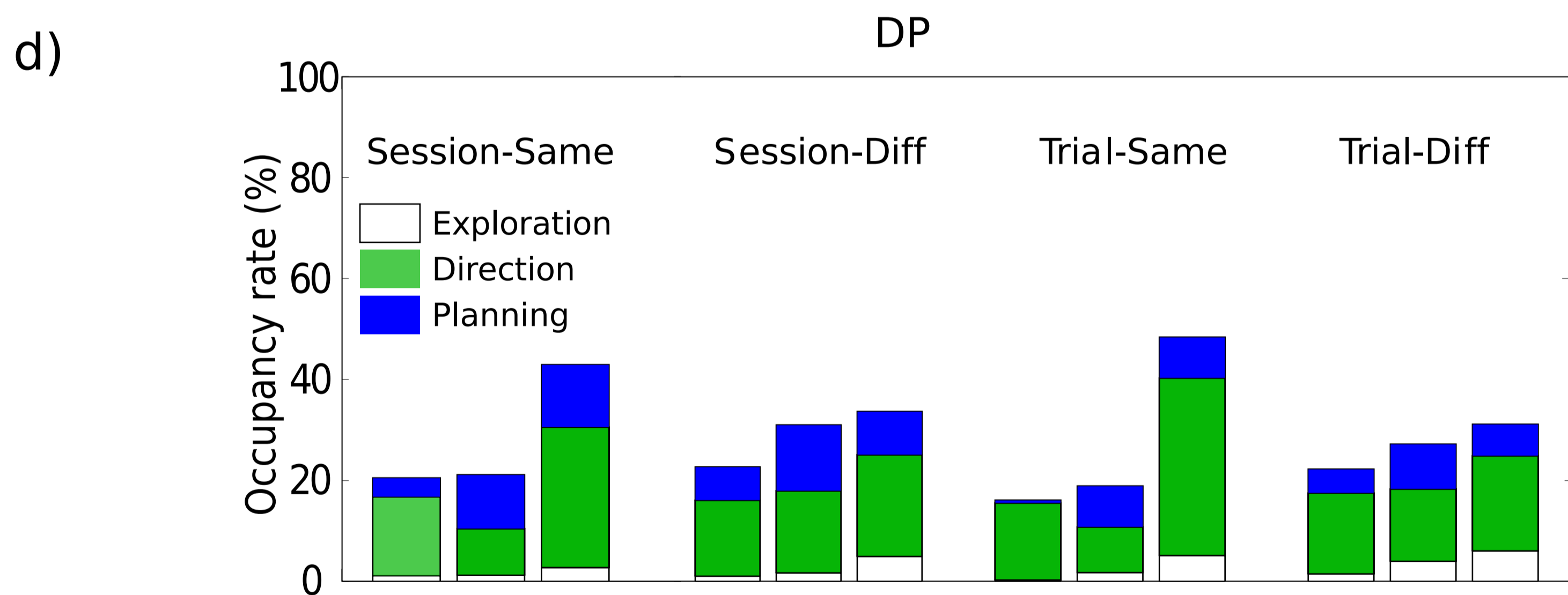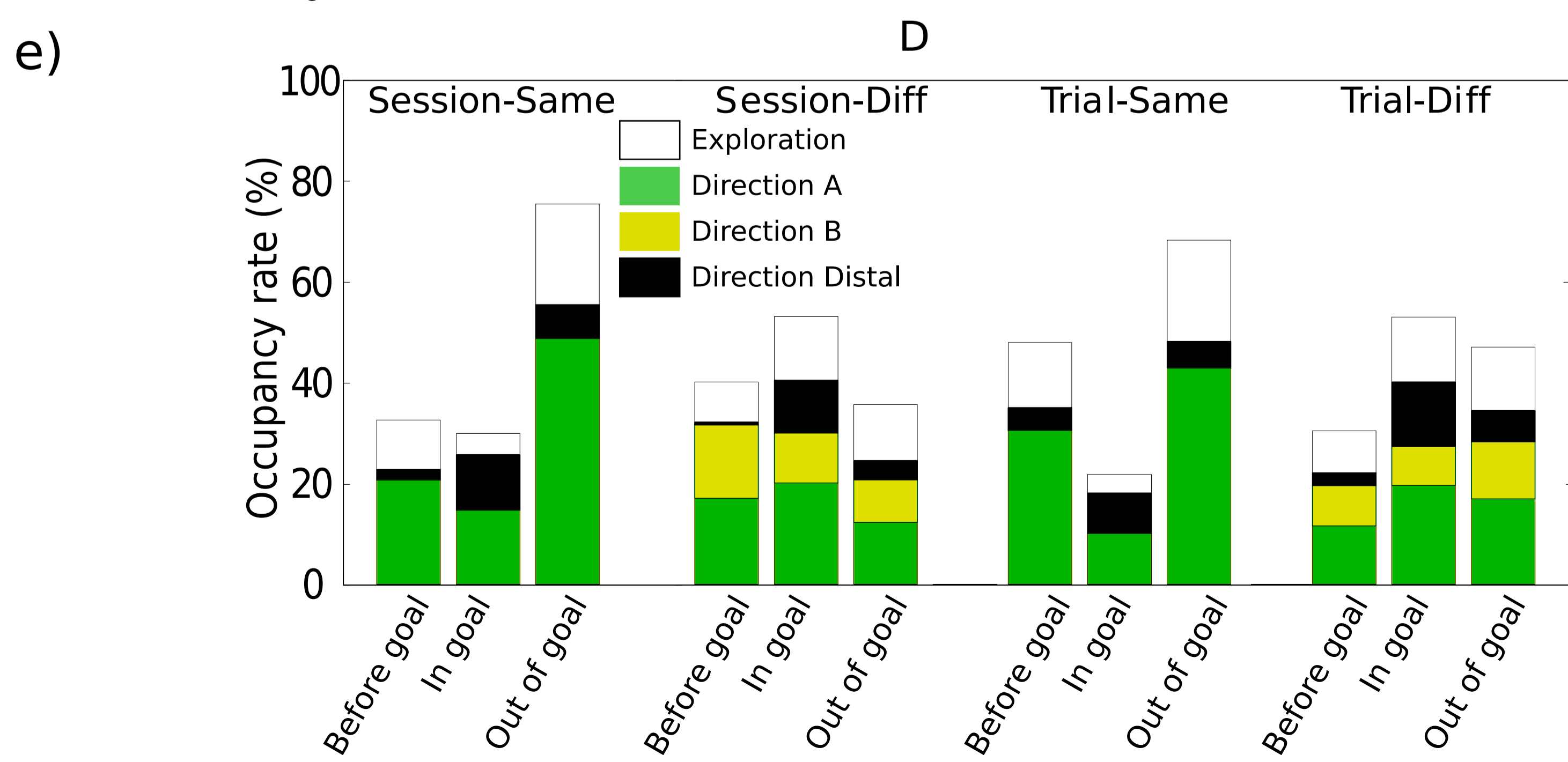

Supplement: S9 Fig — Detailed results with the full DP model and the D model. a) Selection rate of each strategy in each condition of the task for the D model. b) Performance of group DP (Trial-Same and Session-Same) during the first trial and the fourth trial of a session during Stage 1. c) Evolution of weights in the gating network between the inputs and their dedicated strategies units for Session-Same and Trial-Same conditions of the DP model. (d-e) Selection rates of strategies in Session-Same, Session-Diff, Trial-Same and Trial-Diff conditions of the DP model (d) and the D model (e) during Stage 1 and Stage 2. (PDF) [file pcbi.1006092.s012.pdf]
